# Synergy of Electrostatic and π–π Interactions in the Realization of Nanoscale Artificial Photosynthetic Model Systems

**DOI:** 10.1002/anie.202006014

**Published:** 2020-09-02

**Authors:** Eduardo Anaya‐Plaza, Jan Joseph, Stefan Bauroth, Maximilian Wagner, Christian Dolle, Michael Sekita, Franziska Gröhn, Erdmann Spiecker, Timothy Clark, Andrés de la Escosura, Dirk M. Guldi, Tomás Torres

**Affiliations:** ^1^ Department of Organic Chemistry Universidad Autónoma de Madrid (UAM) c/ Francisco Tomás y Valiente 7, Cantoblanco 28049 Madrid Spain; ^2^ Department of Bioproducts and Biosystems Aalto University Kemistintie 1 02150 Espoo Finland; ^3^ Department of Chemistry and Pharmacy & Interdisciplinary Center for Molecular Materials (ICMM) Friedrich-Alexander-Universität Erlangen-Nürnberg (FAU) 91058 Erlangen Germany; ^4^ Institute for Advanced Research in Chemical Sciences (IAdChem) Universidad Autónoma de Madrid (UAM) 28049 Madrid Spain; ^5^ IMDEA-Nanociencia c/ Faraday 9, Campus de Cantoblanco 28049 Madrid Spain

**Keywords:** artificial photosynthesis, fullerenes, nanoscale self-assembly, phthalocyanines

## Abstract

In the scientific race to build up photoactive electron donor‐acceptor systems with increasing efficiencies, little is known about the interplay of their building blocks when integrated into supramolecular nanoscale arrays, particularly in aqueous environments. Here, we describe an aqueous donor‐acceptor ensemble whose emergence as a nanoscale material renders it remarkably stable and efficient. We have focused on a tetracationic zinc phthalocyanine (ZnPc) featuring pyrenes, which shows an unprecedented mode of aggregation, driven by subtle cooperation between electrostatic and π–π interactions. Our studies demonstrate monocrystalline growth in solution and a symmetry‐breaking intermolecular charge transfer between adjacent ZnPcs upon photoexcitation. Immobilizing a negatively charged fullerene (C_60_) as electron acceptor onto the monocrystalline ZnPc assemblies was found to enhance the overall stability, and to suppress the energy‐wasting charge recombination found in the absence of C_60_. Overall, the resulting artificial photosynthetic model system exhibits a high degree of preorganization, which facilitates efficient charge separation and subsequent charge transport.

## Graphical abstract

The synergy between different non‐covalent interactions is modulated to achieve supramolecular hybrids as electron donor‐acceptor photoactive systems consisting of a tetracationic zinc(II) phthalocyanine and anionic water‐soluble fullerenes. In the resulting monocrystalline assemblies, all the fundamental processes of natural photosynthesis are operative and give rise to highly efficient artificial photosynthetic systems.

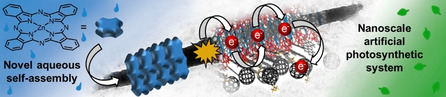

## Introduction

Natural photosynthesis is a key, highly sophisticated biological process that allows the conversion of light into chemical energy.^1^ The development of artificial photosynthetic systems attempts to mimic photosynthesis at the molecular level and to understand the interplay between fundamental processes such as light harvesting, energy transfer, and charge transfer.^2^, ^3^, ^4^, ^5^, ^6^, ^7^

A rather elegant and simple approach to modeling natural antennae involves the use of either electron donors or acceptors that feature high extinction coefficients throughout the visible region of the solar spectrum.^8^, ^9^, ^10^, ^11^ Electron donor‐acceptor systems are equally important as models to mimic the events that take place in the photosynthetic reaction center.^12^, ^13^, ^14^, ^15^ Both are of utmost relevance for the modulation of solar energy conversion and optoelectronic materials.

Phthalocyanines (Pc) and fullerenes (C_60_) stand out among the many molecular building blocks employed in artificial photosynthetic models. Pcs are thermally and chemically stable, feature a rich redox chemistry, and are tunable through coordination of different metals and/or peripheral/axial substitution.^16^, ^17^, ^18^, ^19^, ^20^ Moreover, their intense optical absorption in the red region of the solar spectrum renders them as excellent antennae. Such unique physicochemical properties have provided the incentive to incorporate Pcs into a large number of electron donor‐acceptor systems using both covalent and non‐covalent methodologies.^21^, ^22^, ^23^, ^24^, ^25^, ^26^ The role of Pcs is to harvest light efficiently and, once photoexcited, to act as electron donors. The choice of C_60_ as electron acceptor, in turn, is mainly driven by the fact that it exhibits small reorganization energies in charge‐transfer reactions.^27^, ^28^, ^29^ Non‐covalent approaches en route to electron donor‐acceptor systems is especially interesting:^30^, ^31^, ^32^, ^33^, ^34^ They enable the realization of thermodynamically reversible assemblies constituted of different functional building blocks in a straightforward manner. Overall, their stability is influenced through the careful choice of the type of non‐covalent interactions, on the one hand, and their fine‐tuning by external factors such as temperature, solvent, etc., on the other.

**Figure 1:**
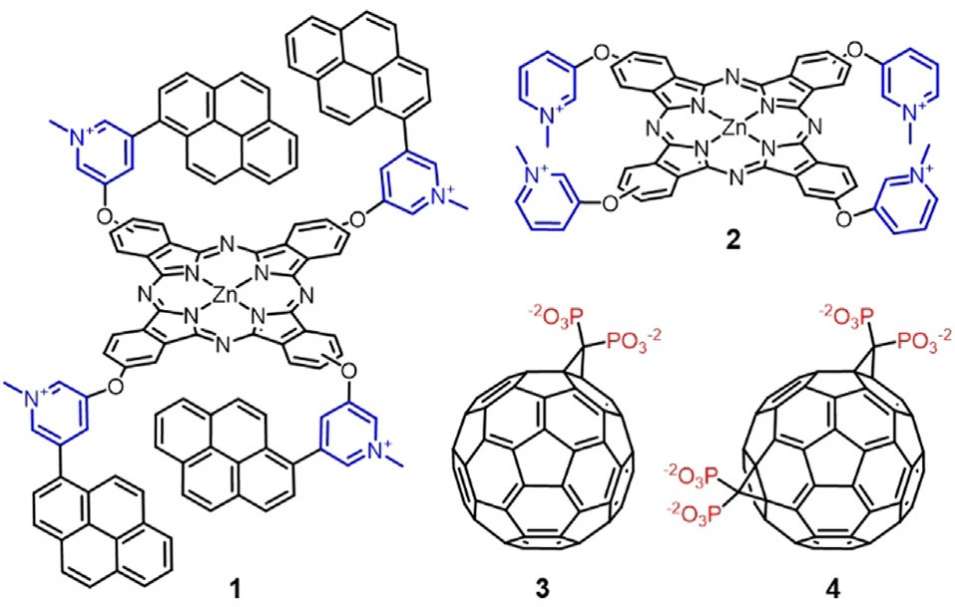
Chemical structures of tetracationic ZnPc **1**, bearing four pyrene moieties, and ZnPc reference **2**. Chemical structures of electron acceptor tetra‐ and octaanionic fullerenes **3** and **4**, respectively. Iodide (ZnPc) and sodium (C_60_) counterions are omitted for clarity purposes.

The grand challenge in the field is to use aqueous media for the design, synthesis and characterization of electron donor‐acceptor systems. A recent example is constituted by single‐walled carbon nanotubes (SWCNTs).^35^, ^36^, ^37^ Solubility of artificial photosynthetic systems in aqueous media is desirable, especially to mimic their natural counterparts more realistically with respect to environmental issues.^37^, ^38^ To this end, a tetracationic ZnPc bearing four peripheral pyrene moieties (**1**) was synthesized and characterized (Figure 1). Synthetic design endows **1** with different modes of aggregation, which can be finely tuned by the amount of organic solvent in the aqueous medium. The aggregation modes range from the monomeric state in DMSO, to well‐known H‐type aggregation in 60:40 water/DMSO and, more interestingly, an unprecedented mode of aggregation in 95:5 water/DMSO. This third mode of aggregation was further characterized by electron microscopy and dynamic light scattering, revealing crystals that form rod‐like structures. Importantly, a symmetry‐breaking charge separation occurs in the aggregates of **1**, as a consequence of the greatly facilitated transfer of electrons and holes between neighboring ZnPc molecules. When these aggregates interact with either tetra‐ or octaanionic water‐soluble fullerenes (**3** or **4**), the resulting unidirectional charge separation yields an appreciably longer‐lived charge‐separated state.

## Results and Discussion

### Synthesis and characterization

ZnPc **1** was prepared as shown in Scheme S1. Briefly, pyrene‐1‐boronic acid pinacol ester was synthesized by dissolving pyrene‐1‐boronic acid in THF, adding it to a pinacol solution in diethyl ether, and refluxing the mixture for 2.5 h.^39^ The resulting pyrene derivative was subsequently reacted with 5‐bromo‐3‐hydroxypyridine via Suzuki cross‐coupling reaction in the presence of Pd(dppf)Cl_2_ as catalyst, leading to the pyrenyl pyridyloxy derivative **5**. Phthalonitrile **6** was obtained by nucleophilic aromatic substitution of 4‐nitrophthalonitrile with **5** in refluxing DMF, and it was easily purified by recrystallization from ethanol. ZnPc **7** was obtained by cyclotetramerization of phthalonitrile **6** in the presence of anhydrous zinc acetate as a metal salt template, and refluxing 2‐dimethylaminoethanol as solvent. ZnPc **1** was obtained by quaternization of **7** with methyl iodide. In order to obtain a suitable reference, ZnPc **2** was synthesized without the pyrenyl moieties (see SI).^40^

The anionic C_60_ derivatives **3** and **4** were prepared by adapting a previously described methodology, based on the Bingel reaction between tetraethyl bromomethylenediphosphonate **8** and pristine C_60_ in different stoichiometric ratios, yielding monoadduct **9** and bisadduct **10** (see SI, Scheme S2).^41^ Bisadduct **10** was obtained as a mixture of regioisomers, which could be purified by column chromatography (with a gradient from chloroform to chloroform/THF 1:1 as eluent) and fully characterized by ^31^P‐NMR, HPLC, and ESI‐MS (Figure S1–S3, respectively). The electron‐accepting features of **9** and the different **10** regioisomers were evaluated by cyclic voltammetry (CV) in an anhydrous *o*‐DCB/1,4‐dioxane (4:1) mixture, employing TBAPF_6_ 0.1 m as supporting electrolyte, Pt electrode as working electrode, Pt wire as counter electrode, and Ag/AgNO_3_ non‐aqueous electrode as pseudo‐reference electrode. The voltammograms showed very similar first reduction potentials in all cases (Figure S4, Table S1), confirming that the amount and distribution of bisphosphonate groups over the C_60_ structure does not affect the electron accepting properties. Therefore, the *trans3* regioisomer was selected for further experiments due to the higher yield obtained in its synthesis. In order to render aqueous solubility, monoadduct **9** and bisadduct **10** were hydrolyzed in presence of trimethylsilyl iodide in CCl_4_, yielding the phosphonic acid derivatives **3** and **4**, respectively.

### Aggregation Studies

**Figure 2:**
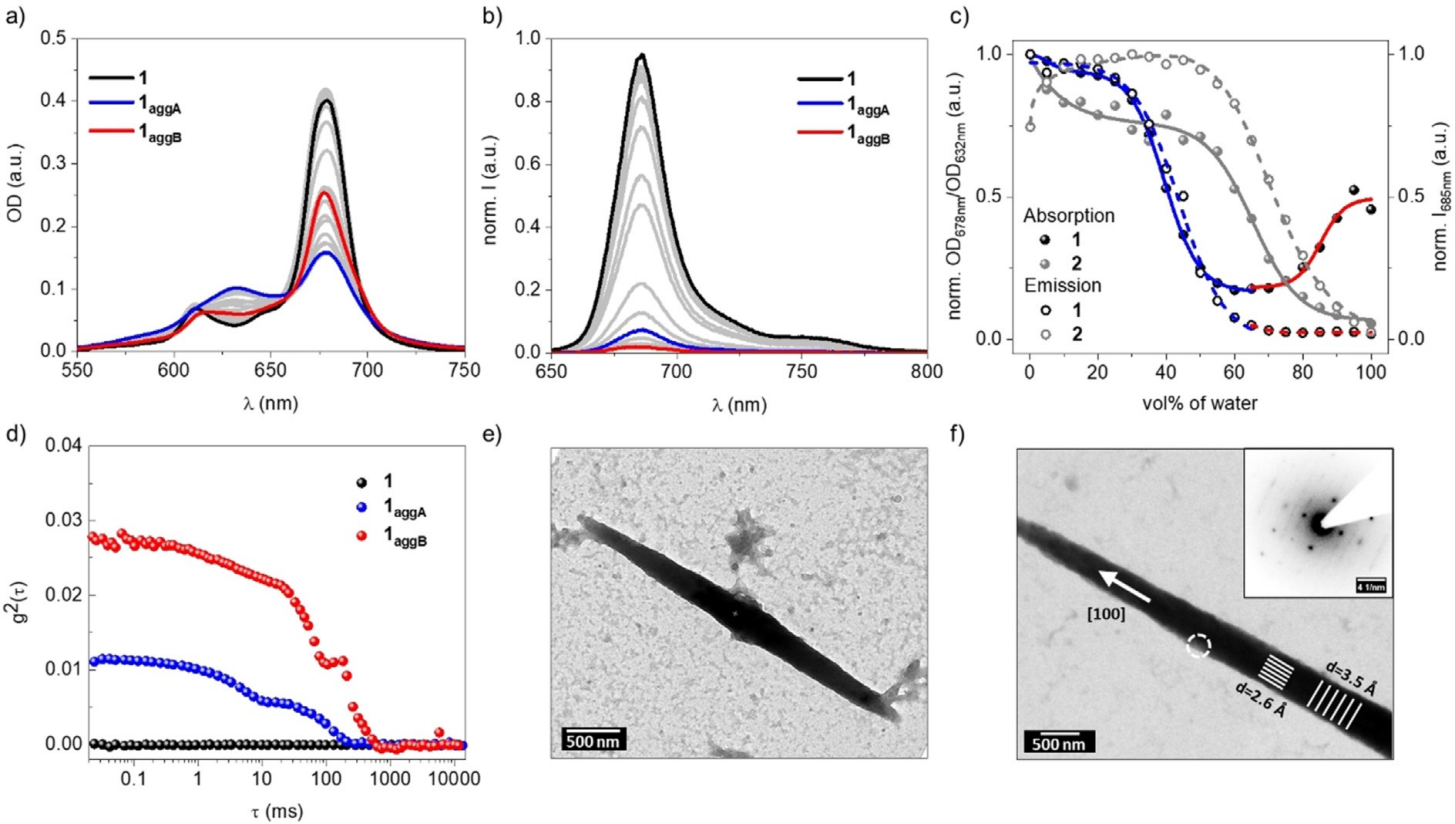
a) Absorption and b) fluorescence spectra (*λ*_exc_=615 nm, inner filter corrected) of **1** (2×10^−6^ m) recorded for different solvent ratios of DMSO and water, from 100 vol% DMSO to 99.8 vol% water. c) Plot of the corresponding normalized Q‐band ratios (678/632 nm) and normalized emission intensity at 685 nm of **1** and **2**. d) Intensity autocorrelation function of **1**, **1_aggA_**, and **1_aggB_** (2.0×10^−5^ m) at a scattering angle of *Θ*=90° and a laser wavelength of 633 nm. e) and f) Bright field transmission electron microscopy (BF‐TEM) images of one crystal formed from **1_aggB_**. Growth axis is indicated as [100]. White circle shows aperture placement for the recording of the electron diffraction pattern shown in the inset. Perpendicular lattice planes are indicated by white lines in the overlay with the spacing as extracted from SAED. Please note the diffraction pattern has been rotated to rotationally align with the bright field image.

Despite carrying four positive charges, **1** shows no solubility in pure water, due to the hydrophobic nature of the ZnPc and the pyrenes in periphery. However, aqueous dispersions can be achieved by injection of water‐miscible organic solutions of **1**, such as in DMSO, showing remarkable stability even below 1 % of the organic solvent. Indeed, the aggregation behavior of **1** depends strongly on the relative water/DMSO ratio, as shown by steady‐state absorption (Figure 2 a) and fluorescence spectroscopies (Figure 2 b). In particular, the absorption spectrum of **1** in DMSO features a sharp Q‐band absorption centered at 678 nm, typical of molecularly dissolved ZnPcs. Increasing the water content up to 60 vol% induces **1** to aggregate (**1_aggA_**), as shown by the broadening of the Q‐band and appearance of a new band at 635 nm (Figure 2 a, blue spectrum). This behavior, typical of H‐type aggregates,^42^ is consistent with the decrease of fluorescence observed in Figure 2 b (blue spectrum). Further increase of the water content to 99.8 vol % results in the diminishment of the 635 nm aggregation band and recovery of the Q‐band narrowness (Figure 2 a, red spectrum). However, the fluorescence remains strongly quenched in this case (Figure 2 b, red spectrum), suggesting a new mode of aggregation (**1_aggB_**). In stark contrast, references **2** and **7**, lacking the pyrene moieties and the positive charges, respectively, give rise to only one form of aggregation, with features similar to **1_aggA_** (Figure S5). A clear trend is observed by plotting the ratio between the absorption at 678 and 632 nm as a function of the vol% of water. In contrast to ZnPc **2** (Figure 2 c, grey solid dots), which shows a single aggregation behavior, **1** (Figure 2 c, black solid dots) exhibits a decrease in ratio below 60 vol% water (**1_aggA_**), followed by an increase at higher water contents (**1_aggB_**). The latter aggregation state is, therefore, consequence of a delicate interplay between the repulsive electrostatic forces between pyridinium moieties and the attractive hydrophobic effects induced by the pyrene units in aqueous media.

The size of the different aggregates of **1** can be obtained from dynamic light scattering (DLS) measurements (Figure 2 d), in which the autocorrelation function of the scattered light illustrates the formation of **1_aggA_** and **1_aggB_** with increasing water content. Importantly, no scattering intensity is detectable for molecularly dissolved **1** in DMSO. The scattering intensity rises by gradually increasing the water content to 60 and 95 vol %.^43^ For 60 vol % water, a clear bimodal decay function is found that shifts to higher decay times for 95 vol % water. The sizes of the different aggregates of **1** are derived from angle‐dependent dynamic light scattering (DLS) measurements. To this end, the hydrodynamic radius R_H_ is obtained by extrapolating the angle‐dependent apparent diffusion coefficients against *θ*=0°. The extrapolated diffusion coefficient can be converted to the hydrodynamic radius (Figure S6). The data fitting for 60 vol% water results in two distinct species, one with a hydrodynamic radius of *R*_H1_=200 nm and a second one in a size range of micrometers (formally *R*_H2_=5 μm). By addition of water, the autocorrelation function shifts towards higher decay times and correspondingly the second species becomes even larger. At the same time, *R*_H1_ splits into two, which might be due to Ostwald ripening of the smaller particles. The intermediate form is likely to grow to the size of the larger particles. The existence of multiple species hinders data interpretation with static light scattering (SLS). Interpreting the structure is challenging in this case. Instead, further insights into the morphology of **1_aggB_** were obtained by transmission electron microscopy (TEM). ZnPc **1** tends to form large needles of micrometer dimensions in aqueous dispersion (Figures 2 e and S17), with size distributions that are in good agreement with the DLS measurements. Selected area electron diffraction (SAED) reveals crystalline stacking along the growth axis (termed [100] in Figure 2 f) with a lattice plane spacing of ca. 3.5 Å, suggesting π—π stacking. The perpendicular lattice plane spacing is ca. 2.6 Å. However, electron beam damage renders an orientation of the crystallites in different zone axes impossible and, in turn, no definite statements regarding the global structural parameters can be made.

In order to show that the spectroscopic features depend on the degree of aggregation, we focused on temperature‐dependent experiments. In the case of **1_aggB_**, the fluorescence spectra of **1** at 5.0×10^−7^ m in 99 % water were recorded at temperatures ranging from 363 to 273 K (Figure S9). At high temperatures, the fluorescence centred at 685 nm is recovered. It decreases and shifts to 705 nm upon lowering the temperature. The ratio between the emission maxima of the aggregates and the monomers, that is, 705 and 685 nm, respectively, is fit to a supramolecular polymerization model (Figure S8),^44^, ^45^, ^46^ yielding an aggregation constant of 8.4×10^3^ m ^−1^. Absorption studies were not taken into account due to the lack of differences in absorption between **1_aggB_** and **1**. In the case of **1_aggA_**, fluorescence assays in a concentration regime of 5.0×10^−6^ m resulted in an aggregation constant of 8.0×10^3^ m ^−1^.

### Theoretical Calculations

**Figure 3:**
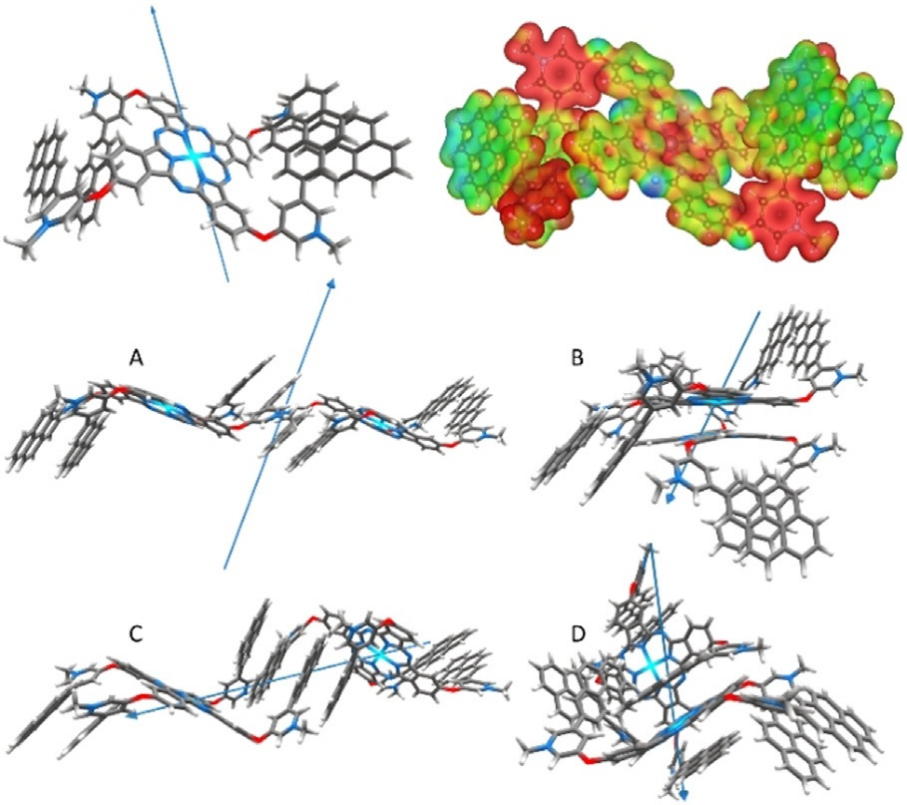
Top: wB97xd/def2‐SVP optimized structure of monomer **1 e** with a total dipole moment of 31.4 Debye as indicated by the blue vector (left). Electrostatic potential (0.15–0.35, blue‐red) mapped on the electron density (isovalue=0.02) of **1 e** (top right). Bottom: wB97xd/def2‐SVP optimized structures of dimers A‐D.

Combined Dreiding force field^47^ and wB97xd/def2‐SVP‐density functional theory (DFT)^48^, ^49^, ^50^ calculations with a polarizable continuum solvent model were used to elucidate possible aggregation alignments of **1** (see SI for all details). The most stable conformations of **1** and **2** in their monomeric forms are achieved by facing the positively charged methylpyridinium groups outwards towards the solvent (Figure S19/20 and Table S2). This orientation also promotes pyrene‐pyrene or pyrene‐ZnPc interactions in case of **1**, resulting in either a planar (**1 e**) or bent ZnPc‐core (**1 c**,**d**), respectively. In DMSO, conformer **1 e** (Figure 3, top) is 37 kcal mol^−1^ more stable than **1 a**,**b** and 3 kcal mol^−1^ more stable than the bended conformation **1 c**. Thus, we expect the initial aggregation to be based on conformer **1 e**, which offers four possible pyrene‐pyrene‐ and two ZnPc‐ZnPc‐interactions. Based on a force‐field prescreening, four dimer arrangements of **1 e** were chosen and optimized with DFT. The dimerization energy (*E*_D_) for ZnPc‐ZnPc‐ (C) or ZnPc‐pyrene‐ (D) interacting dimers are −48 and −55 kcal mol^−1^, respectively, in water. The two pyrene‐pyrene interacting dimers A and B are stabilized by −20 kcal mol^−1^ (Figure S22/23, Table S4–6). The pronounced solvent dependence stems from the high Coulomb repulsion in the dimers, as inferred from a solvent independent *E*_D_ for uncharged dimers of ZnPc **7**. This trend is well in line with the distances between neighboring pyridinium units at 8.2 Å for A and 5.8 Å for C.

Based on the rather large dipole moments of 31.3 Debye for the monomers and their different spatial orientations, we further divided the dimers by means of their total dipole moments. In particular, 482 and 485 Debye are the sums for dimer B and D, respectively, while the sums are 127 and 195 Debye for C and A, respectively. Any further growth goes hand in hand with an increase in dipole moment, especially for B and D; a finding that assists in neglecting these arrangements. In addition, the weaker‐stabilizing pyrene‐pyrene interactions outnumber possible ZnPc‐ZnPc interactions. They are based on less Coulomb repulsions due to the fact that the pyridinium moieties are at larger distances. A coarse linear extrapolation from dimer‐derived interaction energies towards larger aggregates indicates similar stabilization per monomer, especially for A and C (Table S7, Figure S24).

Additional insight into the structural alignment came from the comparison of time‐dependent TD‐DFT predicted vertical excitations and experimental absorption data (Table S8/9, Figure S25–28). The first two predicted vertical transitions (S_0_‐S_1/2_) for the monomer are both about 1.9 eV and in good agreement with the experimental data. In case of the dimers, the two ZnPc‐centered transitions split into four. For the pyrene interacting dimers A and B, none of the four show significant changes in energy. In stark contrast, the ZnPc‐interacting dimers differ substantially in energy. Although the S_0_‐S_1/2_ transitions are stabilized by 0.1 eV for both, a significant oscillator strength is only predicted for dimer D. Further deviation from the monomeric vertical energies are found for dimer C, where S_0_‐S_3/4_ transitions with significant oscillator strength are found at 1.95 and 1.99 eV. Comparison with the hypsochromic shifted absorption essays indicate that **1_aggA_** is likely a direct ZnPc‐ZnPc interaction as proposed by dimer C. In contrast, the lack of absorption changes for **1_aggB_** can be rationalized by the increased ZnPc‐ZnPc distance in this aggregation regime. To evaluate the ZnPc‐distance in the aggregates, the second layer of aggregation needs to be considered to account for the three‐dimensional growth. Figures S29–32 show the force‐field optimized geometries for aggregates based on dimers of A and C. While the latter is built around by ZnPc‐ZnPc columns with alternating, perpendicular pyrene motifs for secondary coordination, aggregates of A feature planes of primarily pyrene‐interactions. In addition, an intermediate structure (MIX) was found.

It is best described as perpendicular arranged strings of A, which are supported by ZnPc‐interactions similar to what is found for C (Figure S33). Here, the better pyrene overlap with respect to C is achieved by decreasing the steric hindrance via increasing the ZnPc‐interplane distances from 3.5 to 5.0 Å (Figure S34/35). As a consequence of reduced ZnPc‐ZnPc interactions, the TD‐DFT predicted vertical excitations closer resemble monomeric behavior (Figure S36). Still, aggregates C and MIX are structurally similar, which is in line with the experimentally observed gradual change in aggregation. This makes MIX a more likely arrangement for **1_aggB_** than A, although we cannot rule out the presence of the latter.

### Complexation Studies

The ground‐ and excited‐state interactions of cationic ZnPcs **1** and **2** with anionic fullerene derivatives **3** and **4** were investigated by absorption and fluorescence spectroscopies. First, monomeric **1** and **2** at constant concentrations of 2.0×10^−6^ m were titrated in pure DMSO with up to three equivalents of **3** and **4**. Throughout these titrations, the Q‐band absorption decreases in intensity and undergoes a 8 nm red shift. In addition, the fluorescence is quantitatively quenched (Figure S11–14). We ascribe these trends to the formation of ZnPc‐C_60_ electron donor‐acceptor hybrids. The overall stoichiometry was determined by applying 1:1 or 2:1 binding model fits to our titration data and evaluating the quality of each fit. 1:1 and 2:1 ratios were found for **1‐3** and **1‐4** species, respectively, consistent with charge neutralization in the resulting hybrid. This points to electrostatic interactions as the main driving force for the binding. Taking this into account, ZnPc complexes with **3** consists of a one‐step process, as shown in Equation (1), while ZnPc complexes with 4 involve two steps for their formation—see Equations (1) and (2):^51^

(1) $$H+G↔\mathrm{HG}\;\;K{}_{1}=\left[\mathrm{HG}\right]/\left(\left[H\right]\left[G\right]\right)$$

(2) $$\mathrm{HG}+H↔H{}_{2}G\;\;K{}_{2}=\left[H{}_{2}G\right]/\left(\left[\mathrm{HG}\right]\left[H\right]\right)$$

Here, H is the ZnPc host, G the fullerene guest, HG the 1:1 complex, and H_2_G the 2:1 complex. K denotes the binding constants for each of the processes described. With Equations (1) and (2) in hand, the binding constants for **1‐3**, **1‐4**, and **2‐3** were determined in absorption and fluorescence titrations (Figure S11–13), using Reactlab Equilibria as fitting software (Table 1).

**Table 1:** Binding constants of different ZnPc and fullerene combinations. Titrations were carried out at constant ZnPc concentrations of 2.0×10^−6^ m in DMSO.

Complex	*K*_abs_ [m ^−1^]	*K*_fluor_ [m ^−1^]
**1‐3**	3.6×10^6^	6.5×10^6^
**1‐4**	*K*_1_=3.6×10^6^ *K*_2_=3.5×10^6^	*K*_1_=7.0×10^6^ *K*_2_=1.9×10^6^
**2‐3**	1.3×10^5^	2.1×10^5^

Overall, the design and incorporation of an electrostatic interaction motif enables high binding constants in DMSO with values of around 10^5^ m ^−1^ (see Table 1 for **2‐3**). Incorporation of pyrene leads to an increase of the binding constant by one order of magnitude (see Table 1 for **1‐3** and **1‐4**). This phenomenon can be explained by the additional interactions between the pyrene surface and C_60_ via π–π interactions. Remarkably, **1‐4** formation shows similar *K*_1_ and *K*_2_ values, which implies that the two processes are independent.

**Figure 4:**
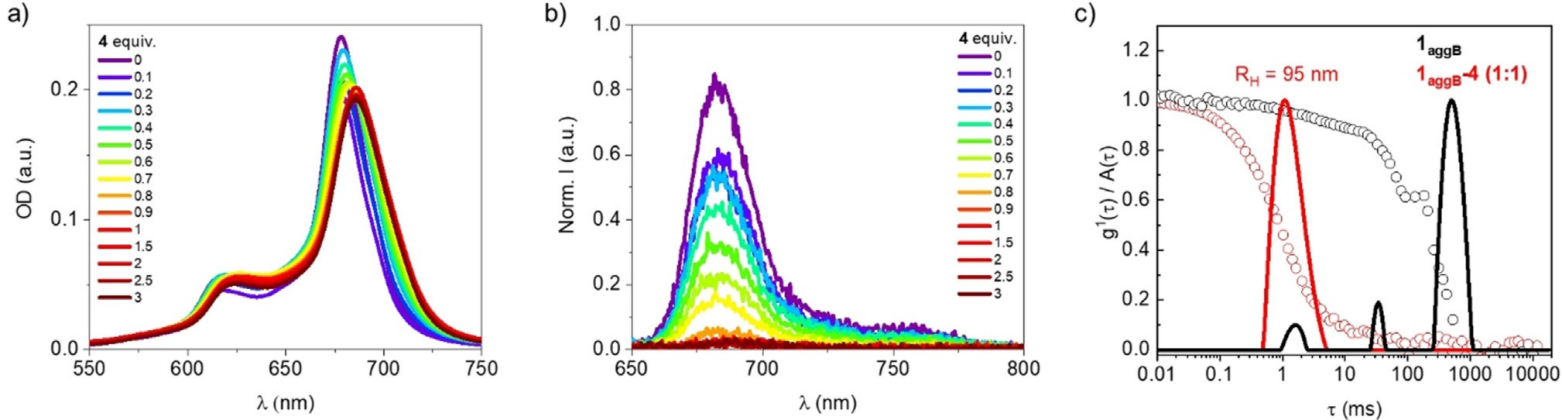
a) Absorption and b) fluorescence spectra (*λ*_exc_=615 nm, inner filter corrected) of **1_aggB_** at a constant concentration of 2.0×10^−6^ m in 95 vol % water, titrated with up to three equivalents of **4**. Absorption and fluorescence spectra are corrected by subtraction of the absorption and fluorescence of pure **4**, respectively. c) Dynamic light scattering experiments of **1_aggB_** and **1_aggB_‐4** (1:1) at 2.0×10^−5^ m in 95 vol % water (at a scattering angle of *Θ*=90°, laser wavelength 633 nm).

The interaction between the anionic fullerenes and **1** in the aggregated state was also studied. No significant data were obtained from titrations of **1_agg_** with **3**, due to the poor solubility of the latter in water. **1_aggA_** and **1_aggB_** (2.0×10^−6^ m), in turn, were titrated with up to three equivalents of **4** in 60 vol % and 95 vol % water, respectively. From these experiments, even if the data cannot be fitted to any theoretical model due to the intrinsic uncertainty of the stoichiometry of the aggregates, qualitative information can be obtained. For instance, both **1_aggA_** and **1_aggB_** show a red shift of ca. 8 nm of both the aggregation and the Q‐band in the absorption spectra (Figure 4 a and S15). However, no other significant changes in the overall shape of the absorption spectrum are observed, despite the higher binding affinity of, for instance, **1**–**4** compared to **1_aggB_**: 10^6^ m ^−1^ vs. 10^3^ m ^−1^. Similarly, the fluorescence spectral features are preserved upon addition of **4**, while the emission intensity is quantitatively quenched upon exceeding 1:1 ratio (Figure 4 b and S15). On these bases, we hypothesize that, in aqueous media, **1** remains as aggregate, while **4** binds to the periphery of **1_agg_** through electrostatic and π–π interactions.

In order to determine the nature of the colloidal hybrid, temperature‐dependant absorption experiments were performed at a ZnPc concentration of 2.0×10^−5^ M. Temperature increases of a **1_aggA_** solution in the presence of 0.5 equivalents of **4** trigger a decrease of the aggregation band at 632 nm and an increase of the Q‐band absorption at 678 nm (Figure S16). A subsequent decrease of the temperature results in the recovery of the initial features, suggesting a better stabilization for the H‐type aggregation relative to the **1‐4** hybrid formation. For **1_aggB_**, no deaggregation is observed. Instead, an irreversible trend suggests another thermodynamically more stable form that is only accessible upon heating (Figure S10). However, in the presence of **4** at a 2:1 ratio, the **1_aggB_** stability is increased to the point that no significant absorption changes evolve within the studied temperature and concentration ranges (Figure S16).

Further insights regarding the **1_aggB_‐4** morphology were obtained by TEM images. The rod‐like shape of the **1_aggB_** aggregates are still observable (Figure S18).^52^ A more statistical overview stems from DLS. With increasing concentration of **4**, the previously multimodal decay function changes to a monomodal decay (Figure 4 c). An R_H_=95 nm was inferred from such data, leading to the conclusion that the presence of **4** affects the size of the crystals with its anionic charges and by increasing stability in solution, compared to the crystals in the absence of **4**. The radius of gyration R_G_ can be calculated from the angle‐dependent scattering intensity, while the shape of the particles can be estimated from the ratio of R_G_ to R_H_. With an R_G_/R_H_=1.68, a prolate ellipsoid is expected as in pristine **1_aggB_** (Figure S7).^53^

Interactions of the **1_aggB_** aggregates with **3** and **4** were also theoretically modeled with a forcefield approach and are illustrated in Figures S37–39. While for the A dimer arrangement (see above) the interaction is limited to the periphery, the C and MIX arrangements offer vacancies for interactions and direct intercalations of **3** and **4**. In case of monomeric **1**, 1:1 and 2:1 complexation were investigated with force‐field annealing, followed by further optimization with DFT. In all cases, direct C_60_‐ZnPc interactions are accomplished by the pyrenes wrapping around C_60_, and, thus, increasing the van der Waals contact area.

### Photophysical Studies

Prior to transient absorption measurements, spectroelectrochemical experiments served to determine the spectroscopic fingerprints of the one‐electron oxidized and reduced forms of **1** and **2** in DMSO. At a voltage of +1.2 V vs. Ag‐wire, features of the one‐electron oxidized form are discernible (Figure S40 and S41, respectively). Besides the ground‐state bleaching (GSB) of the Soret‐ and Q‐band absorptions, new features arise at 422, 520, and 840 nm. These bands fit the values found in the literature for the one‐electron oxidized form of ZnPcs perfectly.^54^ At a voltage of −0.2 V vs. Ag‐wire, features of the one‐electron reduced forms of **1** and **2** are discernable (Figure S40 and S41, respectively). In particular, new features at 560–580, 820, and 960–980 nm arise, accompanied by GSB, which are also found in the literature upon reduction of ZnPcs.^54^ Comparing the GSB and evolving features during the first oxidation and reduction of **1** with **2**, we exclude any participation of the pyrenes in these redox processes.

The role of the aggregation mode in the excited state was studied by deconvolution of the deactivation mechanism after photoexcitation of **1_aggA_** and **1_aggB_**, by pump‐probe experiments. Solutions of **1** at 2.0×10^−5^ m were excited at 630 nm, and time delays ranging from femto‐ to microseconds were recorded. The selected excitation wavelength excites the ZnPcs in the Q‐band/aggregation‐band absorption exclusively. This avoids the excitation of pyrenes and C_60_.

**Figure 5:**
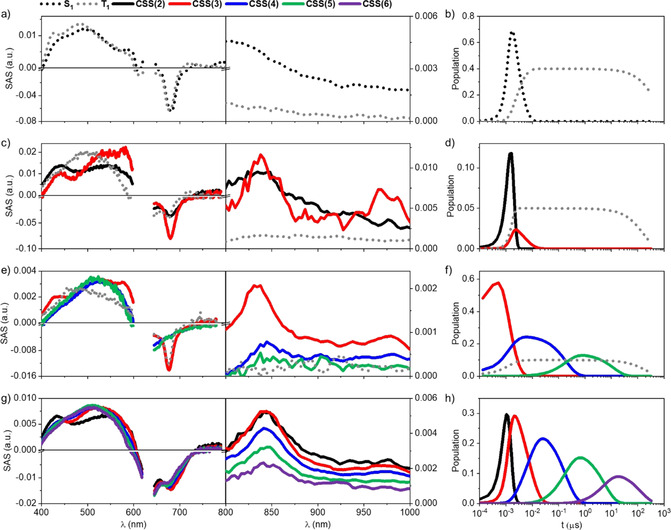
Species associated differential absorption spectra (SAS) (left and center) and population over time (right) obtained upon deconvolution of spectra with Target Analysis (GloTarAn) of **1** (a and b), **1_aggA_** (c and d), **1_aggB_** (e and f), and **1_aggB_‐4** (g and h, 1:2 ratio). Raw spectra were received upon nanosecond pump‐probe experiments (*λ*_ex_=630 nm, *c*=2.0×10^−5^ m) at time delays from 0–250 μs.

Initially, reference measurements were performed in pure DMSO, to examine the excited state deactivation of monomeric **1** (Figure 5 a and S42–44) and **2** (Figure S45/46). Directly after photoexcitation, the singlet‐excited state characteristics of both ZnPcs exhibit maxima at 455, 515, and 820 nm together with the GSB, with lifetimes of 1.68 and 2.24 ns, respectively.^55^ Next, the triplet‐excited state with features at 460 and 495 nm evolves from the singlet‐excited state with a yield of 45 % and a lifetime of 498 μs for **1**. Full recovery of the GSB due to the population of the ground state is the last step in the kinetic model (Figure S44). For **2** in DMSO, the same triplet‐excited state is found, but it decays slowly to the ground state.

With this kinetic model in hand, we turned our attention to the changes in terms of spectroscopy and kinetics upon addition of **4**. Due to the prominent aggregation in DMSO of the complexes involving **2** (even leading to precipitation when is confronted with **3**),^56^ these studies were focused on the complex **1‐4**, for which significant changes relative to **1** are observable (Figures S47 and 48). For example, the singlet‐excited state of **1** is discernible after photoexcitation, but it is short‐lived with 1.7 ps. The back‐to‐back formation of two transient species with lifetimes of 18 and 638 ps, which feature two sets of spectroscopic signatures, commences with the singlet‐excited state decay. It is, on the one hand, those of the one‐electron oxidized form of **1** at 445, 520 and 841 nm, which we observed in our spectroelectrochemical measurements (vide supra). On the other hand, we note those of the one‐electron reduced form of **4** at 1000 nm, which are in sound agreement with the literature.^57^

The first of the two species, however, gives rise to slightly broadened signatures and is assigned to a **1^δ+^‐4^δ−^** charge‐transfer state. This **1^δ+^‐4^δ−^** charge‐transfer state then undergoes full charge separation, which affords the **1^.+^‐4^.−^** charge‐separated state as the second of the two species. Only 25 % of the **1^.+^‐4^.−^** charge‐separated state deactivates to the triplet excited state of **1**. The later reinstates the ground state with a lifetime of 734 μs. A detailed mechanistic model is shown in Figure S49.

Upon transitioning from **1** to **1_aggA_**, significant changes in transient features and lifetimes are observable (Figure 5 c and S50). It is highly relevant that the singlet‐excited state lifetime of **1_aggA_** is quenched by three orders of magnitude down to 3.5 ps. As the singlet‐excited state decay comes to an end, new features develop at 445, 555, 834, and 960 nm. These features are exact matches to the spectroscopic fingerprints recorded upon reduction and oxidation of **1** (vide supra). In other words, excitation of the H‐type **1_aggA_** triggers a symmetry‐breaking charge separation and consequently yields a **1_aggA_^.−^‐1_aggA_^.+^** charge‐separated state. Three lifetimes of 52, 176, and 3600 ps are derived for the **1_aggA_^.−^‐1_aggA_^.+^** charge‐separated state. We infer a transfer of electrons and/or holes between neighboring **1** to assist in a spatial separation of charges and, consequently, a slowing‐down of the charge recombination. Each of these transfer events competes, however, with the intrinsic charge recombination. Thus, the relative amplitudes decrease significantly and amounts to, for example, only 5 % in the case of the 3600 ps‐lived **1_aggA_^.−^‐1_aggA_^.+^** charge‐separated state. For **1_aggB_**, the correspondingly formed **1_aggB_^.−^‐1_aggB_^.+^** charge‐separated state exhibits five lifetimes; next to the three lifetimes discussed for the **1_aggA_^.−^‐1_aggA_^.+^** charge‐separated states, two additional lifetimes of 190 ns and 12 μs (Figure 5 e and S52). A greatly facilitated transfer between neighboring **1_aggB_** enables not only a remarkable lifetime of 12 μs but also a 5 % overall yield.^58^ Details regarding the kinetic models are shown in Figure S51/53.^59^

At first glance, the transient absorption measurements with **1_aggB_‐4** (Figures 5 g and S54) are identical to what was discussed for **1_aggB_**.^60^ We reach the same conclusions from both the spectroscopic and the kinetic evaluations. However, a closer look at the data fittings revealed a notable difference: Best fits of the experimental data necessitate the inclusion of another species in the kinetic model: 132 μs is the lifetime of the sixth charge‐separated state. Importantly, the 840 nm signature of the one‐electron oxidized form of **1_aggB_** is persistent over the entire timescale.^61^ Therefore, we conclude that the longest lifetime of 132 μs stems from a charge transfer that evolves from **1_aggB_** to **4**, which is located at the periphery of the aggregates. Experiments with various equivalents of **4** (Figures S54–59) suggest an increase of the **1_aggB_^.+^‐4^.−^** charge‐separated state up to a yield of 7 % for a 1:2 ratio. Furthermore, the formation of the triplet‐excited state is fully inhibited in the presence of **4**, corroborating the formation of the **1_aggB_^.+^‐4^.−^** charge‐separated state, which is lower in energy than the **1_aggB_^.−^‐1_aggB_^.+^** charge‐separated state.

## Conclusion

In this paper, we have described a tetracationic ZnPc featuring pyrenes, which shows an unprecedented mode of aggregation in aqueous medium. The resulting linear supramolecular polymers bundle into crystalline rod‐like structures. Computational calculations suggest that the formation of this highly organized supramolecular architecture relies on the water‐promoted conformational reorganization of the pyrene‐containing substituents. This is responsible for the fruitful interplay between light harvesting and charge‐separation in **1**. Importantly, when the system interacts with a tetra‐ or octaanionic water‐soluble fullerene under the same conditions, charges are funneled in the hybrid aggregates to the strongest electron‐accepting units, that is, the fullerenes, resulting in longer‐lived charge‐separated states. In this case, both the self‐assembly of **1** and the complexation with fullerene **3** or **4** are controlled by a subtle interplay between electrostatic and π–π interactions, favored by the aqueous medium. Hence, our work represents a versatile approach, which enables water to modulate the synergy between different non‐covalent interactions and, in turn, to achieve multi‐chromophore supramolecular assemblies. In the latter, all the fundamental processes of natural photosynthesis are operative and give rise to highly efficient artificial photosynthetic systems.

## Conflict of interest

The authors declare no conflict of interest.

## Supporting information

As a service to our authors and readers, this journal provides supporting information supplied by the authors. Such materials are peer reviewed and may be re‐organized for online delivery, but are not copy‐edited or typeset. Technical support issues arising from supporting information (other than missing files) should be addressed to the authors.

Supplementary

## References

[1] R. E. Blankenship , Molecular Mechanisms of Photosynthesis, Wiley-Blackwell, Chichester, 2014.

[2] D. Gust , T. A. Moore , Science 1989, 244, 35–41. 17818844 10.1126/science.244.4900.35

[3] J. Barber , Chem. Soc. Rev. 2009, 38, 185–196. 19088973 10.1039/b802262n

[4] P. D. Frischmann , K. Mahata , F. Würthner , Chem. Soc. Rev. 2013, 42, 1847–1870. 22850767 10.1039/c2cs35223k

[5] S. Berardi , S. Drouet , L. Francàs , C. Gimbert-Suriñach , M. Guttentag , C. Richmond , T. Stoll , A. Llobet , Chem. Soc. Rev. 2014, 43, 7501–7519. 24473472 10.1039/c3cs60405e

[6] M. Rudolf , S. V. Kirner , D. M. Guldi , Chem. Soc. Rev. 2016, 45, 612–630. 26744992 10.1039/c5cs00774g

[7] D. K. Dogutan , D. G. Nocera , Acc. Chem. Res. 2019, 52, 3143–3148. 31593438 10.1021/acs.accounts.9b00380

[8] J. Yang , M.-C. Yoon , H. Yoo , P. Kim , D. Kim , Chem. Soc. Rev. 2012, 41, 4808–4826. 22659941 10.1039/c2cs35022j

[9] P. Parkinson , C. E. I. Knappke , N. Kamonsutthipaijit , K. Sirithip , J. D. Matichak , H. L. Anderson , L. M. Herz , J. Am. Chem. Soc. 2014, 136, 8217–8220. 24878362 10.1021/ja504730j PMC4073835

[10] P. Parkinson , N. Kamonsutthipaijit , H. L. Anderson , L. M. Herz , ACS Nano 2016, 10, 5933–5940. 27176553 10.1021/acsnano.6b01265 PMC4928140

[11] J. J. Li , Y. Chen , J. Yu , N. Cheng , Y. Liu , Adv. Mater. 2017, 29, 1701905. 10.1002/adma.201701905 28585340

[12] Y. M. Liu , H. Hou , Y. Z. Zhou , X. J. Zhao , C. Tang , Y. Z. Tan , K. Müllen , Nat. Commun. 2018, 9, 1901. 29765041 10.1038/s41467-018-04321-6 PMC5954131

[13] R. E. Blankenship , D. M. Tiede , J. Barber , G. W. Brudvig , G. Fleming , M. Ghirardi , M. R. Gunner , W. Junge , D. M. Kramer , A. Melis , et al., Science 2011, 332, 805–810. 21566184 10.1126/science.1200165

[14] B. K. Rugg , M. D. Krzyaniak , B. T. Phelan , M. A. Ratner , R. M. Young , M. R. Wasielewski , Nat. Chem. 2019, 11, 981–986. 31548665 10.1038/s41557-019-0332-8

[15] S. Fukuzumi , K. Ohkubo , T. Suenobu , Acc. Chem. Res. 2014, 47, 1455–1464. 24793793 10.1021/ar400200u

[16] C. G. Bezzu , M. Helliwell , J. E. Warren , D. R. Allan , N. B. McKeown , Science 2010, 327, 1627–1630. 20339069 10.1126/science.1184228

[17] J. Mack , N. Kobayashi , Chem. Rev. 2011, 111, 281–321. 21175133 10.1021/cr9003049

[18] M. Urbani , G. De La Torre , M. K. Nazeeruddin , T. Torres , Chem. Soc. Rev. 2019, 48, 2738–2766. 31033978 10.1039/c9cs00059c

[19] V. V. Roznyatovskiy , C.-H. Lee , J. L. Sessler , Chem. Soc. Rev. 2013, 42, 306–312. 10.1039/c2cs35418g 23232711

[20] K. M. Kadish , K. M. Smith , R. Guilard , Handbook of Porphyrin Science, World Scientific Publishing Company, London, 2016.

[21] M. Wolf , C. Villegas , O. Trukhina , J. L. Delgado , T. Torres , N. Martín , T. Clark , D. M. Guldi , J. Am. Chem. Soc. 2017, 139, 17474–17483. 29028170 10.1021/jacs.7b08670

[22] G. Bottari , G. De La Torre , T. Torres , Acc. Chem. Res. 2015, 48, 900–910. 25837299 10.1021/ar5004384

[23] O. Trukhina , M. Rudolf , G. Bottari , T. Akasaka , L. Echegoyen , T. Torres , D. M. Guldi , J. Am. Chem. Soc. 2015, 137, 12914–12922. 26401549 10.1021/jacs.5b06454

[24] C. B. Kc , G. N. Lim , F. D'Souza , Angew. Chem. Int. Ed. 2015, 54, 5088–5092; 10.1002/anie.201500156 25726834

[25] S. V. Kirner , C. Henkel , D. M. Guldi , J. D. Megiatto, Jr. , D. I. Schuster , Chem. Sci. 2015, 6, 7293–7304. 28757988 10.1039/c5sc02895g PMC5512142

[26] L. M. Arellano , L. Martín-Gomis , H. B. Gobeze , D. Molina , C. Hermosa , M. J. Gómez-Escalonilla , J. L. G. Fierro , Á. Sastre-Santos , F. D'Souza , F. Langa , Nanoscale 2018, 10, 5205–5213. 29493701 10.1039/c8nr00262b

[27] A. Hirsch , M. Brettreich , Fullerenes: Chemistry and Reactions, Wiley-VCH, Weinheim, 2005.

[28] Chemistry of Nanocarbons (Eds.: T. Akasaka, F. Wudl, S. Nagase), Wiley, Chichester, 2010.

[29] Fullerenes: Principles and Applications (Eds.: F. Langa De La Puente, J.-F. Nierengarten), Royal Society Of Chemistry, Cambridge, 2011.

[30] M. Lehmann , M. Dechant , M. Holzapfel , A. Schmiedel , C. Lambert , Angew. Chem. Int. Ed. 2019, 58, 3610–3615; 10.1002/anie.201812465 30615820

[31] A. De La Escosura , M. V. Martínez-Díaz , D. M. Guldi , T. Torres , J. Am. Chem. Soc. 2006, 128, 4112–4118. 16551120 10.1021/ja058123c

[32] N. Martin , J.-F. Nierengarten , Supramolecular Chemistry of Fullerenes and Carbon Nanotubes, Wiley-VCH, Weinheim, 2012.

[33] F. D'Souza , O. Ito , Chem. Soc. Rev. 2012, 41, 86–96. 21975532 10.1039/c1cs15201g

[34] M. Lederer , U. Hahn , J.-M. Strub , S. Cianférani , A. Van Dorsselaer , J.-F. Nierengarten , T. Torres , D. M. Guldi , Chem. Eur. J. 2016, 22, 2051–2059. 26744015 10.1002/chem.201503315

[35] U. Hahn , S. Engmann , C. Oelsner , C. Ehli , D. M. Guldi , T. Torres , J. Am. Chem. Soc. 2010, 132, 6392–6401. 20402472 10.1021/ja100065h

[36] C. Romero-Nieto , R. García , M. Á. Herranz , C. Ehli , M. Ruppert , A. Hirsch , D. M. Guldi , N. Martín , J. Am. Chem. Soc. 2012, 134, 9183–9192. 22574613 10.1021/ja211362z

[37] E. Anaya-Plaza , M. M. Oliva , A. Kunzmann , C. Romero-Nieto , R. D. Costa , A. de la Escosura , D. M. Guldi , T. Torres , Adv. Funct. Mater. 2015, 25, 7418–7427.

[38] E. Anaya-Plaza , A. Aljarilla , G. Beaune , Nonappa , J. V. I. Timonen , A. de la Escosura , T. Torres , M. A. Kostiainen , Adv. Mater. 2019, 31, 1–6. 10.1002/adma.201902582 31392780

[39] M. Beinhoff , W. Weigel , M. Jurczok , W. Rettig , C. Modrakowski , I. Brüdgam , H. Hartl , A. D. Schlüter , Eur. J. Org. Chem. 2001, 3819–3829.

[40] T. T. Tasso , T. Furuyama , N. Kobayashi , Inorg. Chem. 2013, 52, 9206–9215. 23914935 10.1021/ic4002048

[41] A. L. Mirakyan , L. J. Wilson , J. Chem. Soc. Perkin Trans. 2 2002, 1173–1176.

[42] T. Nyokong , Coord. Chem. Rev. 2007, 251, 1707–1722.

[43] The scattering intensity is only slightly affected due to the generally low absorption (in this area).

[44] T. F. A. De Greef , M. M. J. Smulders , M. Wolffs , A. P. H. J. Schenning , R. P. Sijbesma , E. W. Meijer , Chem. Rev. 2009, 109, 5687–5754. 19769364 10.1021/cr900181u

[45] M. M. J. Smulders , M. M. L. Nieuwenhuizen , T. F. A. De Greef , P. Van Der Schoot , A. P. H. J. Schenning , E. â. W. Meijer , Chem. Eur. J. 2010, 16, 362–367. 19921721 10.1002/chem.200902415

[46] M. J. Mayoral , C. Rest , J. Schellheimer , V. Stepanenko , G. Fernández , Chem. Eur. J. 2012, 18, 15607–15611. 23132726 10.1002/chem.201202367

[47] S. L. Mayo , B. D. Olafson , W. A. Goddard , J. Phys. Chem. 1990, 94, 8897–8909.

[48] J. Da Chai , M. Head-Gordon , Phys. Chem. Chem. Phys. 2008, 10, 6615–6620. 18989472 10.1039/b810189b

[49] F. Weigend , R. Ahlrichs , Phys. Chem. Chem. Phys. 2005, 7, 3297–3305. 16240044 10.1039/b508541a

[50] F. Weigend , Phys. Chem. Chem. Phys. 2006, 8, 1057–1065. 16633586 10.1039/b515623h

[51] P. Thordarson , Chem. Soc. Rev. 2011, 40, 1305–1323. 21125111 10.1039/c0cs00062k

[52] The image quality suffers, however, because of the increased amount of organic material on the copper grids.

[53] A. K. Brewer , A. M. Striegel , Analyst 2011, 136, 515–519. 21109889 10.1039/c0an00738b

[54] M. Lederer , M. Ince , M. V. Martinez-Diaz , T. Torres , D. M. Guldi , ChemPlusChem 2016, 81, 941–946. 31968794 10.1002/cplu.201600197

[55] Besides excitation of the singlet exited state, 15 % of the irradiation leads to more undefined transients with lifetimes of roughly 10 and 200 ps, which are attributed to dimers and smaller aggregates.

[56] Compound **2** aggregates in the presence of **4** in DMSO. This renders an in-depth analysis meaningless. Furthermore, addition of **3** at the concentration and time range of pump-probe experiments lead to instant precipitation, due to inferior solubility in water compared to **4**.

[57] S. González , N. Martín , D. M. Guldi , J. Org. Chem. 2003, 68, 779–791. 12558399 10.1021/jo020412l

[58] The triplet quantum yields are less than 10 % in **1_aggA_** and **1_aggB_**.

[59] Using any other kinetic model for the data led to meaningless results.

[60] The observed changes for the transition from **1_aggA_** to **1_aggA_-4** are the same as for **1_aggB_** to **1_aggB_-4**, and so are not further discussed here, since **1_aggA_-4** is inferior in terms of lifetimes and overall efficiency.

[61] We have, however, no clear cut spectroscopic proof for the one-electron reduced form of **4**.

