# Supplementary material for: Synergy of Electrostatic and π–π Interactions in the Realization of Nanoscale Artificial Photosynthetic Model Systems
Source: Angew Chem Int Ed Engl. 2020 Sep 2;59(42):18786–94. doi: 10.1002/anie.202006014 (PMC7590087; doi:10.1002/anie.202006014)
Supplement: Supplementary file 1 — Supplementary [file ANIE-59-18786-s001.pdf]

Angewandte Chemie
Eine Zeitschrift der Gesellschaft Deutscher Chemiker

Supporting Information

Synergy of Electrostatic and π–π Interactions in the Realization of Nanoscale Artificial Photosynthetic Model Systems

Eduardo Anaya-Plaza[+], Jan Joseph[+], Stefan Bauroth, Maximilian Wagner, Christian Dolle, Michael Sekita, Franziska Gröhn, Erdmann Spiecker, Timothy Clark, Andrés de la Escosura, Dirk M. Guldi,* and Tomás Torres**

anie_202006014_sm_miscellaneous_information.pdf

Table of Contents

Contents

Table of Contents S2
Experimental Procedures S3
General methods S3
Synthesis and characterization of derivatives 1-4 ......S5
Synthesis of **1** S5
Synthesis of **2** S6
Characterization of the ZnPc **1** and its precursors S7
Synthesis and characterization of the anionic fullerene derivatives **3** and **4**. S15
Results and Discussion S20
Aggregation studies of the ZnPcs S20
Organic solvent effect S20
Light scattering S21
Temperature effect S22
ZnPc-C_{60} titrations S24
Monomeric state S24
Aggregated state S26
Transmission electron microscopy S27
Molecular Modelling S28
Spectroelectrochemical characterisation S53
Time resolved transient absorption spectroscopy S54
Measurements in DMSO S54
Measurements in regime A S59
Measurements in regime B S60
References S64
Author Contributions S64

Experimental Procedures

General methods

Chemicals. Chemicals (reagent grade) and solvents (anhydrous, deuterated and HPLC grade) were purchased from Sigma-Aldrich, Alfa Aesar and Scharlau, and used as received without further purification.
Chromatography. Column chromatography was carried out using silica gel Merck-60 (230-400 mesh, 60 Å) as the solid support. Thin layer chromatography (TLC) analyses were performed on aluminum sheets precoated with silica gel 60 F254 (Merck). High-performance liquid chromatography (HPLC) was performed in an Agilent 1100 LC (Agilent Technologies), using ø 4.6 × 250 mm Buckyprep column (Cosmosil) with a linear gradient from t_0 = toluene to t_{40} = toluene/THF (85:15) as eluent; a 1 mL/min flow rate and a detection wavelength of 390 nm were employed.
NMR. NMR spectra were measured on a Bruker AM-300 (300 MHz) or AM-500 (500 MHz) instruments, locked on deuterated solvents. Carbon chemical shifts are measured in ppm relative to trimethylsilane (TMS), using the resonance of the deuterated solvent for internal calibration. Phosphorous chemical shifts are measured in ppm, relative to H_3PO_4 added in a sealed capillary to the sample. The assignment of the NMR signals was supported, in some indicated cases, by 2D-NMR spectra such as COSY, HSQC and HMBC. H_{Ar} denotes aromatic protons, impossible to assign, corresponding to pyrene moieties.
Mass spectrometry. Matrix-assisted laser desorption ionization time-of-flight (MALDI-TOF) experiments were carried out using Bruker Ultraflex III mass spectrometer, in positive ion mode. The convenient matrix for these measurements is indicated for each compound.
Spectroscopy. UV/Vis absorption measurements at room temperature were performed in a 10x10 mm cuvette with a Lambda 2 double beam instrument (Perkin-Elmer), or a JASCO V-660 spectrophotometer. Temperature dependent UV/Vis absorption measurements were performed in a 10x10 mm cuvette with a Cary 5000 double beam spectrometer (Varian). Fluorescence measurements were performed in a 10x10 mm cuvette with a Fluoromax 3 spectrometer (HORIBA Yobin).
Time-resolved absorption measurements were performed by using a Clark MXR CPA 2101 and CPA 2110 Ti:sapphire amplifier (775 nm, 1 kHz, 150 fs pulse width) as the laser source. Femtosecond transient absorption spectra were obtained by using an Ultrafast System HELIOS spectrometer with 150 fs resolution and time delays between 0 to 5500 ps. By focusing a fraction of the fundamental 775 nm onto a 2 mm sapphire disc the probe-visible white light (~400-770 nm) was generated. The probe-(near)IR light (780-1300 nm) was generated using a 1 cm sapphire disc. The excitation wavelength of 630 nm was generated using a non-collinear optical parametric amplifier (NOPA, Clark MXR). A long-pass filter was used to exclude the fundamental 775 nm. Nanosecond transient absorption spectra were obtained by using an Ultrafast System EOS spectrometer with ~1 ns resolution and time delays between 0 to 400 μs. A built-in photonic crystal fiber supercontinuum laser source with a fundamental of 1064 nm at 2 kHz output frequency and pulse width of ~1 ns was used to generate the white light (~370 nm to >1600 nm). All measurements were performed in a 2 mm quartz cuvette under argon atmosphere at room temperature.
Dynamic light scattering. A light scattering setup consisting of a 22 mW laser with a wavelength of λ = 632.8 nm, a CGS 3 goniometer (ALV Langen, Germany) and an ALV 5000 correlator with 320 channels was used. Scattering angles from $30° \leq \theta \leq 150°$ were covered in 10° steps. Data analysis was performed by transformation of the autocorrelation function of the scattered light into the autocorrelation function of the electric field using Siegert equation. The inverse Laplace transformation was carried out to create the distribution of relaxation times using the program CONTIN. The characteristic τ were transferred into the apparent diffusion coefficients using the equation. The apparent diffusion coefficients were extrapolated against $q \rightarrow 0$. The extrapolated diffusion coefficient translates to the hydrodynamic radius with the Stokes Einstein equation. UV/vis measurements were carried out with a Shimadzu UV-spectrometer (UV-1800). Quartz cuvettes with a pathlength of 1 cm purchased from Hellma/Müllheim were used.
Electrochemistry. Cyclic voltammetry (CV) and square wave voltammetry (SWV) studies of C_{60} species were performed with an Autolab PGSTAT30 potentiostate/galvanostate. Measurements were carried out in a home-built one-compartment cell using a three-electrode configuration, o-DCB as a solvent and 0.05 M tetra-n-butylammonium hexafluorophosphate (n-Bu_4NPF_6) as supporting electrolyte. A platinum electrode was used as the working electrode, and a platinum wire as the counter electrode. All potentials were recorded against a Ag/AgNO3 non-aqueous electrode and corrected against the Fc+/Fc redox couple. CV was measured at scan rates of 100 mV s−1. SWV was measured at scan rates of 100 mV s^{-1}. Prior to each voltammetric measurement, the cell was degassed by bubbling with argon for about 20 min.
For spectroelectrochemical measurements a Metrohm PGSTAT101 potentiostat was used in combination with an Avantes AvaLight DH-S-BAL / AvaSpec-2048 fiber spectroscope. To allow light passing through the sample solution, the used working electrode was a close meshed platinum net. As reference electrode served a silver wire, whereas the counter electrode was a platinum wire.

SUPPORTING INFORMATION

Transmission electron microscopy. Transmission electron microscopy was performed with a LEO EM912-Omega (Zeiss) with an acceleration voltage of 80 kV. Selected area electron diffraction (SAED) patterns have been recorded using a Phillips CM30 S/TEM at an accelerating voltage of 300 kV at a camera length of nominally 175 mm with a calibration using gold nanoparticles with known lattice constant. For SAED an aperture with diameter of 300 nm was imposed on the area of interest. Samples were prepared by drop-casting solution onto a carbon coated copper grid. The grids were dried for 1h in a desiccator.

Sample preparation. Stock solutions for all ZnPcs were prepared in DMSO. Mixed water/DMSO solutions were obtained by injecting the corresponding amounts of stock solution into milliQ ultrapure water.

Synthesis and characterization of derivatives 1-4

*Synthesis of **1***

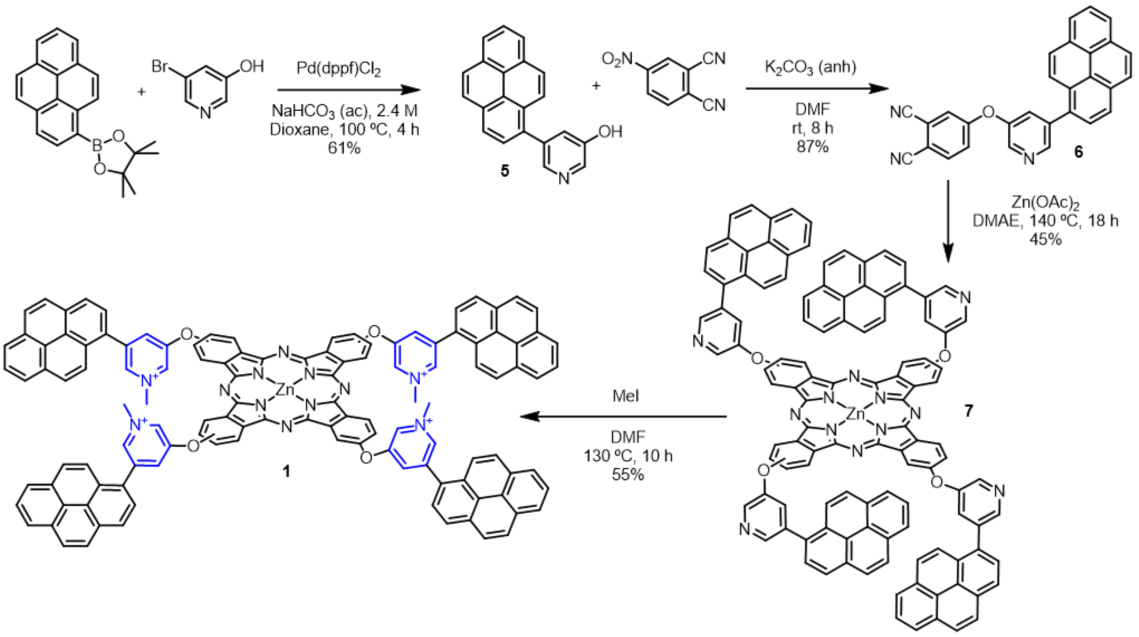

Scheme S1. Synthesis of **1**.

Pyrenyl-1-boronic acid pinacol ester[1]

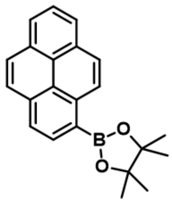

In a round-bottom flask, pyrene-1-boronic acid (1.94 g, 7.88 mmol) and pinacol (2.84 g, 24.0 mmol) were dissolved in 10 mL of THF and 40 mL of diethyl ether, and warmed up to reflux. After 3 h, the reaction mixture was dried under reduced pressure and purified by column chromatography, using heptane/ethyl acetate (20:1) as eluent. The product was obtained as a white solid. Yield: 2.39 g, 92%. Mp: 120 ºC. ^{1}H-NMR (300 MHz, $(CD_3)_2SO$): δ (ppm) = 8.97 (d, J = 9.2 Hz, 1H), 8.45 (d, J = 7.7 Hz, 1H), 8.32 (d, J = 7.7 Hz, 2H), 8.27-8.23 (m, 3H), 8.18 (d, J = 8.9 Hz, 1H), 8.08 (t, J = 7.6 Hz, 1H), 1.43 (s, 12H).

3-hydroxy-5-(1-pyrenyl)pyridine (**5**)

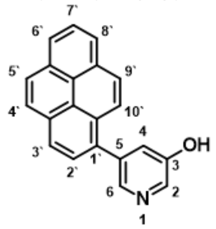

In a round-bottom flask, pyrenyl-1-boronic acid pinacol ester (2.39 g, 7.27 mmol) and 3-bromo-5-hydroxypyridine (1.24 g, 7.10 mmol) were dissolved in 90 mL of 1,4-dioxane under argon atmosphere. $Pd(dppf)Cl_2 \cdot CH_2Cl_2$ (340 mg, 0.42 mmol) was then dissolved and 80 mL of previously deoxygenated aqueous sodium carbonate (2.4 M) was added. The reaction mixture was heated at 100 ºC for 4 hours. After cooling down, both phases were separated and the aqueous phase was washed with THF (3 x 10 mL). The combined organic phases were dried over anhydrous Mg_2SO_4 and dried under reduced pressure. The crude residue was purified by column chromatography, using hexane/ethyl acetate (3:2) as eluent. The product was obtained as a white solid. Yield: 1.28 g, 61%. Mp: >250 ºC. ^{1}H-NMR (300 MHz, $(CD_3)_2SO$): δ (ppm) = 10.23 (s, 1H, OH), 8.39 (d, J = 7.9 Hz, 1H, H_{Ar}), 8.36 (d, J = 6.9 Hz, 1H, H_{Ar}), 8.32 (d, J = 7.8 Hz, 1H, H_{Ar}), 8.29 (d, J = 4.3, 1H, H-2), 8.29 (d, J = 4.2, 1H, H-6, 8.26 (s, 2H, H_{Ar}), 8.22 (d, J = 9.4 Hz, 1H, H_{Ar}), 8.13 (d, J = 7.6 Hz, 1H, H_{Ar}), 8.08 (d, J = 6.9 Hz, 1H, H_{Ar}), 8.04 (d, J = 7.4 Hz, 1H, H_{Ar}), 7.40 (dd, J_1 = 4.3 Hz, J_2 = 4.2 Hz, 1H, H-4).

4-(5-(1-pyrenyl)pyridyl-3-oxy) phthalonitrile (**6**)

SUPPORTING INFORMATION

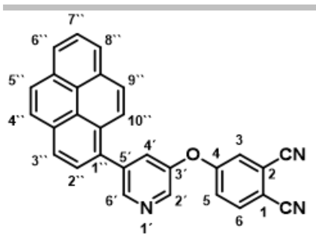

In a round-bottom flask, compound **5** (493.8 mg, 1.67 mmol) and 4-nitrophthalonitrile (323.1 mg, 1.87 mmol) were dissolved in DMF (20 mL) under argon atmosphere. Anhydrous potassium carbonate (1.0 g, 7.24 mmol) was then added in portions and the mixture was stirred at room temperature for 8 hours. The reaction was poured over a water-ice mixture and the precipitate was filtered off and purified by recrystallization from hot ethanol. The product was obtained as a white solid. Importantly, because the assignment of aromatic signals corresponding to the pyrene moiety is extremely difficult once incorporated into the ZnPc macrocycle (i.e., for compounds **1** and **7)**, a full characterization by NMR spectroscopy (including DEPT-135, ^{1}H-^{1}H COSY, ^{1}H-^{13}C HSQC and ^{1}H-^{13}C HSQC spectra) was performed at this stage (see below), helping a lot for the characterization of subsequent products. Yield: 612.4 mg, 87%. Mp: 92 ^{o}C. ^{1}H-NMR (300 MHz, $(CD_3)_2SO$): δ (ppm) = 8.77 (s, 1H, H-2\`), 8.66 (d, *J* = 2.5 Hz, 1H, H-6\`), 8.38 (d, *J* = 8.0 Hz, 1H, H_{Ar}), 8.34 (d, *J* = 7.9 Hz, 1H, H_{Ar}), 8.30 (d, *J* = 7.9 Hz, 1H, H_{Ar}), 8.23 (s, 2H, H_{Ar}), 8.21 (d, *J* = 9.3 Hz, 1H, H_{Ar}), 8.17-8.01 (m, 5H, H_{Ar}, H-3 and H-6), 7.96 (s, 1H, H-4\`), 7.67 (dd, $J_1$ = 8.7 Hz, $J_2$ = 2.4 Hz, 1H, H-5). $^{13}$C-RMN (76 MHz, $(CD_3)_2SO$): δ (ppm) = 160.4 (C-4), 150.8 (C3\`), 147.4 (C-2\`), 140.9 (C-6\`), 137.6 (C-5\`), 136.3 (C-6), 131.9, 130.8, 130.3, 129.1 (C-4\`), 128.3, 128, 127.9, 127.2, 126.5, 125.7, 125.3, 124.9, 124.1, 124, 123.8, 123.1 (C-3), 122.8 (C-5), 116.8 (C-2), 115.8 (CN), 115.3 (CN), 108.9 (C-1). ^{13}C-DEPT-135: (76 MHz, $(CD_3)_2SO$): δ (ppm) = 147.4, 140.9, 136.3, 129.1, 128.3, 127.9, 127.2, 126.5.

<u>[2,(3),9,(10),16,(17),23,(24)-tetrakis-(5-(1-pyrenyl)pyridyl-3-oxy)phthalocyaninato]-zinc(II)-N^{29}, N^{30}, N^{31}, N^{32}</u> (**7**)

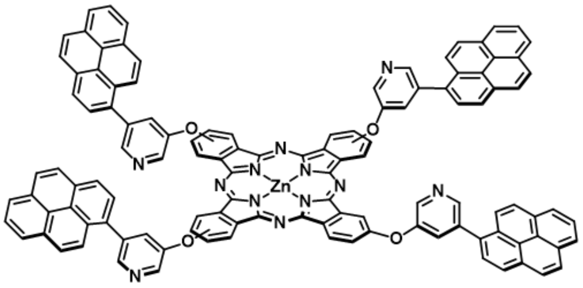

In a sealed tube, phthalonitrile **6** (346 mg, 0.82 mmol) and anhydrous zinc acetate (75 mg, 0.41 mmol) were dissolved in DMAE (5 mL) under argon atmosphere. The reaction was heated to 150 ^{o}C for 24 hours. The reaction mixture was then poured over water/methanol (1:1) mixture and the blue precipitate was filtered off. The green solid was purified by column chromatography using toluene/1,4-dioxane/pyridine (40:10:1) as eluent. The product was obtained as a green solid (162 mg, 45%). Mp: > 250 ^{o}C. ^{1}H-NMR (300 MHz, $(CD_2CD_2)_2O$ (TFA (5%)): δ (ppm) = 12.39 (TFA), 9.2-7.6 (m).* UV-Vis (THF): λ_{max} (nm) (log ε) = 673 (5.24), 608 (4.48), 346 (5.16). MS (MALDI-TOF, DCTB): *m/z* (%) = 1750.4 [M^{+}] (100), 3500.9 [2M^{+}] (4). HRMS (MALDI-TOF, DCTB+PPGNa2000) m/z: exp. Mass = 1748.4179 calc. Mass = 1748.4146. *Due to extremely low solubility and the presence of a regioisomeric mixture, the assignment of signals in the NMR spectrum was impossible for this compound.

<u>[2,(3),9,(10),16,(17),23,(24)-tetrakis-(N-methyl-5-(1-pyrenyl)pyridinium-3-yloxy) phthalocyaninato]zinc(II)-N^{29}, N^{30}, N^{31}, N^{32}, tetraiodine</u> (**1**)

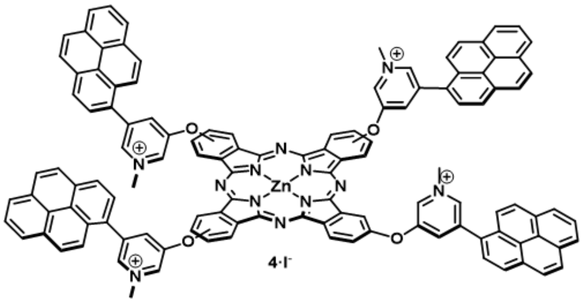

In a sealed tube, the ZnPc **7** (58 mg, 33 µmol) was dissolved in DMF (2 mL) under argon atmosphere, and methyliodide (0.15 mL, 2.4 mmol) was added and heated to 130 ^{o}C. After 10 hours, the reaction mixture was poured onto diethyl ether. The resulting precipitate was isolated by centrifugation and washed with diethyl ether (3x5 mL), water (3x5 mL) and acetone (1x5 mL). The product was obtained as a green solid. Yield: 42.2 mg, 55%. Mp: > 250 ^{o}C. ^{1}H-NMR (500 MHz, $(CD_3)_2SO$): δ (ppm) = 9.59 (m, 4H, H_{Pc}), 9.50 (m, 8H, H_{Py}+H_{Pc}), 9.40 (2s, 4H, H_{Py}), 9.04 – 8.86 (4s (br), 4H, H_{Py}), 8.51 – 8.42 (m, 4H, H_{Ar}), 8.42 – 7.91 (m, 36H, H_{Ar}+H_{Pc}), 4.59 (2s, 12H). ^{13}C-DEPT-135 (126 MHz, $(CD_3)_2SO$): δ (ppm) = 142.4, 136.2, 135.4, 129.2, 127.6, 127.1, 126.6, 125.9, 125.5, 124.1, 113.9, 49.0. UV-Vis (DMSO): λ_{max} (nm) (log ε) = 679 nm (5.20), 611 nm (4.44), 348 nm (5.00). MS (ESI^{+}, methanol) = 452.13 [M]$^{+4}$ (100).

*Due the presence of a regioisomeric mixture and the overlapping of signals, the complete assignment of signals in the ^{1}H- and ^{13}C-NMR spectrum was not possible for this compound.

*Synthesis of **2***

Octakis(1-methyl-3-pyridiniumoxy)-ZnPc tetraiodide (**2**) was synthesized as reported earlier.[2]

SUPPORTING INFORMATION

*Characterization of the ZnPc **1** and its precursors*

Compound **5**

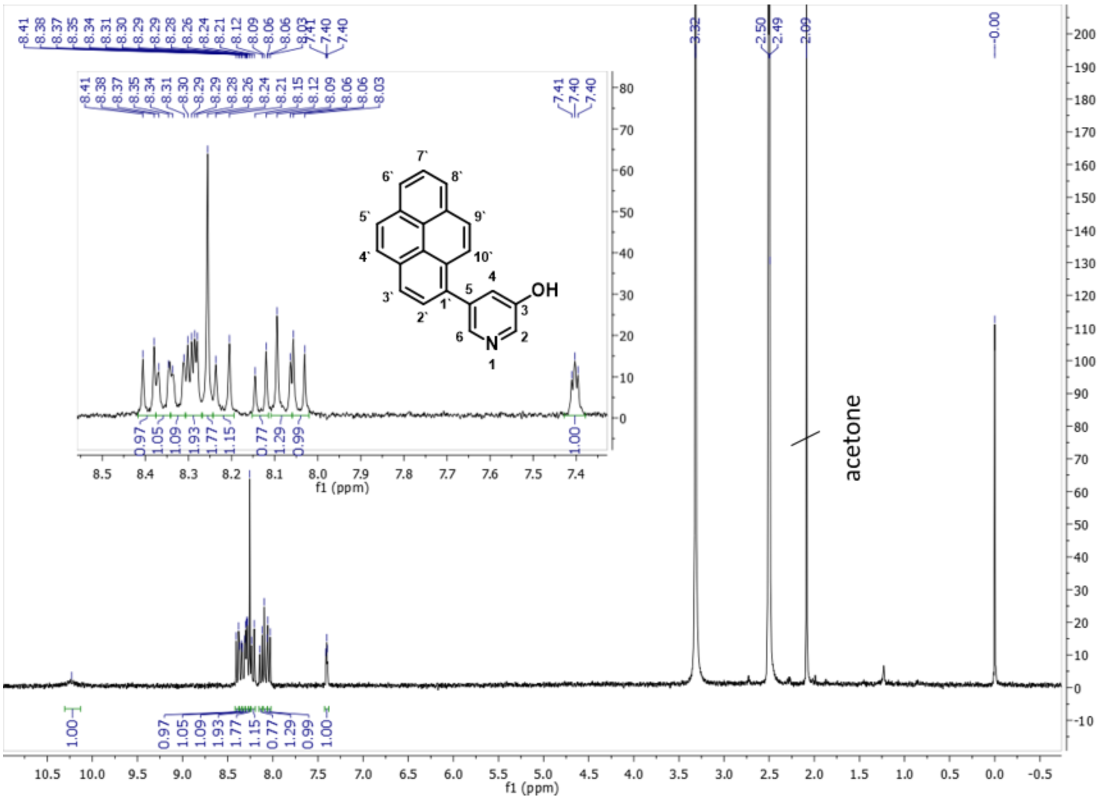

^{1}H-NMR spectrum (300 MHz, DMSO-d_6) of **5**.

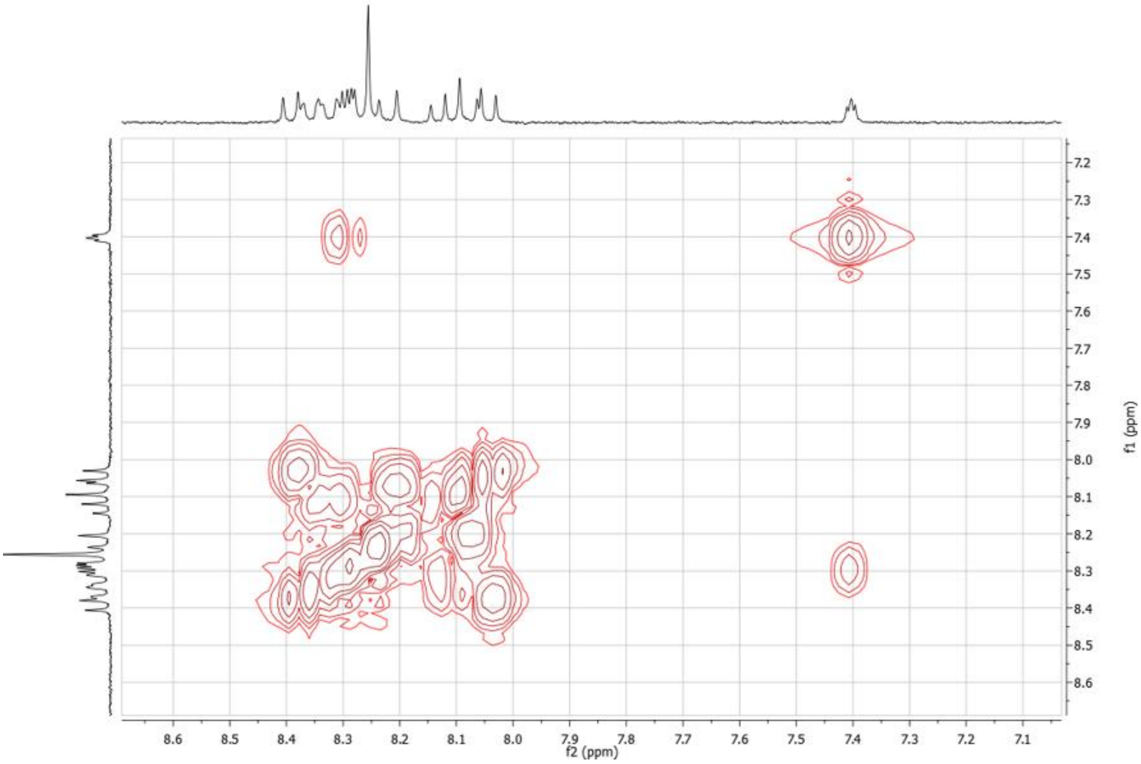

^{1}H-^{1}H COSY spectrum (300 MHz, DMSO-d_6) of **4**.

Compound **6**

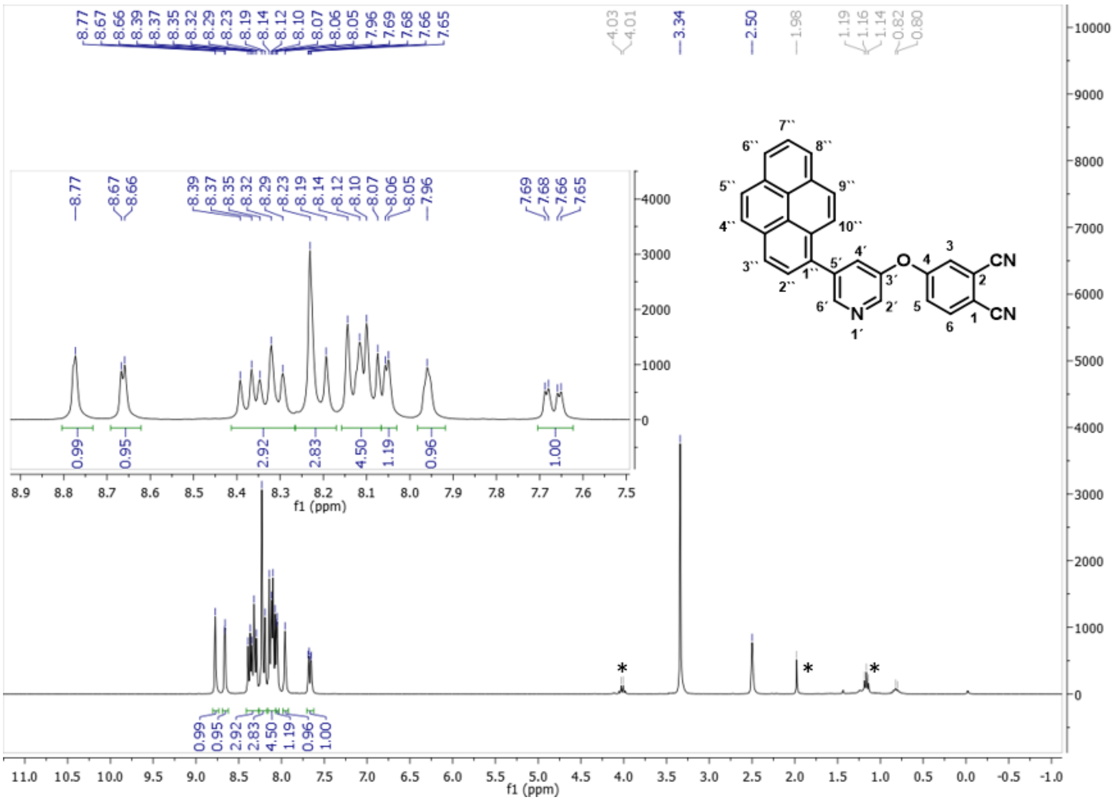

^{1}H-NMR spectrum (300 MHz, DMSO-d6) of **6**. The signals of ethyl acetate, as an impurity, are marked with an asterisk.

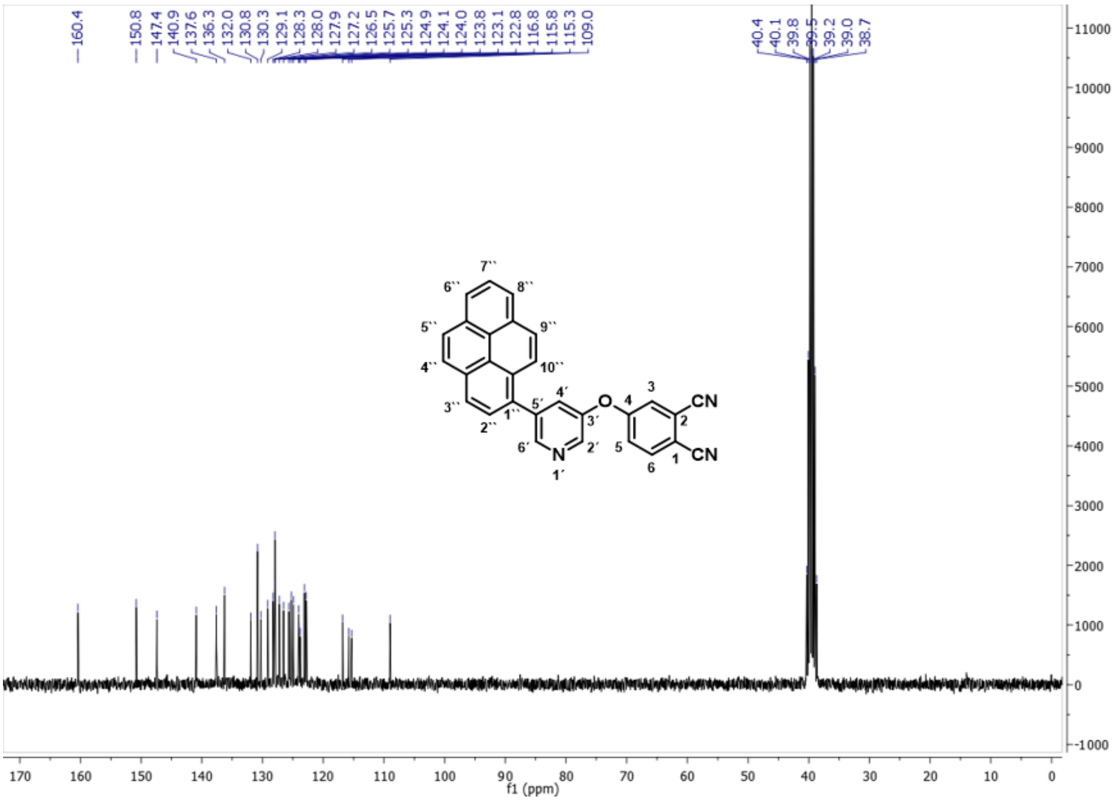

^{13}C-NMR spectrum (76 MHz, DMSO-d6) of **6**.

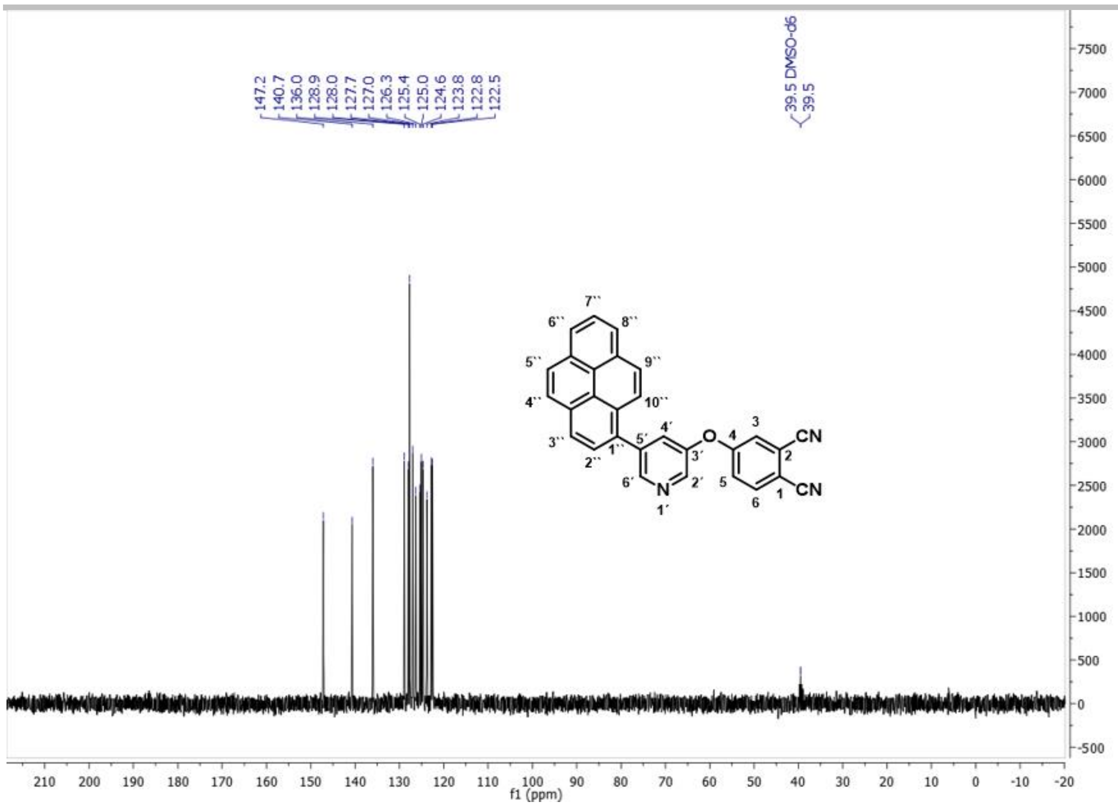

DEPT-135 spectrum (76 MHz, DMSO-d_6) of **6**.

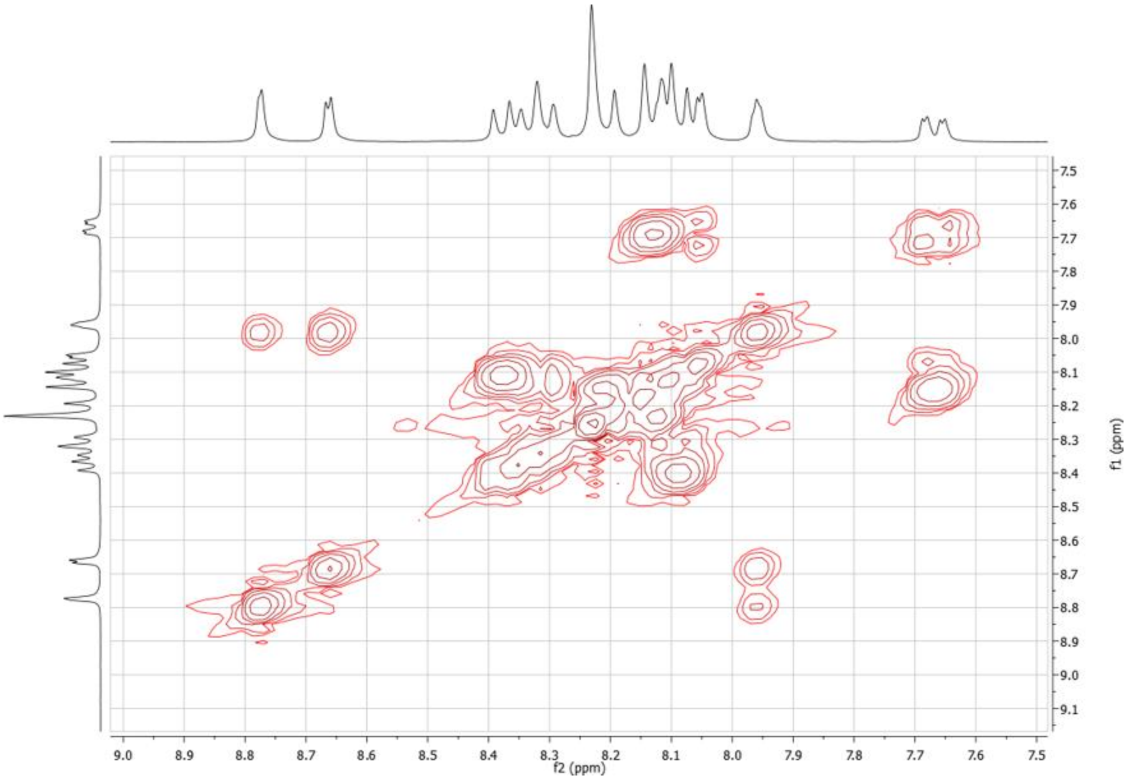

^{1}H-^{1}H COSY spectrum (300 MHz, DMSO-d_6) of **6**.

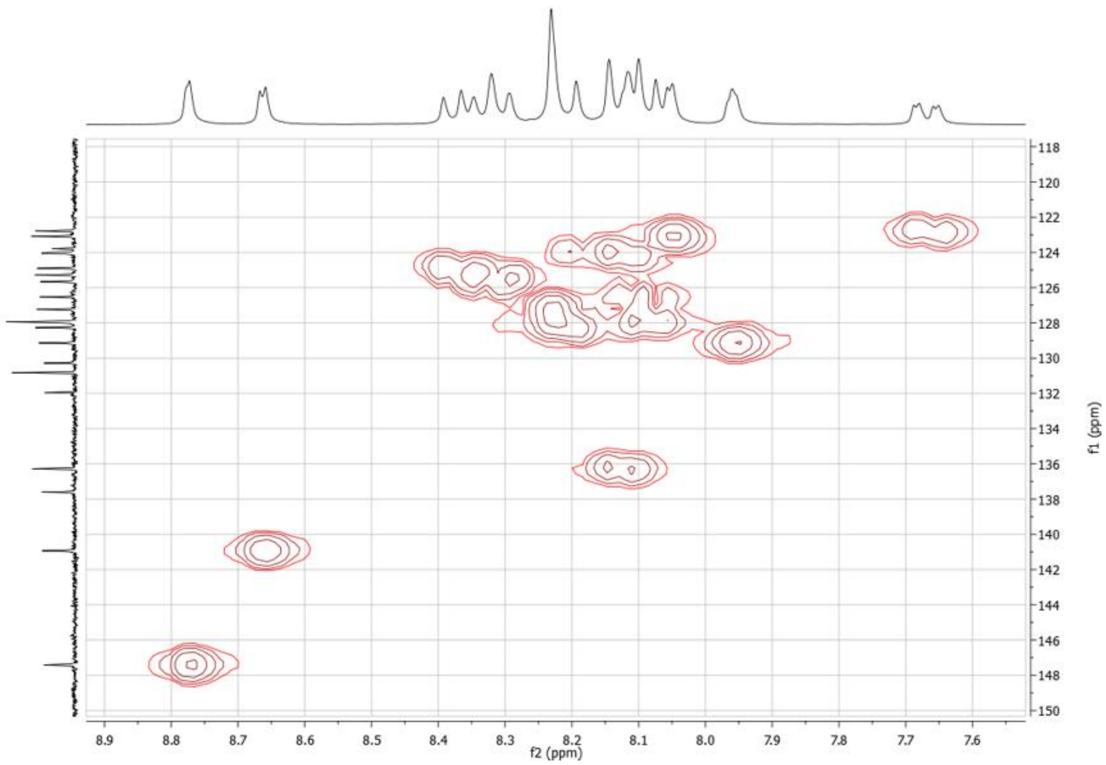

^{1}H-^{13}C HSQC spectrum (300 MHz, DMSO-d_6) of **6**.

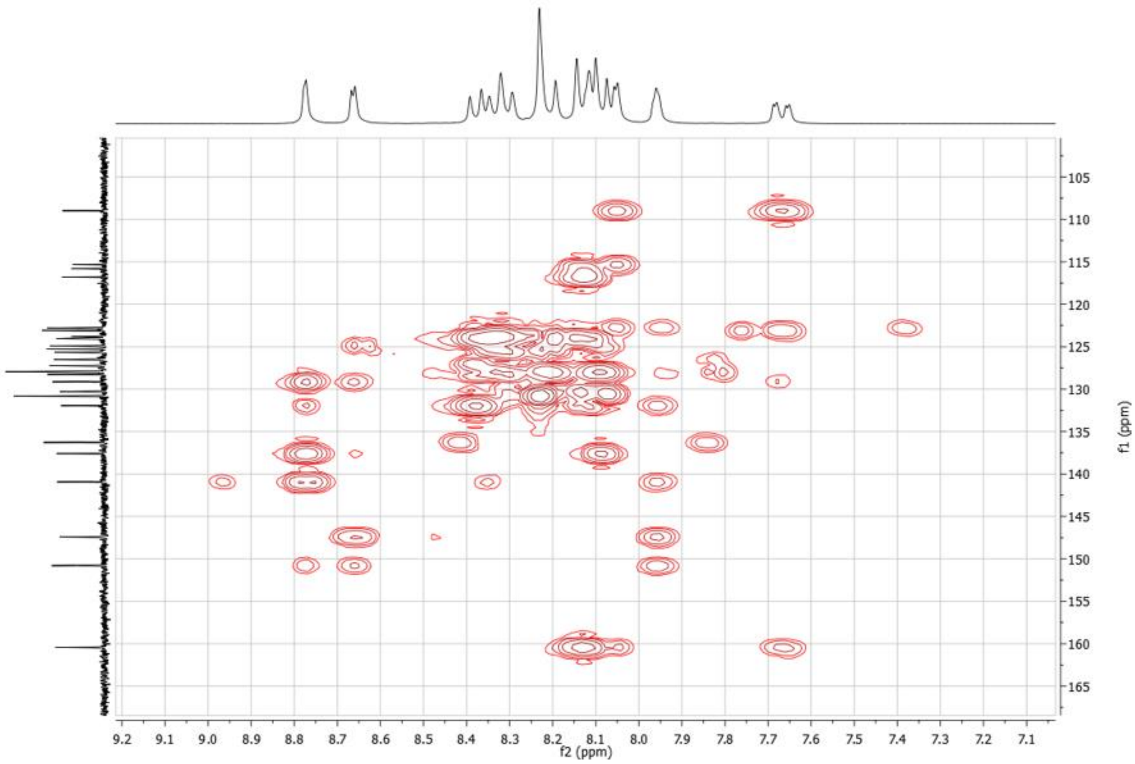

^{1}H-^{13}C HMBC spectrum (300 MHz, DMSO-d_6) of **6**.

Compound **7**

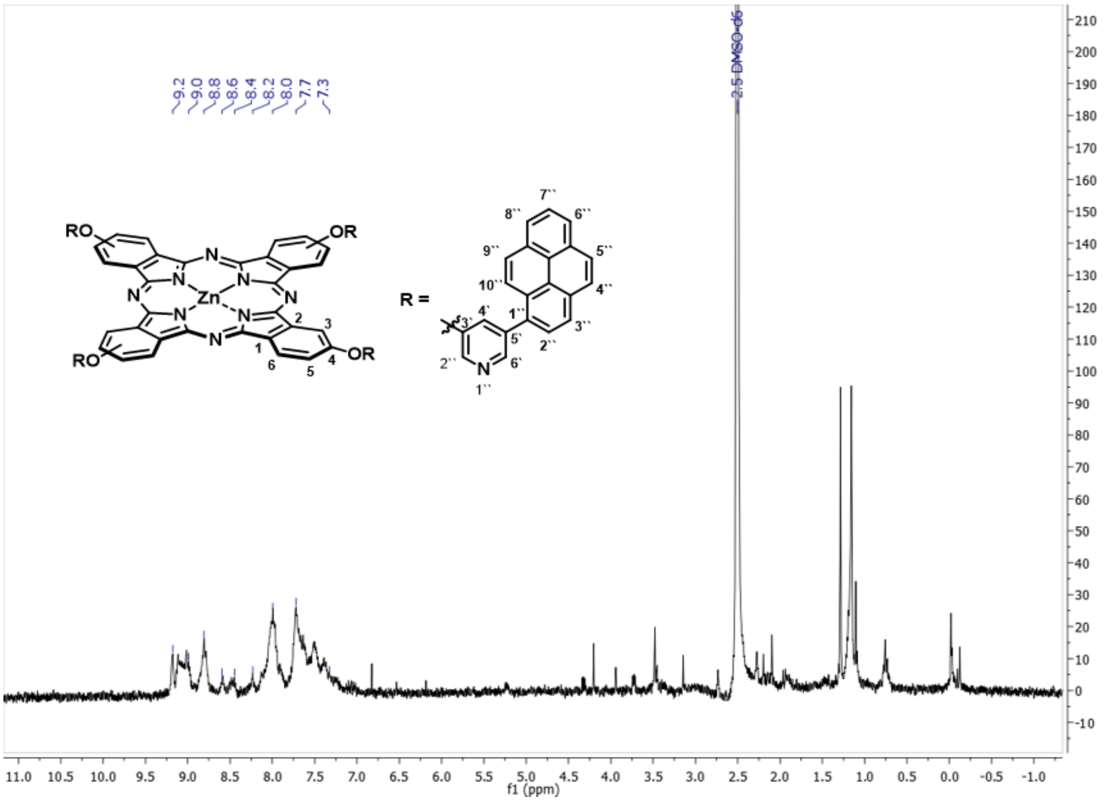

^{1}H-NMR spectrum (300 MHz, DMSO-d_6 – TFA 10%) of **7**.

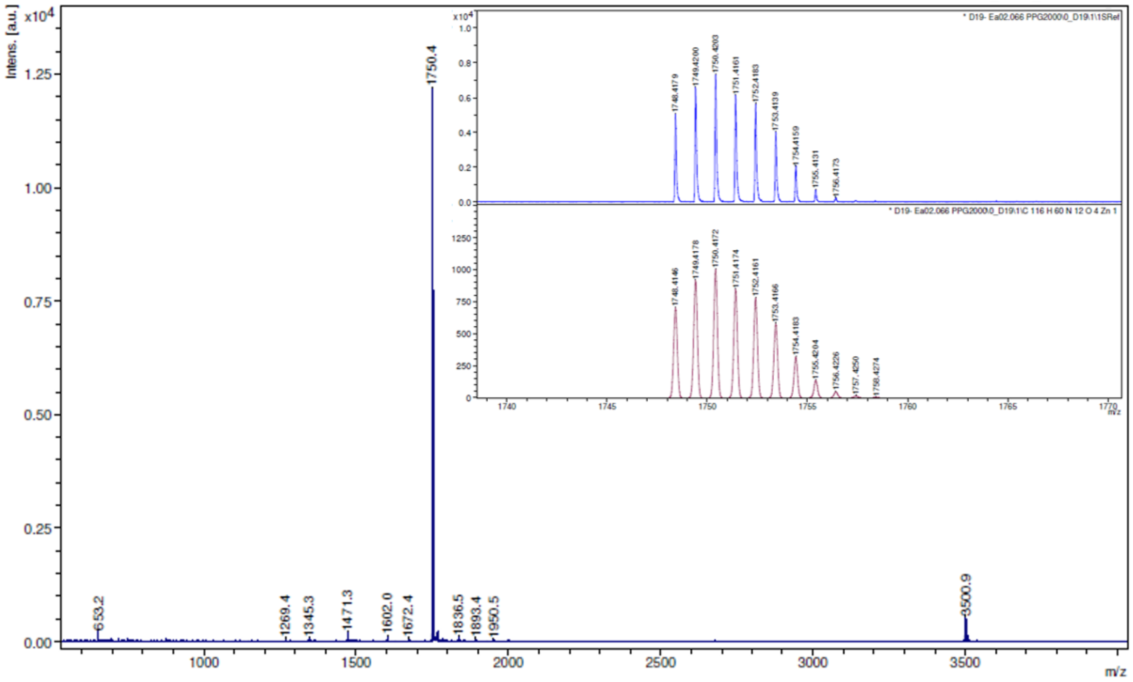

MALDI-TOF (DCTB) spectrum of **7**. Inset: HR-MALDI-HR (DCTB+PPGNa2000) of the peak at *m/z* 1750.4 (top) and isotopic distribution calculated for the ZnPc **7** ($C_{116}H_{60}N_{12}O_4Zn$, bottom).

Compound **1**

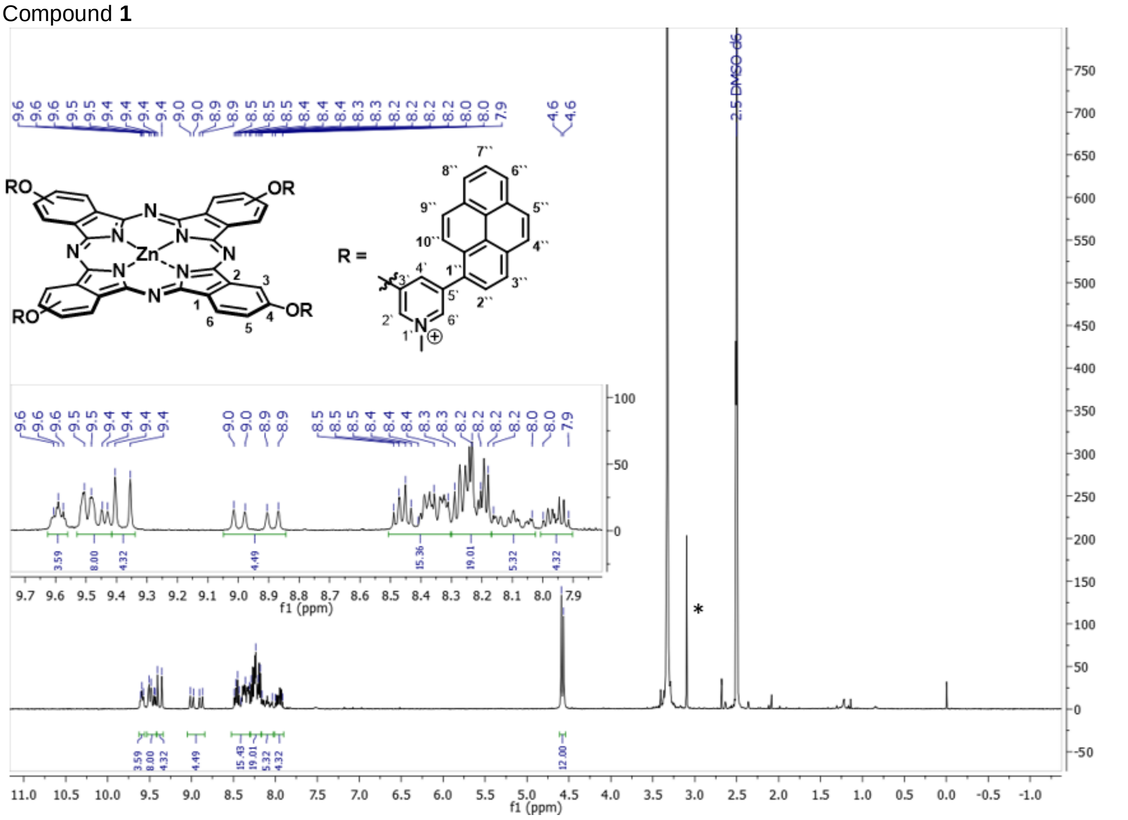

^{1}H-NMR spectrum (500 MHz, DMSO-d_6) of **1**. Signals corresponding to methanol, as an impurity, are marked with an asterisk. The observed multiplicity is due to the presence of different regioisomers.

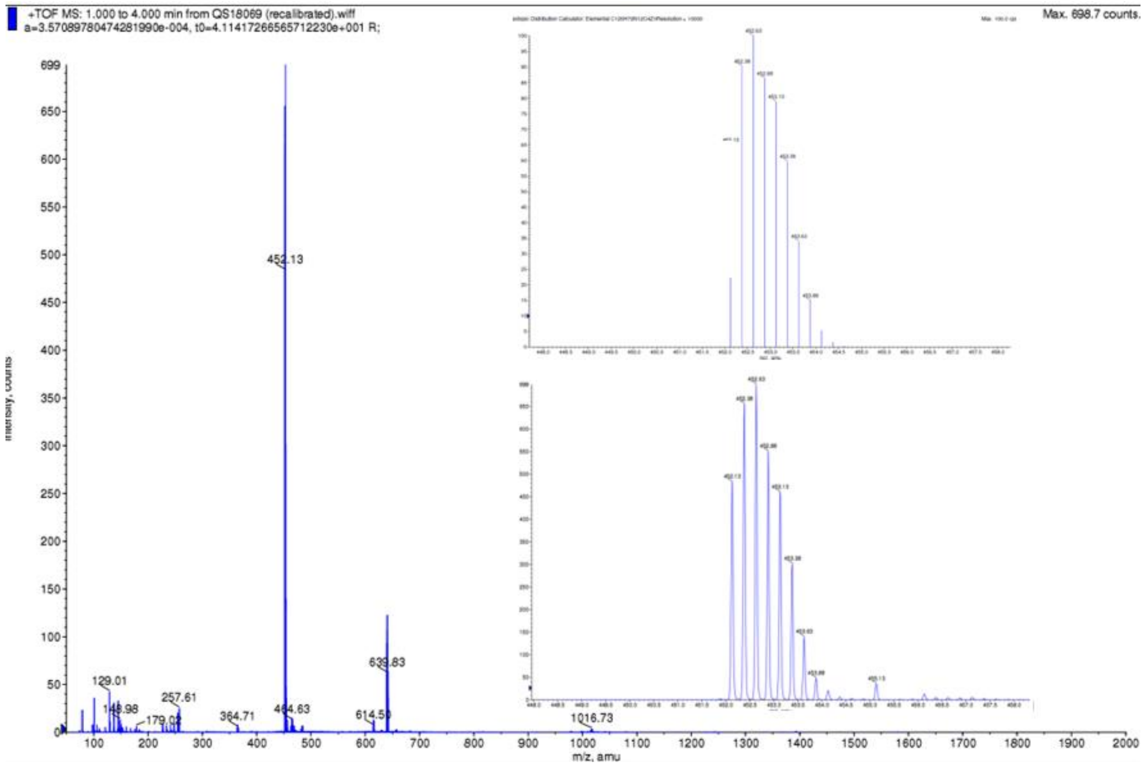

ESI+ (MeOH) spectrum of **1**. Inset: isotopic distribution calculated for the ZnPc **1** ($[C_{120}H_{72}N_{12}O_4Zn]^{4+}$, top) and detail of ESI+ peak at *m/z* 452.1 (bottom).

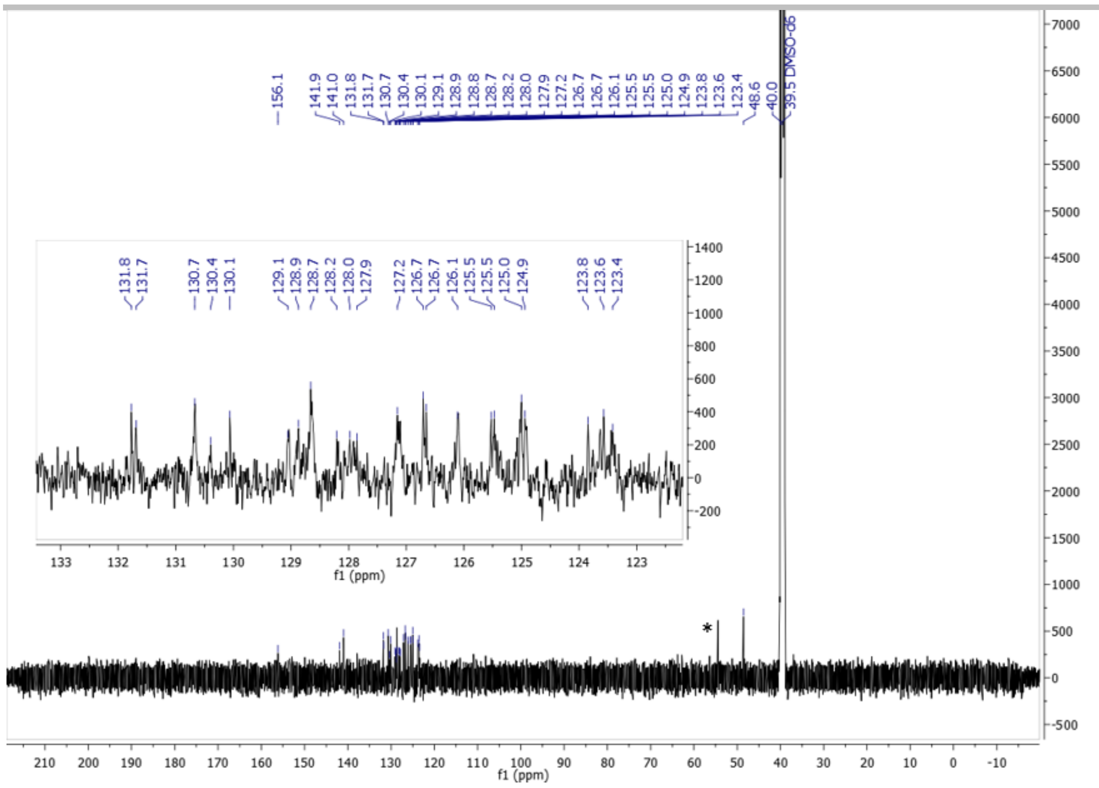

^{13}C-NMR spectrum (125 MHz, DMSO-d$_6$) of **1**. Signals corresponding to methanol, as an impurity, are marked with an asterisk. The observed multiplicity and low resolution is due to the presence of different regioisomers.

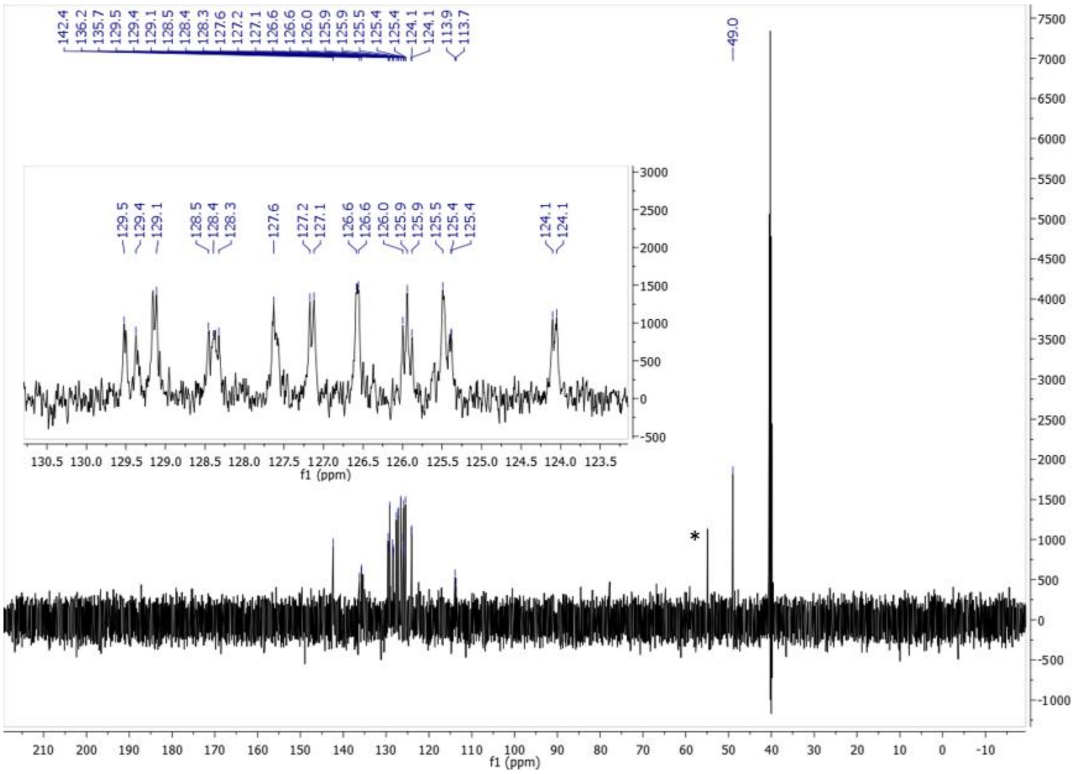

DEPT-135 spectrum (125 MHz, DMSO-d$_6$) of **1**. Signals corresponding to methanol, as an impurity, are marked with an asterisk. The observed multiplicity and low resolution is due to the presence of different regioisomers.

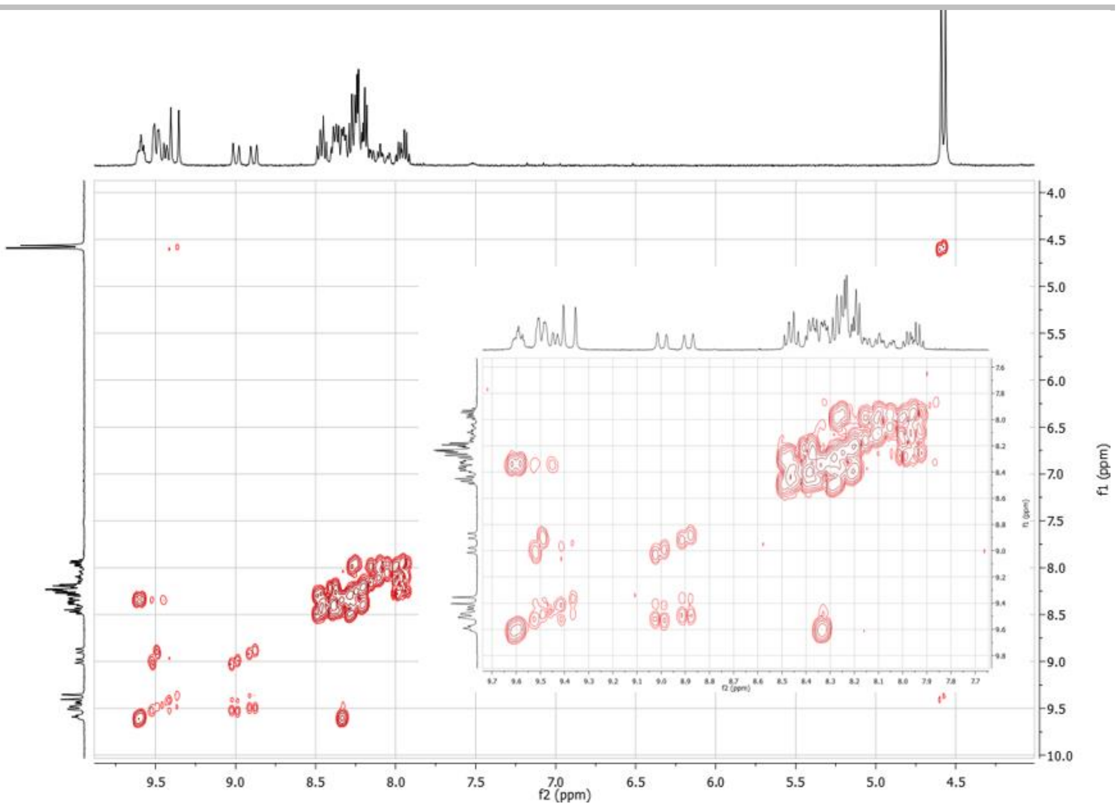

^1H-^1H COSY spectrum (500 MHz, DMSO-d_6) of **1**. Inset: detail of the aromatic region of the spectrum.

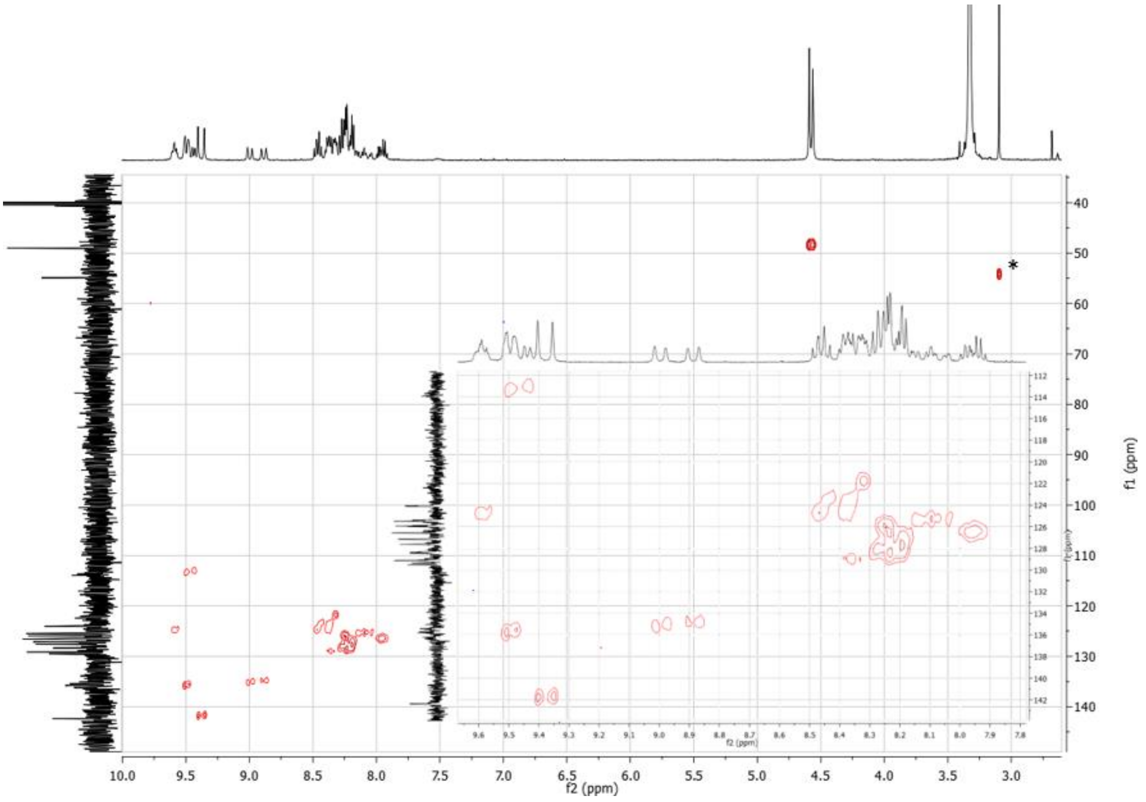

^1H-DEPT-135 HSQC spectrum (500 MHz, DMSO-d_6) of **1**. Inset: detail of the aromatic region of the spectrum. Signals corresponding to methanol, as an impurity, are marked with an asterisk. The observed multiplicity is due to the presence of different regioisomers.

*Synthesis and characterization of the anionic fullerene derivatives **3** and **4**.*

The synthesis of compounds **2** and **3** (*trans3* isomer) was achieved by adapting a previously described methodology (Scheme S2).

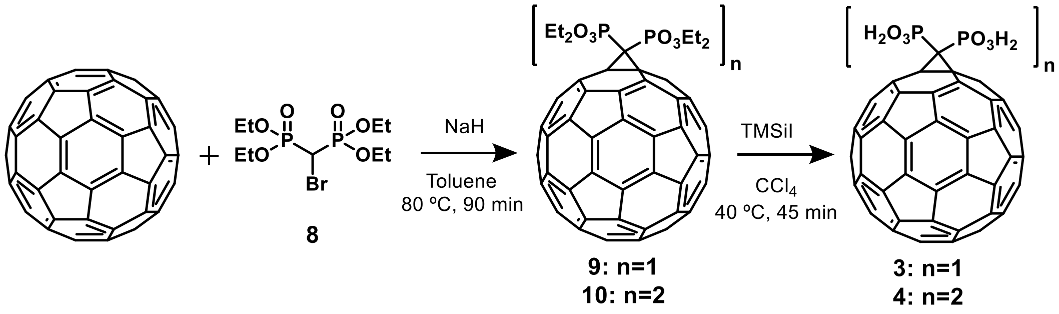

Scheme S2. Synthesis of fullerenes **3** and **4**. Note that **10** was obtained as a mixture of regioisomers, purified by column chromatography (eluent gradient, from chloroform to chloroform/THF 1:1). The trans3 isomer was the majoritarian species, therefore being the only one hydrolysed, leading to the compound (**3**) that has been utilized in the subsequent complexation studies with the ZnPc **1**.

Tetraethyl bromomethylenediphosphonate (**8**)[3]

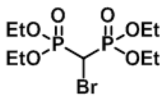

In a 100 mL round-bottomed flask, *n*-buthyllithium 1.6 M in hexane (6.9 mL, 11.0 mmol) was dissolved into 20 mL of freshly distilled THF at -78 ºC. Subsequently, a solution of diisopropylamine (1.6 mL, 11.4 mmol) in 10 mL of freshly distilled THF was added and stirred for 10 min. Thereafter, a solution of tetraethyl methylenediphosphonate (1.2 mL, 4.8 mmol) in 10 mL of freshly distilled THF was added dropwise. When the addition was completed, the reaction was left to warm up to room temperature, followed by the addition of a solution of dibromotetrachloroethane (1.8 g, 5.5 mmol) in 10 mL of freshly distilled THF. After 30 min, the reaction was poured over an H_2SO_4 2M/DCM (1:1) mixture (60 mL) and the phases were separated. The aqueous phase was washed with DCM (2 x 25 mL) and the combined organic phases were dried over anhydrous Mg_2SO_4 and, the solvent was removed in vacuo. The dark yellow oil crude was purified by column chromatography, employing ethylacetate as eluent, resulting into a yellowish oil. Yield: 1.34 g, 75%. ^{1}H-NMR (300 MHz, $CDCl_3$): δ (ppm) = 4.36-4.20 (m, 8H, -OCH_2-), 3.87 (t, *J* = 16.9 Hz, 1H, -CHBr), 1.38 (t, *J* = 7.1 Hz, 12H, -CH_3). ^{31}P-NMR (122 MHz, $CDCl_3$, ref. H_3PO_4 = 0.00 ppm): δ (ppm) = 13.29 (s).

General procedure for the Bingel-Hirsch reaction[4]

In a 1 L round bottom flask, dry toluene (400 mL) were deaereated, followed by the addition of C_{60} (510 mg, 0.74 mmol) and NaH 60% in mineral oil (200 mg, 5 mmol), previously washed with toluene. The resulting solution was warmed up to 80 ºC, followed by the dropwise addition of **8** (659 mg, 1.79 mmol) in anhydrous toluene (5 mL), resulting in gas evolution (H_2). The reaction mixture was left stirring for 90 min, filtered off afterwards and the resulting solution was dried under reduced pressure.

Monoadduct (**9**)

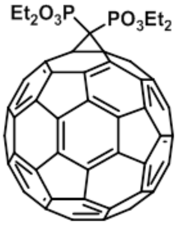

The aforementioned reaction crude was purified by column chromatography, with $CHCl_3$ as eluent, **9** eluting as first fraction. Yield: 86.7 mg, 12 %. HPLC retention time: 18.5 min. Mp: 252-254 ºC. ^{1}H-NMR (300 MHz, $CDCl_3$): δ (ppm) = 4.48 (q, *J* = 7.2 Hz, 8H, -OCH_2), 1.52 (t, *J* = 7.0 Hz, 12H, -CH_3). ^{31}P-NMR (122 MHz, $CDCl_3$, ref. H_3PO_4 = 0.00 ppm): δ (ppm) = 14.82 (s). MS (MALDI-TOF, DCTB): *m/z* (%) = 1006.1 $[M]^+$ (100).

Bisadduct (**10**)

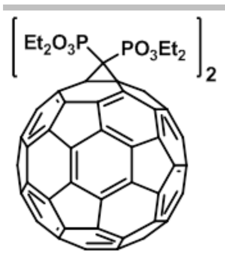

The aforementioned reaction mixture was purified by chromatography column, employing a solvent gradient, from $CHCl_3$ to $CHCl_3$/THF (1:1) as eluent, eluting as second (**10**-*trans1*), third (**10**-*trans2*), fourth (**10**-*trans3*) and fifth (**10**-*trans4* + **10**-*e*) fractions. The latter was further purified, yielding small amounts suitable for characterization purposes. MS (MALDI-TOF, DCTB): *m/z* (%) = 1292.1 $[M]^+$ (100). HRMS (MALDI-TOF, DCTB) m/z: exp. Mass = 1292.1433 calc. Mass = 1292.1465.

10-*trans1*: Yield: 5.2 mg, 0.6 %. HPLC retention time: 20.8 min. ^{1}H-NMR (300 MHz, $CDCl_3$): δ (ppm) = 4.58 (q, *J* = 7.4 Hz, 16H, $-OCH_2$), 1.59 (t, *J* = 7.1 Hz, 24H, $-CH_3$). ^{31}P-NMR (122 MHz, $CDCl_3$, ref. H_3PO_4 = 0.00 ppm): δ (ppm) = 16.07 (s).

10-*trans2*: Yield: 38.7 mg, 4 %. HPLC retention time: 22.4 min. ^{1}H-NMR (300 MHz, $CDCl_3$): δ (ppm) = 4.87-4.23 (m, 16H, $-OCH_2$), 1.59 (m, 24H, $-CH_3$). ^{31}P-NMR (122 MHz, $CDCl_3$, ref. H_3PO_4 = 0.00 ppm): δ (ppm) = 16.12 (d, *J* = 8.1 Hz), 15.38 (d, *J* = 8.0 Hz).

10-*trans3*: Yield: 85.3 mg, 9 %. HPLC retention time: 26.1 min. ^{1}H-NMR (300 MHz, $CDCl_3$): δ (ppm) = 4.62-4.24 (m, 16H, $-OCH_2$), 1.58 (t, *J* = 7.6 Hz, 6H, $-CH_3$), 1.49 (t, *J* = 7.8 Hz, 6H, $-CH_3$), 1.48 (t, *J* = 7.8 Hz, 6H, $-CH_3$). ^{31}P-NMR (122 MHz, $CDCl_3$, ref. H_3PO_4 = 0.00 ppm): δ (ppm) = 15.59 (s).

10-*trans4* + **10**-*e*: Yield: 67.2 mg, 7 %.

10-*trans4*: HPLC retention time: 29.4 min. ^{1}H-NMR (300 MHz, $CDCl_3$): δ (ppm) = 4.53-4.07 (m, 16H, $-OCH_2$), 1.54-1.14 (m, 24H, $-CH_3$). ^{31}P-NMR (122 MHz, $CDCl_3$, ref. H_3PO_4 = 0.00 ppm): δ (ppm) = 15.55 (dd, J_1 = 14.6 Hz, J_2 = 10.2 Hz).

10-*e*: HPLC retention time: 29.4,* 31.4 min. ^{1}H-NMR (300 MHz, $CDCl_3$): δ (ppm) = 4.46-4.23 (m, 16H, $-OCH_2$), 1.49-1.26 (m, 24H, $-CH_3$). ^{31}P-NMR (122 MHz, $CDCl_3$, ref. H_3PO_4 = 0.00 ppm): δ (ppm) = 15.58 (s), 15.55* (dd, J_1 = 14.6 Hz, J_2 = 10.2 Hz). The impurity corresponding to **10**-*trans4* is marked with an asterisk.

Phosphonic acid fullerene derivative, monoadduct (**3**)

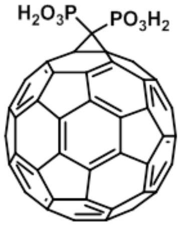

Fullerene **9** (21.8 mg, 0.022 mmol) was dissolved in CCl_4 (5 mL) and was warmed up to 40 ºC. Trimethylsilyl iodide was added dropwise (10 µL, 0.7 mmol) and was reacted for 45 min. Water (10 mL) was added at rt and was left for additional 30 min. The two phases were separated and the organic layer was washed with water (2 x 5 mL). Combined organic phases were then subjected to ion exchange with Amberlite IR-120 (plus) resine, and subsequently evaporation of the solvent under reduced pressure. The resulting solid was crushed with acetone. Yield: 18.7 mg, 96 %. Mp: >250 ºC. MS (ESI^-, MeOH)*: *m/z* (%) = 446.0 $[M-2H]^{2-}$ (100), 892.9 $[M-H]^-$ (3). HRMS (ESI^-, MeOH)* *m/z*: $[M-2H]^{-2}$ calcd for $C_{61}H_2O_6P_2$, 445.9669; found, 445.9670. *The sample was dissolved in DMSO and subsequently diluted in MeOH.

Phosphonic acid fullerene derivative, bisadduct (**4** - *trans3* isomer)

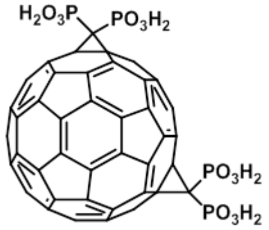

Fullerene **10**-*trans3* (175.3 mg, 0.135 mmol) were dissolved into CCl_4 (10 mL) and was warmed up to 40 ºC. Trimethylsilyl iodide was added dropwise (0.4 mL, 0.25 mmol) and was reacted for 45 min. Water (10 mL) was added at rt and was left for additional 30 min. The two phases were separated and the organic layer was washed with water (2 x 5 mL). Combined organic phases were then subjected to ion exchange with Amberlite IR-120 (plus) resine, and subsequently evaporation of the solvent under reduced pressure. The resulting solid was crushed with acetone. Yield: 125.5 mg, 87 %. Mp: >250 ºC. MS (ESI^-, MeOH)*: *m/z* (%) = 355.0 $[M-3H]^{3-}$ (36), 493.0 $[M-2H-PO_3]^{2-}$ (85), 532.9 $[M-2H]^{2-}$ (100), 1066.9 $[M-H]^-$ (2). HRMS (ESI^-, MeOH)* *m/z*: $[M-2H]^{-2}$ calcd for $C_{62}H_6O_{12}P_4$, 532.9410; found, 532.9405. *The sample was dissolved in DMSO and subsequently diluted in MeOH.

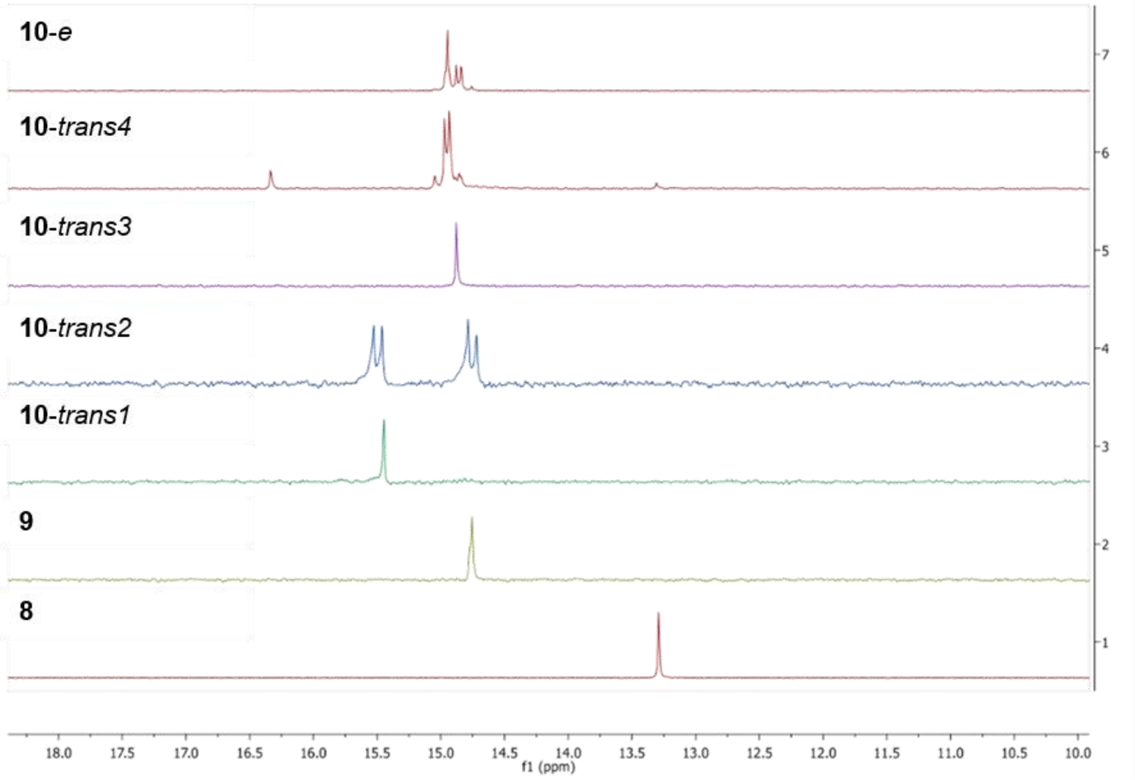

Figure S1. ^{31}P-NMR spectra (122 MHz, $CDCl_3$, ref. H_3PO_4 = 0.00 ppm) of **8**, **9**, and the regioisomers of **10**.

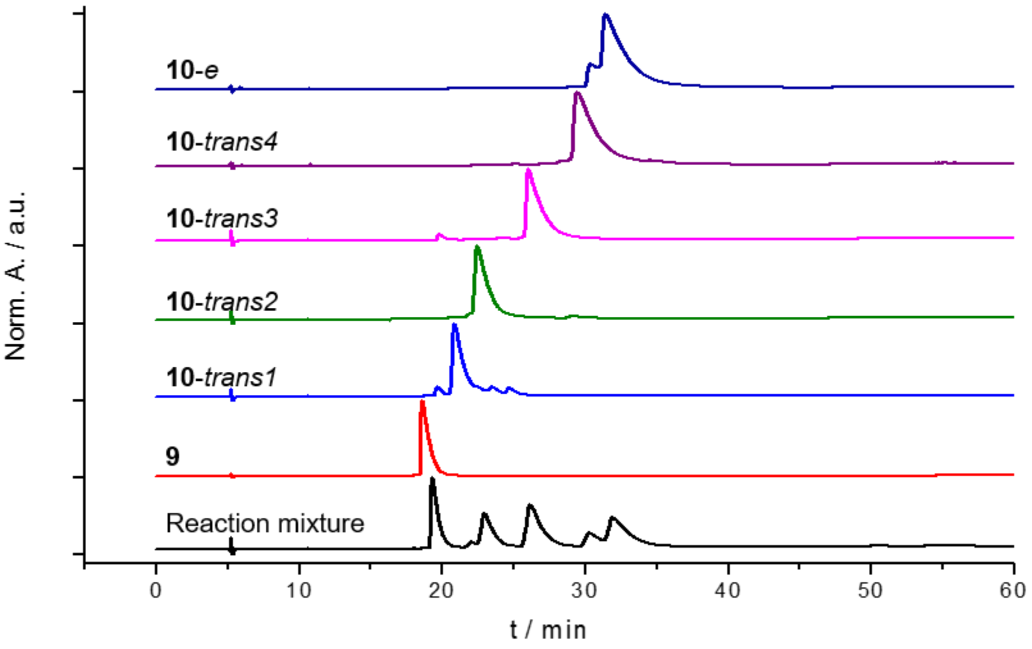

Figure S2. HPLC chromatograms of **9**, five different regioisomers of **10**, and the reaction mixture. Conditions: Buckyprep column, using a linear gradient of eluents: t_0 = toluene (t_0); t_{40} = toluene/THF (85:15).

a)

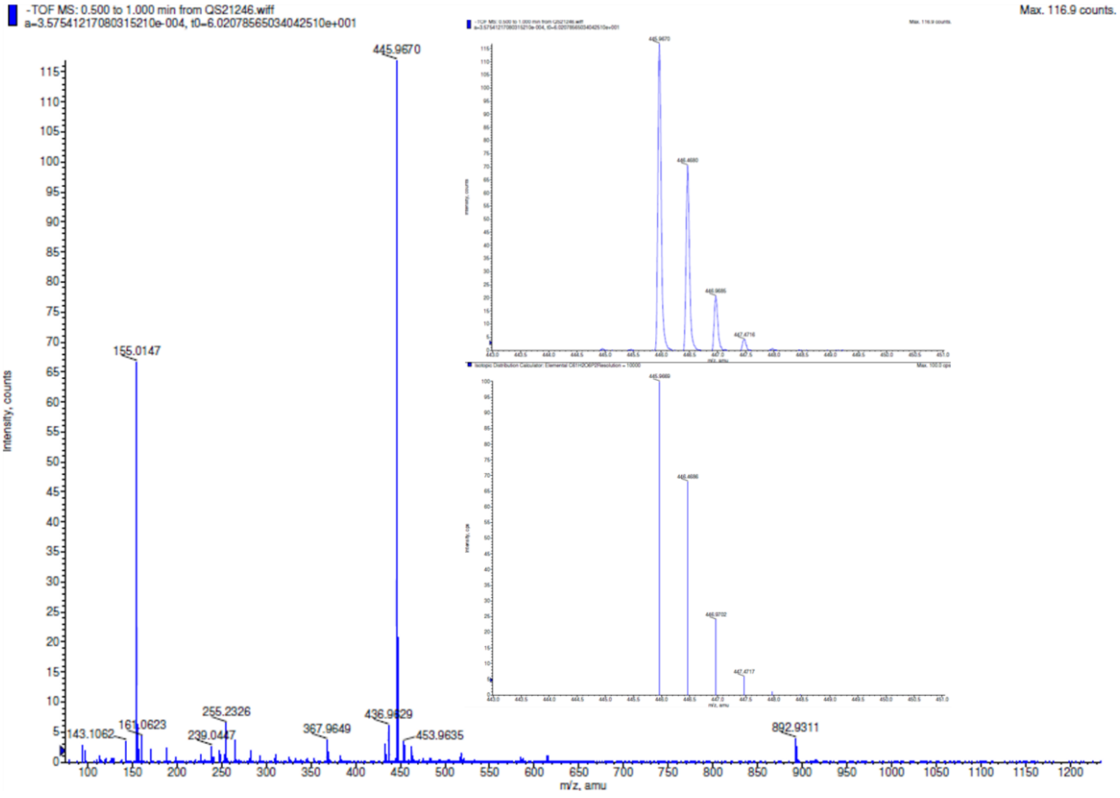

b)

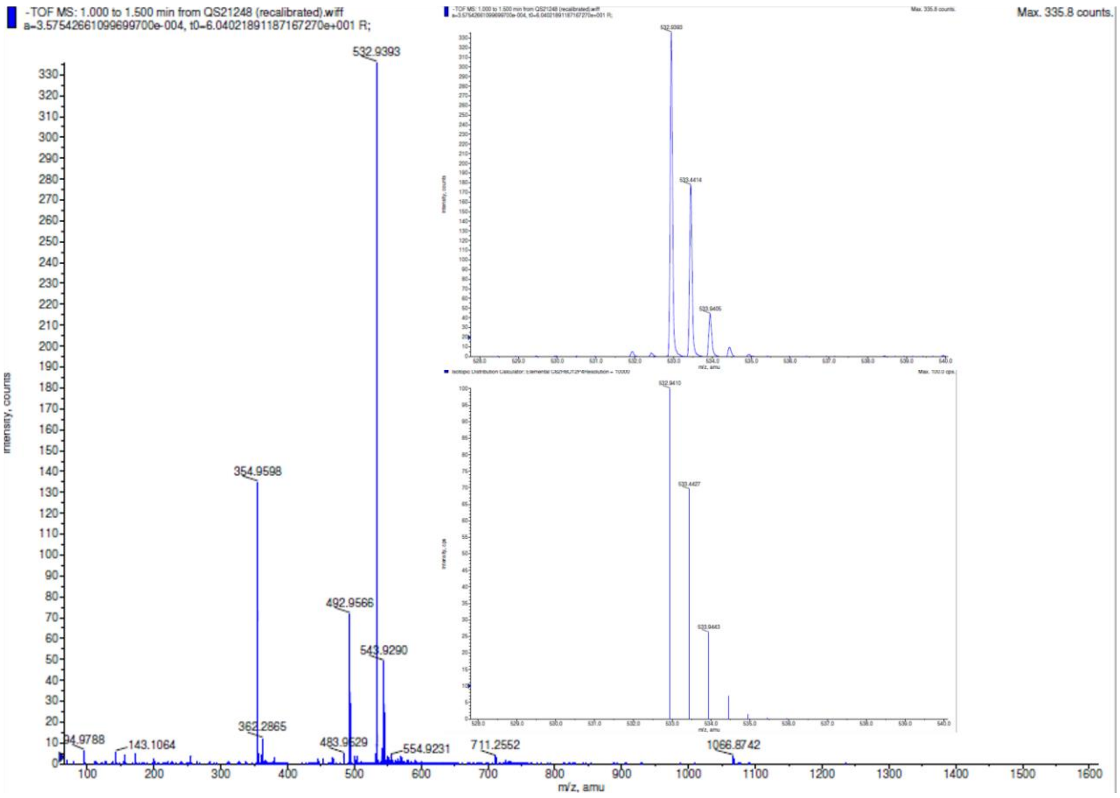

Figure S3. a) ESI- (MeOH) spectrum of **3**. Inset: isotopic distribution calculated for the molecular ion of **3** ([$C_{61}H_2O_6P_2$]$^{2+}$, top) and detail of ESI+ peak at 445.9 *m/z* (bottom). b) ESI- (MeOH) spectrum of **4**. Inset: isotopic distribution calculated for the molecular ion of **4** ([$C_{62}H_6O_{12}P_4$]$^{2+}$, top) and detail of ESI+ peak at 532.9 *m/z* (bottom).

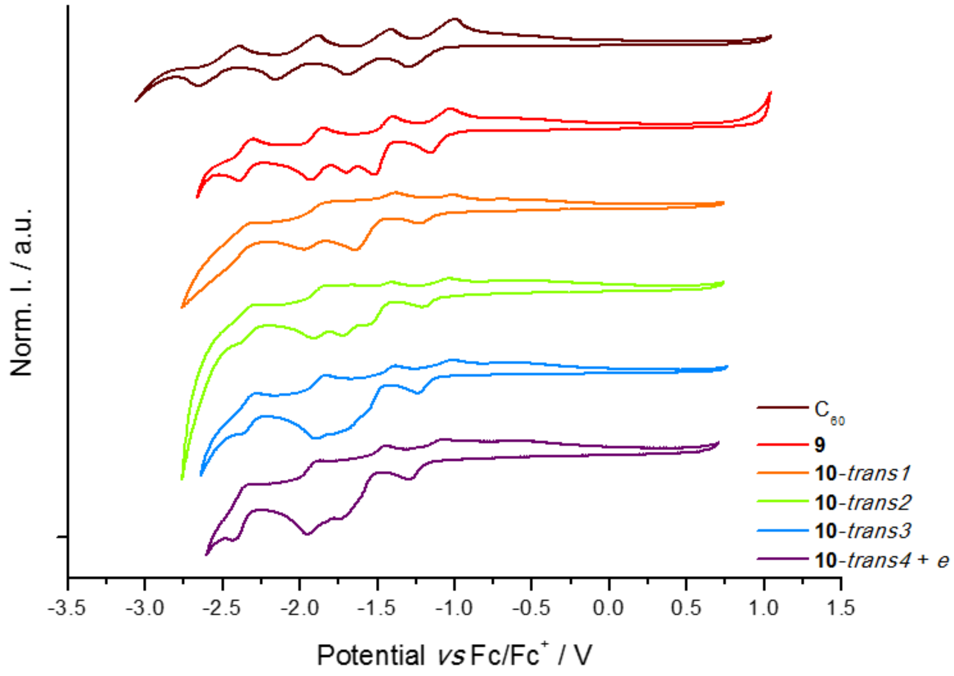

Figure S4. Cyclic voltammograms of C_{60}, **9**, and five different regioisomers of **10** in an anhydrous *o*-DCB/dioxane (4:1) mixture, employing $TBAPF_6$ 0.1 M as supporting electrolyte, Pt electrode as working electrode, Pt wire as counter electrode and $Ag/AgNO_3$ non-aqueous electrode as pseudo-reference electrode. Spectra corrected to Fc/Fc^+ as reference.

Table S1. Redox Potentials of C_{60}, **9**, and **10** obtained by cyclic voltammetry.

Compound	1st $E_{1/2}$	2nd $E_{1/2}$	Irrev. Red.	3rd $E_{1/2}$	4th $E_{1/2}$
C_{60}	-1.11	-1.52	-	-1.99	-2.49
**9**	-1.19	-1.53	-1.68	-1.95	-2.42
**10**-*trans1*	-1.19	-1.50	-1.70	-1.89	-2.36
**10**-*trans2*	-1.15	-1.49	-1.69	-1.88	-2.35
**10**-*trans3*	-1.18	-1.46	-1.68	-1.85	-2.34
**10**-*trans4 + e*	-1.24	-1.51	-1.67	-1.90	-2.37

Results and Discussion

Aggregation studies of the ZnPcs

Organic solvent effect

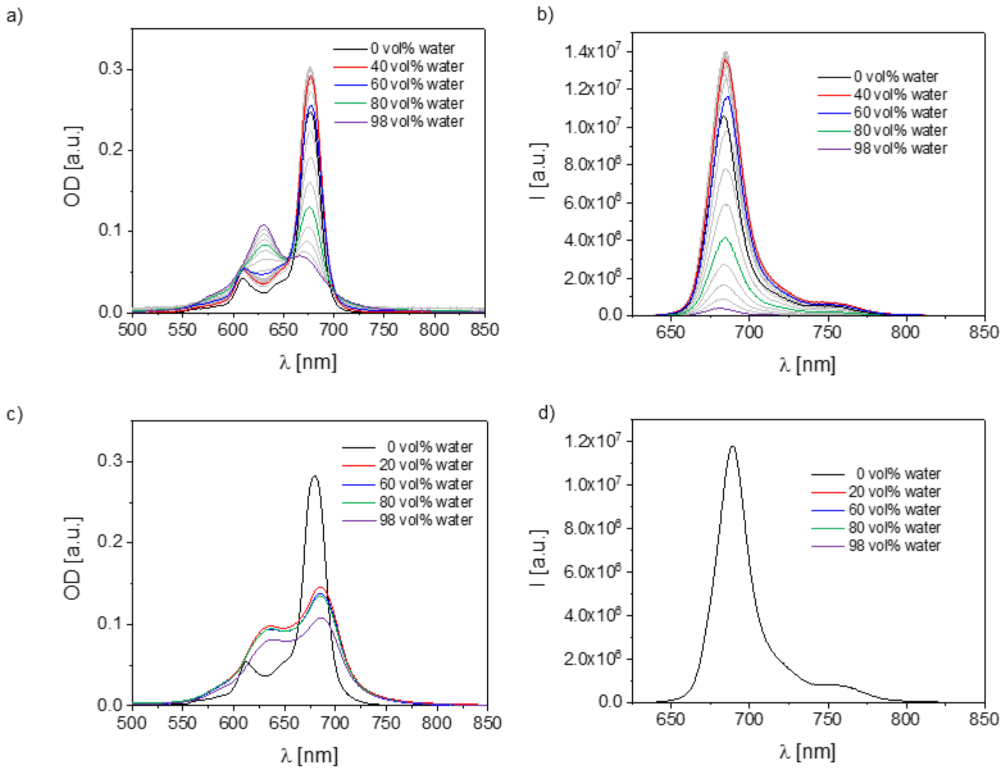

Figure S5. Absorption and emission spectra of **2** (a, b) and **6** (c, d) at 2.0 x 10^{-6} M recorded for different solvent ratios of DMSO and water, from 100 vol% DMSO to 98 vol% water. Emission recorded at λ_{exc} = 615 nm.

Light scattering

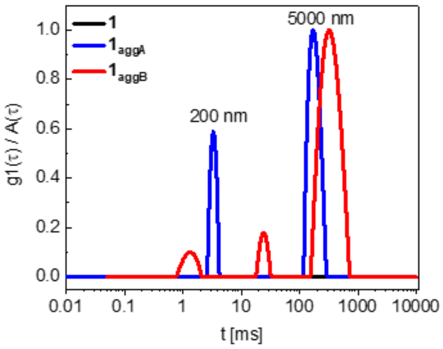

Figure S6. Distribution function of relaxation times for **1** at increasing water content (curves from θ = 90°). Hydrodynamic radii extracted from extrapolation q against 0.

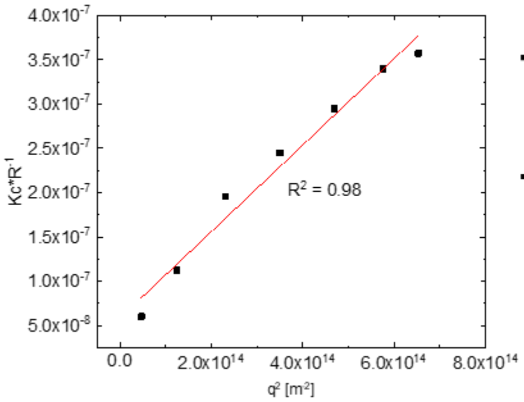

Sample	R_G / nm	R_H / nm	R_G/R_H
**1-4** (1:1)	158	94.7	1.68

*values obtained by extrapolation of q against 0

Figure S7. Scattering intensity of **1-4** (1:1) plotted against q^2.

Temperature effect

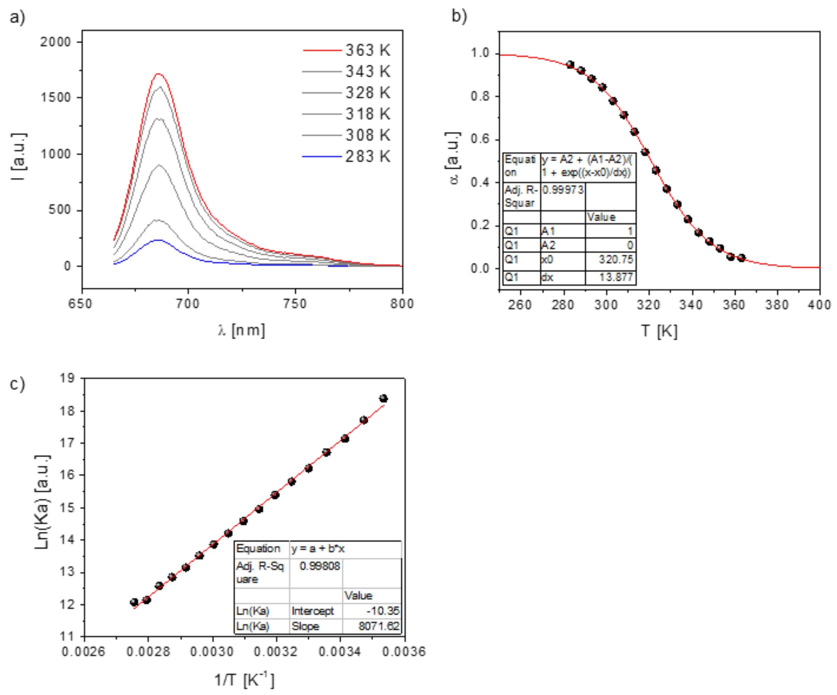

Figure S8. Temperature-dependent fluorescence spectra (a) of $\mathbf{1}_{\mathbf{aggA}}$ in DMSO/water (40:60) at 1.0 x 10^{-6} M from 363 (red) to 293 K (blue). Fluroescence recorded at λ_{exc} = 615 nm. Data recorded every 5 K, applying a temperature gradient of -2 K/min. b) Fraction of aggregated species *versus* temperature and fitting of the spectral changes to the Boltzmann equation (R^2 = 0.9997). c) Van't Hoff plot for **1** in the aforementioned conditions.

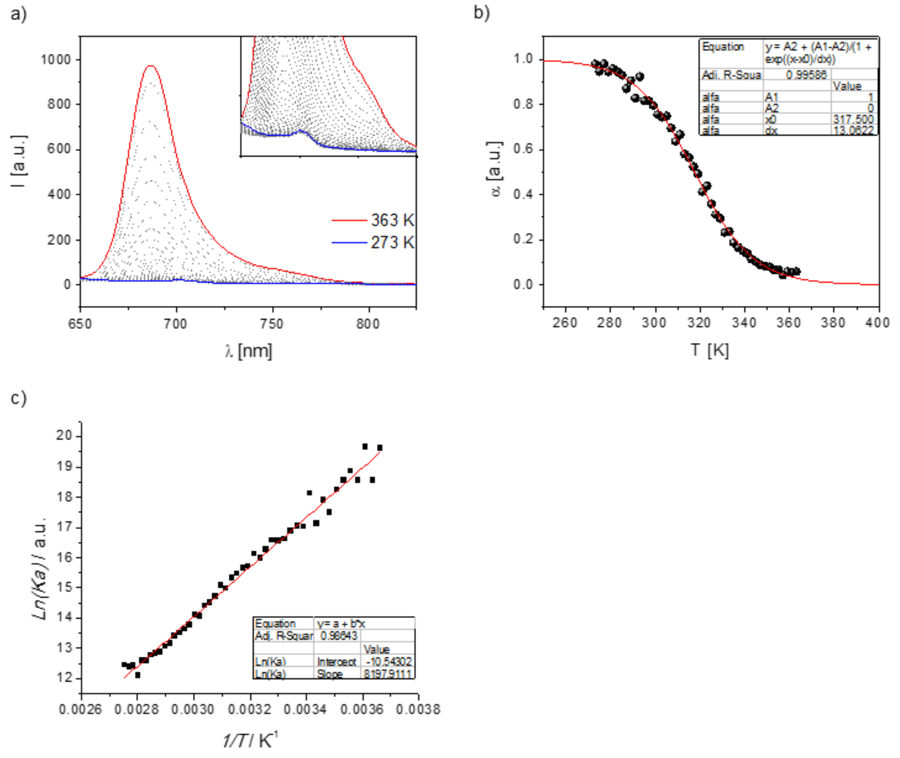

Figure S9. Temperature-dependent fluorescence spectra (b) of $\mathbf{1}_{\mathbf{aggB}}$ in DMSO/water (5:95) at 5.0 x 10^{-7} M from 363 (red) to 293 K (blue). Fluorescence recorded at λ_{exc} = 615 nm. Data recorded every 2 K, applying a temperature gradient of -2 K/min. b) Fraction of aggregated species *versus* temperature and fitting of the spectral changes to the Boltzmann equation (R^2 = 0.9995). c) Van't Hoff plot for **1** in the aforementioned conditions.

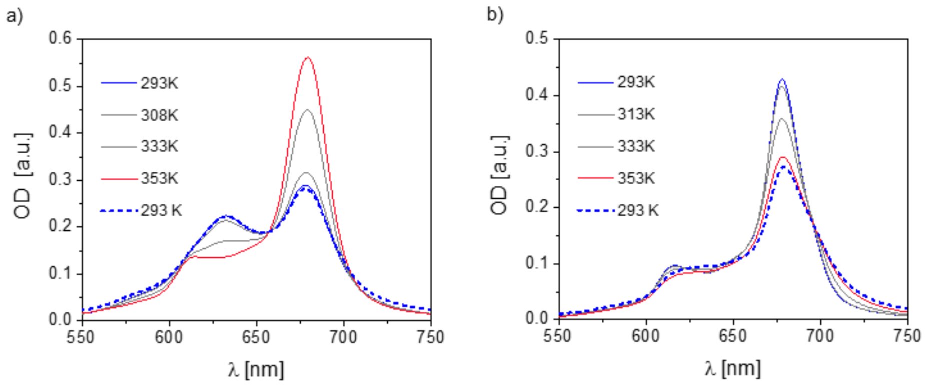

Figure S10. Temperature-dependent absorption spectra of $\mathbf{1}_{\mathbf{aggA}}$ (a) and $\mathbf{1}_{\mathbf{aggB}}$ (b) mixed with **4** (2:1 ratio) in DMSO/water (40:60 and 5:95, respectively) at 2.0 x 10^{-5} M from 293 to 353 K.

ZnPc-C_{60} titrations

Monomeric state

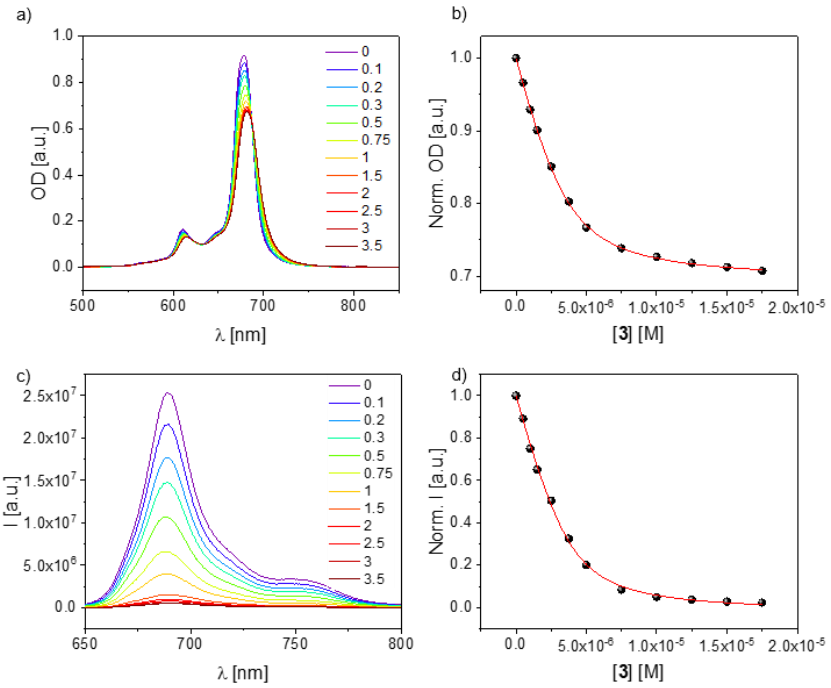

Figure S11. Absorption (a) and fluorescence (c) spectra of **1** (5.0 x 10^{-6} M) upon addition of **3** from 0 (purple) up to 1.75 x 10^{-5} M (red) in DMSO. Fluorescence recorded at λ_{exc} = 615 nm. Absorption of the added fullerenes were subtracted from the corresponding spectra. Plot of normalized change in absorption at 678 (b) and fluorescence at 689 nm (d) *versus* the concentration of **3**. Solid red line shows the curve-fit of R^2 = 0.999 and R^2 = 0.990, respectively, obtained by nonlinear least-squares analysis.

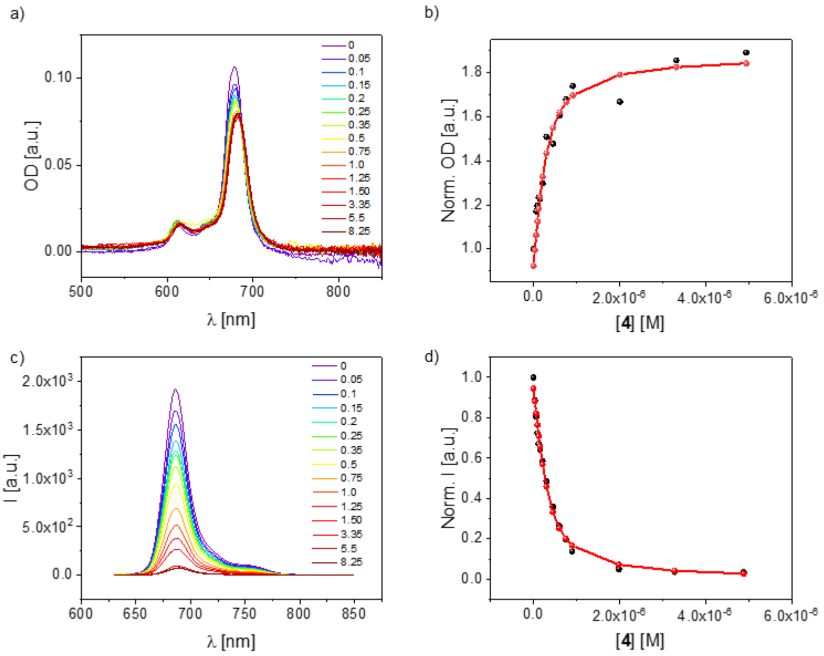

Figure S12. Absorption (a) and fluorescence (c) spectra of **1** (6 x 10^{-7} M) upon addition of **4** from 0 (purple) up to 5 x 10^{-6} M (red) in DMSO. Fluorescence recorded at λ_{exc} = 615 nm. Absorption of the added fullerenes were subtracted from the corresponding spectra. Plot of normalized change in absorption at 700 (b) and fluorescence at 684 nm (d) *versus* the concentration of **4**. Solid red line/dots shows the curve-fit of R^2 = 0.999 and R^2 = 0.999, respectively, obtained by nonlinear least-squares analysis.

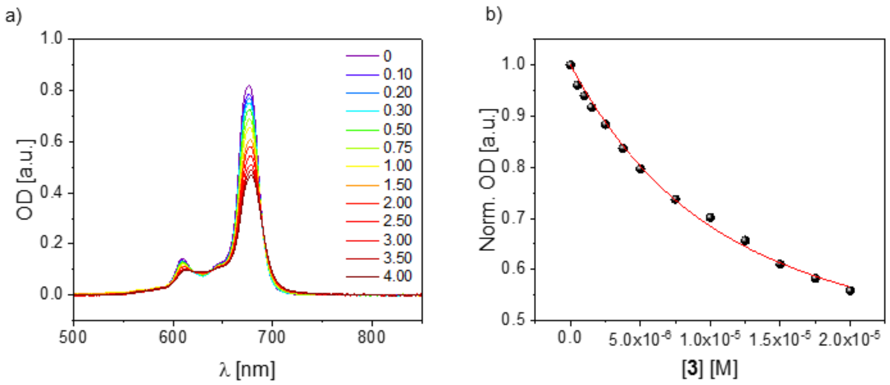

Figure S13. Absorption spectra (a) of **2** (5.0×10^{-6} M) upon addition of **3** from 0 (purple) up to 1.75×10^{-5} M (red) in DMSO. Absorption of the added fullerenes were subtracted from the corresponding spectra. Plot of normalized change in absorption (b) at 678 nm *versus* the concentration of **3**.

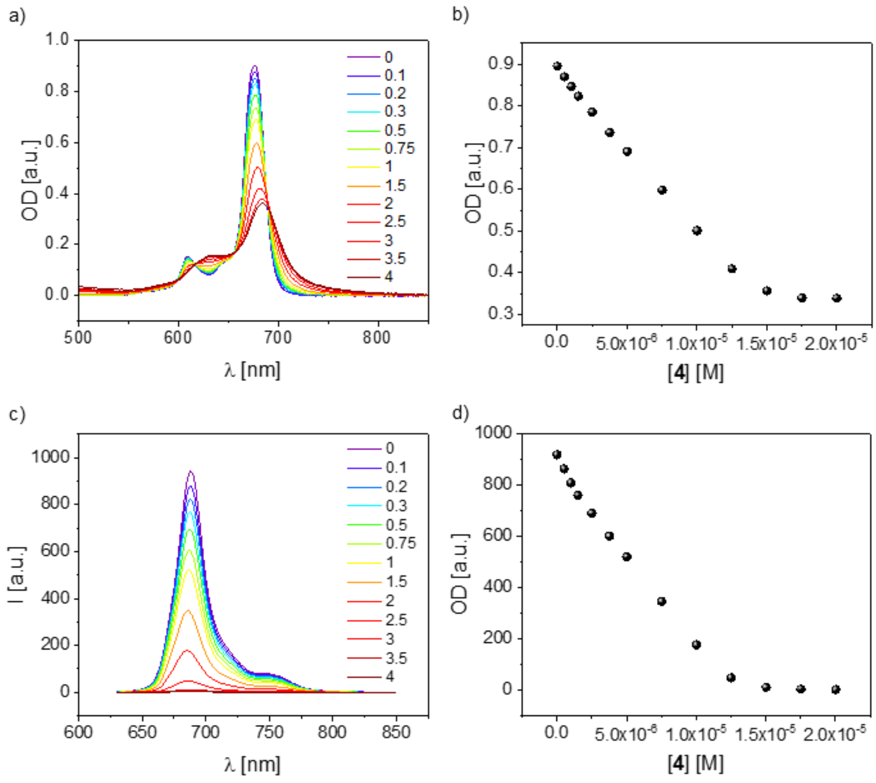

Figure S14. Absorption (a) and fluorescence (c) spectra of **2** (5.0×10^{-6} M) upon addition of **4** from 0 (purple) up to 2×10^{-5} M (red) in DMSO. Fluorescence recorded at λ_{exc} = 615 nm. Absorption of the added fullerenes were subtracted from the corresponding spectra. Plot of the change in absorption at 678 nm (b) and fluorescence at 690 nm (d) *versus* the concentration of **4**.

Aggregated state

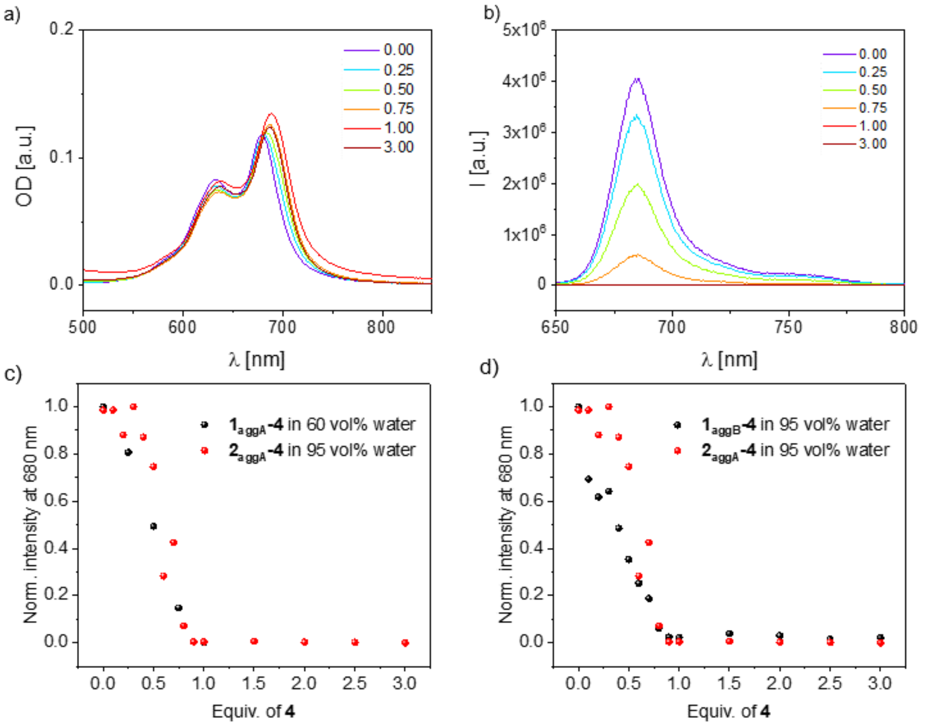

Figure S15. (a,b) Absorption (a) and fluorescence (b) spectra of $\mathbf{1}_{\mathbf{aggA}}$ (2.0 x 10^{-6} M) upon addition of **4** from 0 (purple) up to 6 x 10^{-6} M (red) in DMSO/water (40:60). Absorption of the added fullerenes were subtracted from the corresponding spectra. (c,d) Fluorescence intensity at 680 nm of c) $\mathbf{1}_{\mathbf{aggA}}$ and $\mathbf{2}_{\mathbf{aggA}}$ and d) $\mathbf{1}_{\mathbf{aggB}}$ and $\mathbf{2}_{\mathbf{aggA}}$ (2.0 x 10^{-6} M) upon addition of up to three equivalents of **4**. Fluorescence recorded at λ_{exc} = 615 nm.

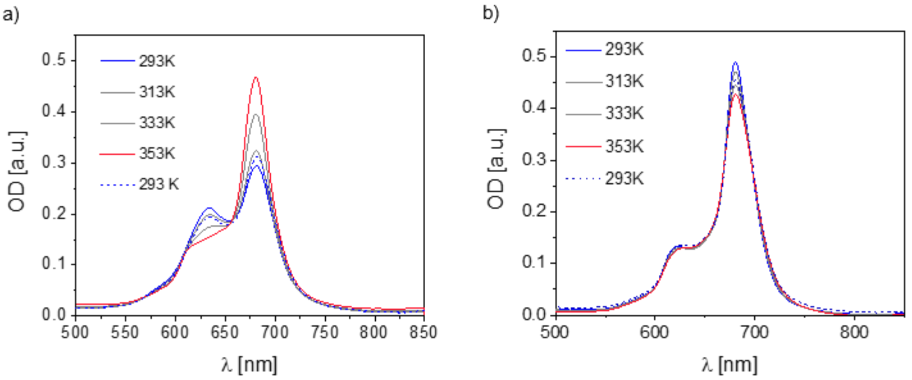

Figure S16. Temperature-dependent absorption of $\mathbf{1}_{\mathbf{aggA}}$ (a) and $\mathbf{1}_{\mathbf{aggB}}$ (b) mixed with **4** (2:1 ratio) in DMSO/water (40:60 and 5:95, respectively) at 2.0 x 10^{-5} M from 293 to 353 K.

Transmission electron microscopy

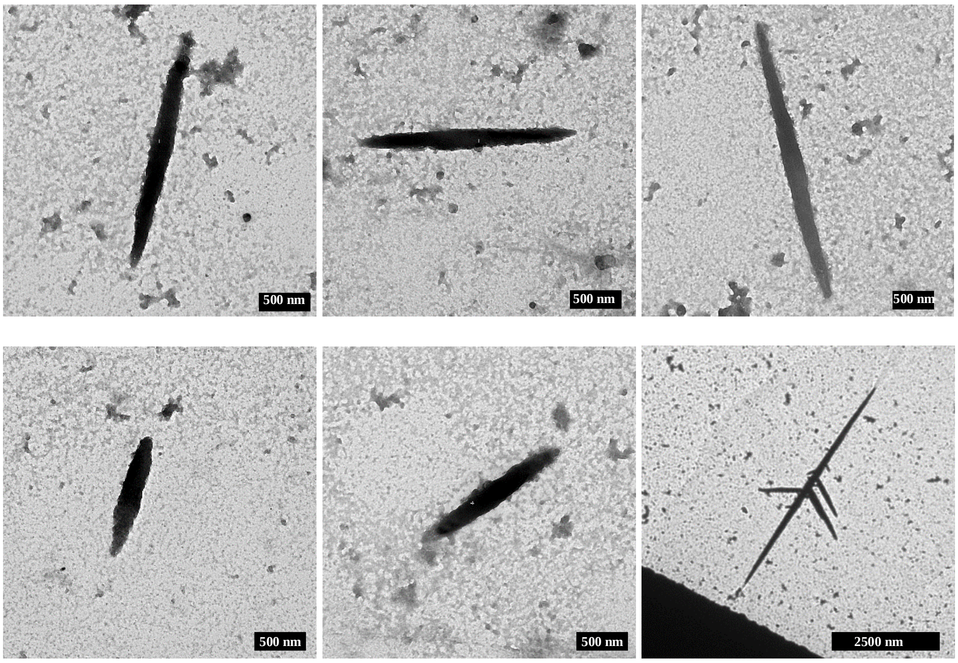

Figure S17. TEM image of $\mathbf{1}_{aggB}$ in 95 vol% water deposited onto a carbon-coated copper grid with an acceleration voltage of 80 kV. Samples were prepared from a solution of **1** (2.0 x 10^{-5} M) in DMSO, which was dispersed into water.

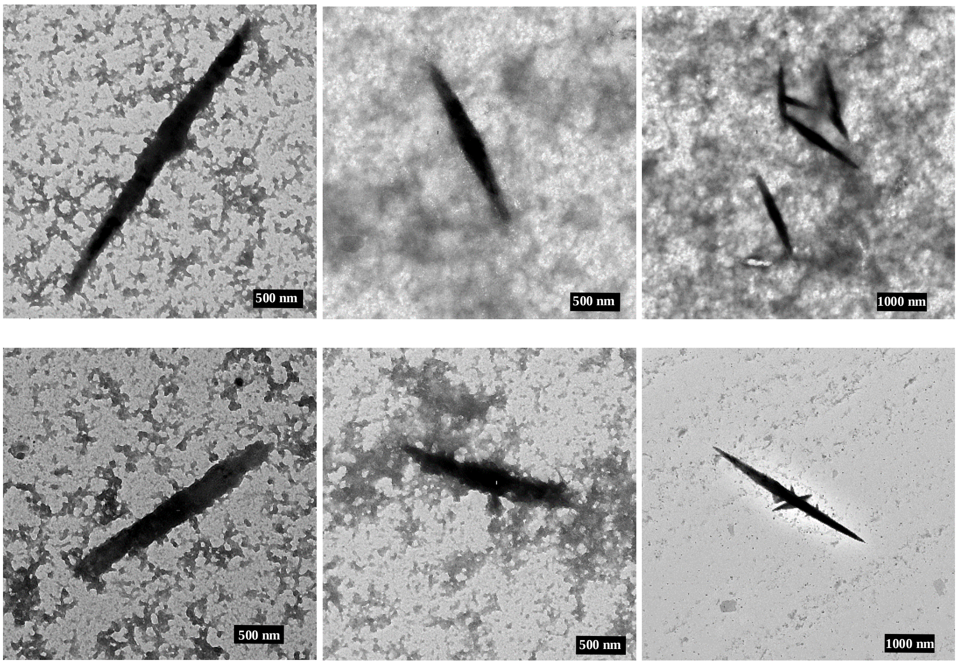

Figure S18. TEM image of $\mathbf{1}_{aggB}$ in presence of **4** (1:2 ratio) in 95 vol% water deposited onto a carbon-coated copper grid with an acceleration voltage of 80 kV. Samples were prepared from a mixed solution of **1** (2.0 x 10^{-5} M) and **4** (4.0 x 10^{-5} M) in DMSO, which was dispersed into water.

Molecular Modelling

Two general approaches were used to study the monomeric **1** and **2** as well as the resulting aggregates. Dreiding forcefield calculations[5] utilizing Forcite Forcefield Calculation Package included in Materials Studio software 2018[6] were used for preoptimization, conformer screening, simulated annealing and molecular dynamics. For this purpose, the Dreiding Zn parameter were parametrized against wB97xd/def2-SVP DFT - optimized structures of **1** to reproduce the Zn-N bond distance (Zn-N ≈ 1.9 Å) and angles (∢(N_1-Zn-N_3) ≈ 180°, ∢(N_1-Zn-N_2) ≈ 90°). All other parameters, including the parameters for the (12,6)-Lennard Jones potential were kept from the original Dreiding Forcefield.
First the Monomer of **1** and **2** were optimized, followed by a conformation scan including 10000 conformers of a unique regioisomer, varying all dihedral angles of the single bonds at once, followed by an optimization of all conformers. A series of 5 monomers, including the energetic most favourable conformations with significant difference in structure, was chosen to be then further optimized with DFT in Gaussian 16.[7] Optimization was performed with dispersion and long range corrected wB97xd functional, including PCM solvation of water.[8-14] Basis set for optimization and single point calculation was def2-SVP. To elucidate the size of the basis set single point calculations were also performed on the wB97xd/def2-TZVP//wB97xd/def2-SVP and wB97xd/def2-TZVPPD//wB97xd/def2-SVP level of theory (Figure S21 and Table S3).[15-16]
Dimers of **1** were constructed under the estimation, that the preferable monomer geometry stays intact. In total 12 input structures were generated featuring four pyrene-pyrene-, four pyrene-ZnPc-, and four ZnPc-ZnPc-interactions, respectively. Dreiding forcefield optimizations, followed by 1000 simulated annealing cycles and reoptimization of each structure was performed. The best stabilized dimer was then used as starting geometry to restart this cycle a second and a third time. The energetically best four conformers were chosen to study on the wB97xd/def2-SVP level of theory. Optimization was performed with water PCM solvation and single point calculations including counterpoise correction were performed in gas phase, toluene and DMSO, as the basis set is incomplete. Thus, the interaction energies, basis set superposition errors, deformation energy of the monomer and total dimerization energies could be estimated according to the following equations:

$$E_i = E_{AB}^{AB}(AB) - E_{A}^{AB}(AB) - E_{B}^{AB}(AB)$$

With E_i being the interaction energy and the subscript AB defines the structure used for the single point calculation. In case of AB it corresponds to the dimer, build-up of monomers A and B. The superscript indicates the basis set used for the single point calculation (here all basis functions of the dimer AB) and in brackets the origin of the optimized structure used for the single point calculation (here always the dimer AB). E_{BSSE} corresponds to the basis set superposition error, E_{def} to the deformation energy and E_D is the dimerization energy.

$$E_{BSSE} = E_{A}^{AB}(AB) - E_{A}^{A}(AB)$$
$$E_{def}(A) = E_{A}^{A}(AB) - E_{A}^{A}(A)\, , E_{def}(B) = E_{B}^{B}(AB) - E_{B}^{B}(B)$$
$$E_D = E_i + E_{def}(A) + E_{def}(B)$$

Next, the first 12 vertical excitations involving singlet states were studied for the dimer and monomer. TD-DFT with the same method and basis was used. DMSO was chosen as PCM solvent, as this offers better comparability of the monomer to experimental data.
The same search algorithm of Dreiding optimization and simulated annealing was performed to find dimers and trimers of **1** with **3** and **4**. Similar, DFT optimizations were performed – however we exchanged the basis set to 3-21G[17-18] for the pre-opzimizations and Lan2DZ (Lan2DZdp for phosphorus)[19-25] for the final iterations – as they showed faster SCF convergence than def2-SVP on this larger complexes.
The information of the dimer orientation and binding geometry was then used to form larger aggregates of **1** with Dreiding optimizations and simulated annealing.

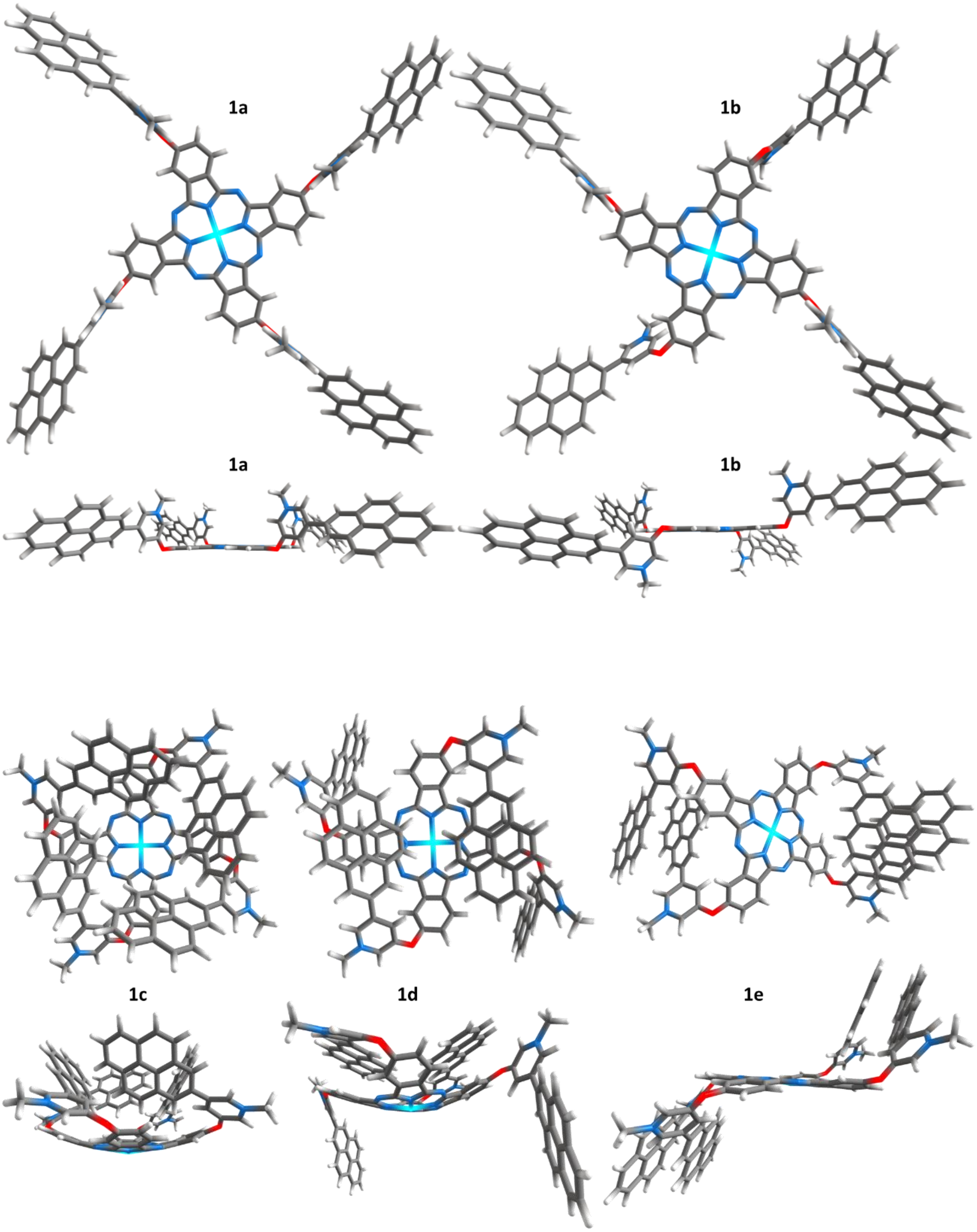

Figure S19. wB97xd/def2-SVP optimized geometries of different conformations of **1**. Geometries **1c-d** were predicted to be energetically most favorable during the Dreiding forcefield pre-screening.

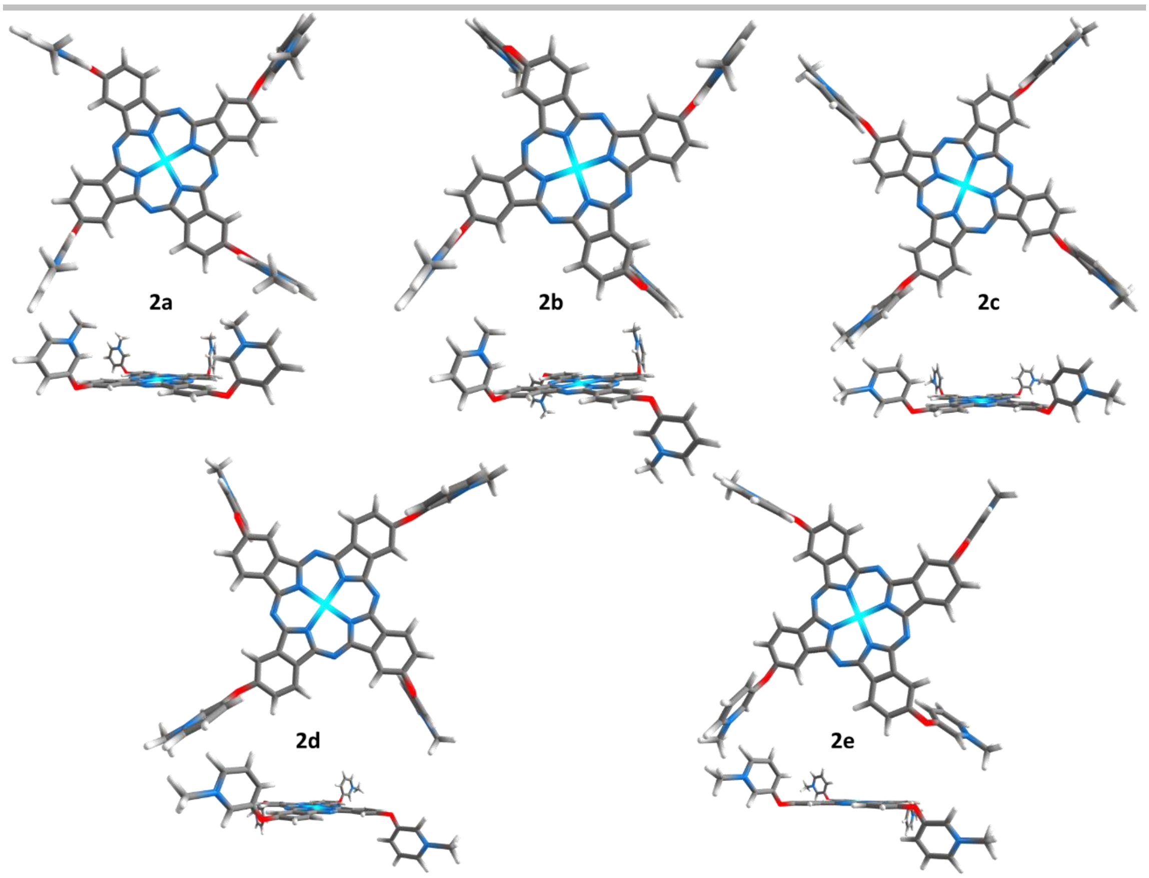

Figure S20. wB97xd/def2-SVP optimized geometries of different conformations of **2**.

Table S2. Absolute and relative energies of the different monomers predicted with wB97xd/def2-SVP using PCM. solvation of toluene, DMSO and water.

	PCM solvent	water		DMSO		toluene	
		E Ha	ΔE kcal mol^{-1}	E Ha	ΔE kcal mol^{-1}	E Ha	ΔE kcal mol^{-1}
monomer	2a	-4893.661515	0.44	-4891.399054	0.77	-4891.187598	8.10
	2b	-4893.661986	0.15	-4891.399748	0.34	-4891.194363	3.86
	2c	-4893.661376	0.53	-4891.399396	0.56	-4891.197612	1.82
	2d	-4893.661513	0.44	-4891.399645	0.40	-4891.200513	0.00
	2e	-4893.662222	0.00	-4891.400288	0.00	-4891.198875	1.03
monomer	1a	-7347.164949	37.77	-7347.159851	37.90	-7346.95869	42.23
	1b	-7347.165012	37.73	-7347.160121	37.73	-7346.962924	39.57
	1c	-7347.220280	3.08	-7347.215222	3.19	-7347.017744	5.20
	1d	-7347.193278	20.01	-7347.188006	20.25	-7346.980291	28.68
	1e	-7347.225185	0.00	-7347.220304	0.00	-7347.026035	0.00

SUPPORTING INFORMATION

Table S3. Absolute and relative energies predicted with wB97xd/def2-SVP, wB97xd/def2-TZVP and wB97xd/def2-TZVPPD using PCM solvation of water.

		E / Ha	E / Ha	E / Ha	ΔE / kcal mol^{-1}	ΔE / kcal mol^{-1}	ΔE / kcal mol^{-1}
		def2-SVP	def2-TZVP	def2-TZVPPD	def2-SVP	def2-TZVP	def2-TZVPPD
		water	water	water	water	water	water
monomer	2a	-4893.661515	-4894.947615	-	0.44283129	0.336940715	-
	2b	-4893.661986	-4894.948076	-	0.1479093	0.04762719	-
	2c	-4893.661376	-4894.947204	-	0.5302539	0.594562475	-
	2d	-4893.661513	-4894.947327	-	0.4441668	0.517259645	-
	2e	-4893.662222	-4894.948152	-	0	0	-
monomer	1a	-7347.164949	-7353.228329	-7353.275899	37.7676585	31.34273307	31.51750333
	1b	-7347.165012	-7353.228429	-7353.275999	37.7279694	31.27968822	31.45465678
	1c	-7347.22028	-7353.273871	-7353.321195	3.0753096	2.78756676	3.11672238
	1d	-7347.193278	-7353.245726	-7353.293574	20.0052501	20.43437517	20.43474261
	1e	-7347.225185	-7353.278317	-7353.326166	0	0	0

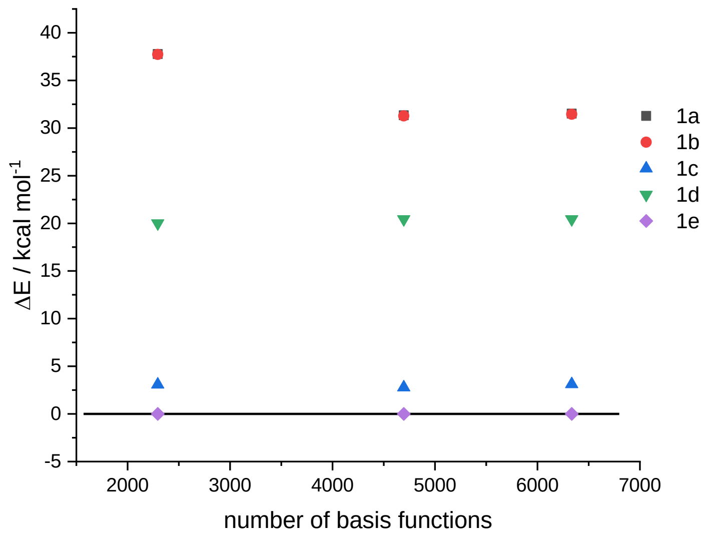

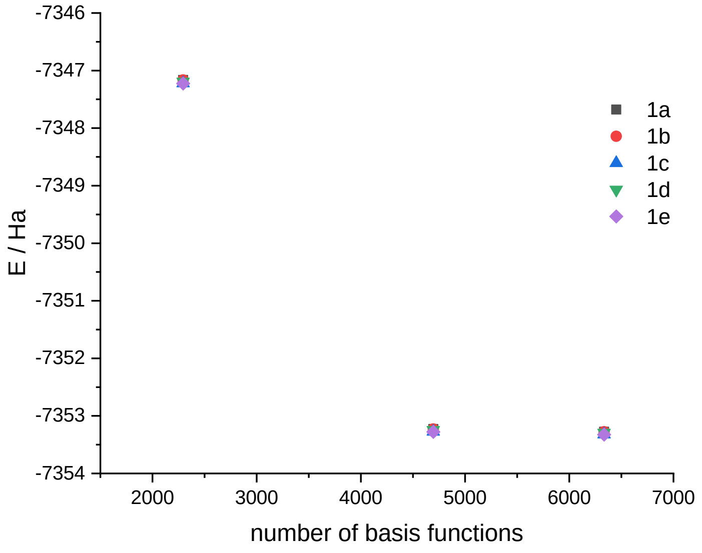

Figure S21. Relative and absolute energy predicted with wB97xd with def2-SVP (2295 basis functions) def2-TZVP (4696 basis functions) and def2-TZVPPD (6335 basis functions).

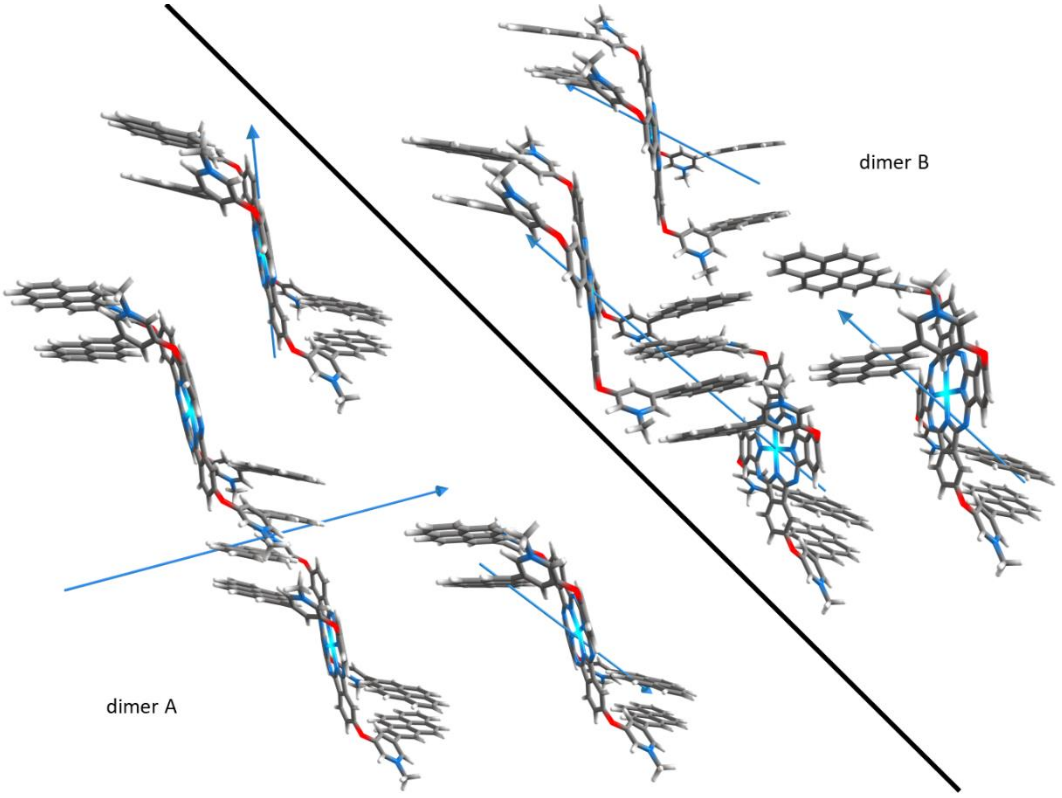

Figure S22. wB97xd/def2-SVP optimized structures and dipole moment of dimer A (bottom left) and dimer B (top right) as well as the monomers in the same orientation. Total dipole moments are 195.3 Debye (dimer A), 481.9 Debye (dimer B) and 31.2 Debye (optimized monomer).

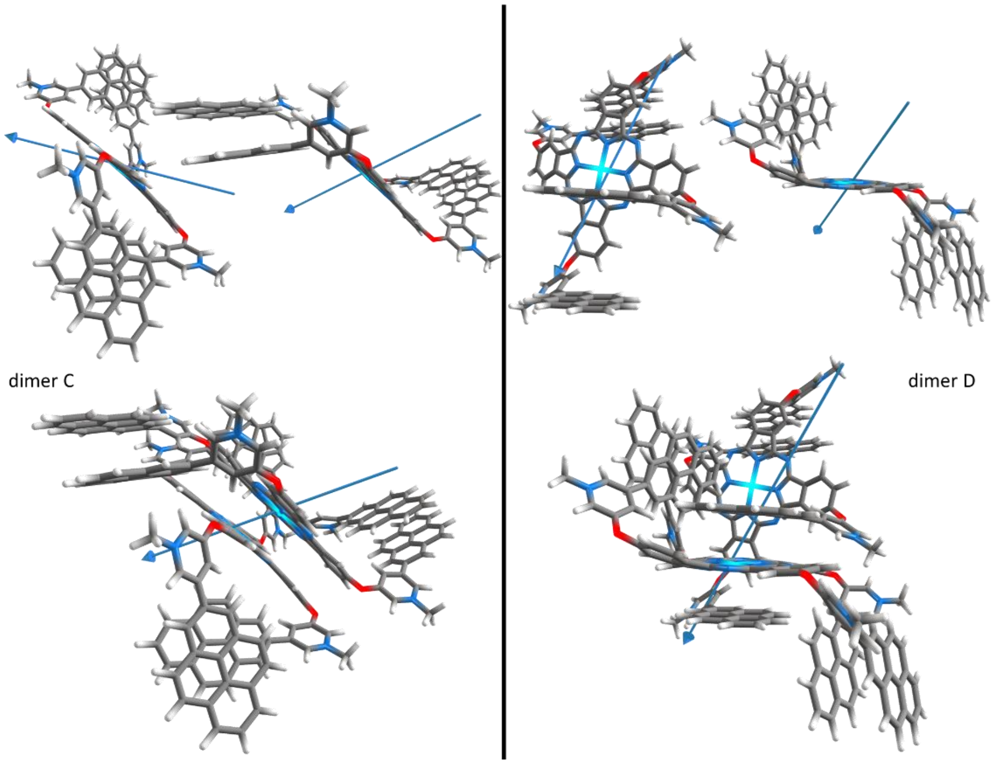

Figure S23. wB97xd/def2-SVP optimized structures and dipole moment of dimer C (left) and dimer D (right) as well as the monomers in the same orientation. Total dipole moments are 126.7 Debye (dimer C), 485.1 Debye (dimer D) and 31.2669 Debye (optimized monomer).

Table S4. Energies for charged dimers A-D derived with wB97xd/def2-SVP in gas phase and PCM solvation of toluene, DMSO and water. Energy differences ΔE correspond to interaction energy (ΔE_i - orange), the basis set superposition error (ΔE_{BSSE} - green) and the deformation energy per monomer (ΔE_{def} - yellow).

	molecule (basis set)	dimer A			dimer B			dimer C			dimer D		
		E	ΔE	ΔE	E	ΔE	ΔE	E	ΔE	ΔE	E	ΔE	ΔE
		Ha	Ha	kcal mol^{-1}	Ha	Ha	kcal mol^{-1}	Ha	Ha	kcal mol^{-1}	Ha	Ha	kcal mol^{-1}
gas phase	AB(AB)	-14693.048109	0.393386	246.85	-14693.045074	0.348804	218.87	-14692.925037	0.527112	330.76	-14693.045074	0.383398	240.58
	A(AB)	-7346.721240	-0.002978	-1.87	-7346.700910	-0.008729	-5.48	-7346.726420	-0.012513	-7.85	-7346.698960	-0.013134	-8.24
	B(AB)	-7346.720255	-0.002996	-1.88	-7346.692968	-0.008608	-5.40	-7346.725728	-0.012504	-7.85	-7346.729513	-0.016086	-10.09
	A(A)	-7346.718263	0.000185	0.12	-7346.692180	0.026267	16.48	-7346.713907	0.004541	2.85	-7346.685826	0.032622	20.47
	B(B)	-7346.717259	0.001189	0.75	-7346.684361	0.034087	21.39	-7346.713224	0.005224	3.28	-7346.713427	0.005021	3.15
toluene	AB(AB)	-14693.962401	0.100291	62.93	-14693.964562	0.044789	28.11	-14693.968433	0.097852	61.40	-14693.964562	0.094345	59.20
	A(AB)	-7347.030715	-0.005954	-3.74	-7347.003273	-0.012394	-7.78	-7347.033950	-0.021385	-13.42	-7347.012058	-0.025536	-16.02
	B(AB)	-7347.031978	-0.008989	-5.64	-7347.006078	-0.013025	-8.17	-7347.032336	-0.020552	-12.90	-7347.046849	-0.030282	-19.00
	A(A)	-7347.024761	0.001274	0.80	-7346.990880	0.035156	22.06	-7347.012565	0.013470	8.45	-7346.986522	0.039513	24.79
	B(B)	-7347.022989	0.003046	1.91	-7346.993053	0.032982	20.70	-7347.011783	0.014252	8.94	-7347.016567	0.009468	5.94
DMSO	AB(AB)	-14694.473783	-0.031833	-19.98	-14694.478242	-0.090412	-56.73	-14694.530878	-0.099147	-62.21	-14694.541597	-0.128224	-80.46
	A(AB)	-7347.221345	-0.001765	-1.11	-7347.193142	-0.004961	-3.11	-7347.215576	-0.008710	-5.47	-7347.190801	-0.007345	-4.61
	B(AB)	-7347.220604	-0.001906	-1.20	-7347.194688	-0.005840	-3.66	-7347.216155	-0.009284	-5.83	-7347.222572	-0.010886	-6.83
	A(A)	-7347.219580	0.000724	0.45	-7347.188181	0.032124	20.16	-7347.206867	0.013437	8.43	-7347.183456	0.036848	23.12
	B(B)	-7347.218698	0.001607	1.01	-7347.188848	0.031456	19.74	-7347.206871	0.013434	8.43	-7347.211685	0.008619	5.41
water	AB(AB)	-14694.487212	-0.035261	-22.13	-14694.491642	-0.093860	-58.90	-14694.545433	-0.103857	-65.17	-14694.557441	-0.133038	-83.48
	A(AB)	-7347.226341	-0.001889	-1.19	-7347.198174	-0.005047	-3.17	-7347.220444	-0.008677	-5.44	-7347.196879	-0.008533	-5.35
	B(AB)	-7347.225610	-0.002026	-1.27	-7347.199607	-0.005888	-3.69	-7347.221131	-0.009375	-5.88	-7347.227524	-0.010989	-6.90
	A(A)	-7347.224452	0.000733	0.46	-7347.193127	0.032057	20.12	-7347.211768	0.013417	8.42	-7347.188346	0.036838	23.12
	B(B)	-7347.223585	0.001600	1.00	-7347.193719	0.031466	19.74	-7347.211757	0.013428	8.43	-7347.216535	0.008650	5.43

Table S5. Energies for uncharged dimers A-D (ZnPc 7 as monomeric building block) derived with wB97xd/def2-SVP in gas phase and PCM solvation of toluene, DMSO and water. Energy differences ΔE correspond to interaction energy (ΔE_i - orange), the basis set superposition error (ΔE_{BSSE} - green) and the deformation energy per monomer (ΔE_{def} - yellow).

	molecule (basis set)	dimer			dimer inter			dimer PC			dimer Pcpyr		
		E	ΔE	ΔE	E	ΔE	ΔE	E	ΔE	ΔE	E	ΔE	ΔE
		Ha	Ha	kcal mol^{-1}	Ha	Ha	kcal mol^{-1}	Ha	Ha	kcal mol^{-1}	Ha	Ha	kcal mol^{-1}
GP	AB(AB)	-14376.623519	-0.036133	-22.66	-14376.631509	-0.096342	-60.41	-14376.692320	-0.103819	-65.09	-14376.709080	-0.140946	-88.37
	A(AB)	-7188.294169	-0.003369	-2.11	-7188.266966	-0.009166	-5.75	-7188.294025	-0.013576	-8.51	-7188.265619	-0.013861	-8.69
	B(AB)	-7188.293217	-0.003310	-2.08	-7188.268201	-0.009171	-5.75	-7188.294476	-0.013533	-8.49	-7188.302515	-0.017362	-10.89
	A(A)	-7188.290799	-0.003016	-1.89	-7188.257800	0.029983	18.80	-7188.280449	0.007334	4.60	-7188.251758	0.036024	22.59
	B(B)	-7188.289907	-0.002124	-1.33	-7188.259030	0.028752	18.03	-7188.280944	0.006839	4.29	-7188.285153	0.002630	1.65
tol	AB(AB)	-14376.664249	-0.035487	-22.25	-14376.672429	-0.095005	-59.57	-14376.730828	-0.103647	-64.99	-14376.746400	-0.139264	-87.32
	A(AB)	-7188.314768	-0.002818	-1.77	-7188.288379	-0.007767	-4.87	-7188.313208	-0.011841	-7.42	-7188.286171	-0.011449	-7.18
	B(AB)	-7188.313994	-0.002768	-1.74	-7188.289045	-0.007856	-4.93	-7188.313974	-0.012015	-7.53	-7188.320966	-0.014814	-9.29
	A(A)	-7188.311950	-0.003351	-2.10	-7188.280612	0.027987	17.55	-7188.301367	0.007233	4.53	-7188.274722	0.033878	21.24
	B(B)	-7188.311227	-0.002628	-1.65	-7188.281189	0.027411	17.19	-7188.301958	0.006641	4.16	-7188.306152	0.002447	1.53
DMSO	AB(AB)	-14376.718244	-0.034536	-21.65	-14376.726536	-0.092620	-58.07	-14376.787242	-0.104367	-65.44	-14376.797750	-0.135823	-85.16
	A(AB)	-7188.342195	-0.002141	-1.34	-7188.316598	-0.005768	-3.62	-7188.341001	-0.011133	-6.98	-7188.314833	-0.009561	-5.99
	B(AB)	-7188.341514	-0.002091	-1.31	-7188.317318	-0.006091	-3.82	-7188.341875	-0.011325	-7.10	-7188.347093	-0.013204	-8.28
	A(A)	-7188.340054	-0.004107	-2.57	-7188.310830	0.025117	15.75	-7188.329868	0.006079	3.81	-7188.305272	0.030675	19.23
	B(B)	-7188.339423	-0.003475	-2.18	-7188.311227	0.024720	15.50	-7188.330550	0.005397	3.38	-7188.333889	0.002058	1.29
water	AB(AB)	-14376.719961	-0.034492	-21.63	-14376.728280	-0.092515	-58.01	-14376.789305	-0.104478	-65.51	-14376.799547	-0.135595	-85.02
	A(AB)	-7188.343076	-0.002128	-1.33	-7188.317500	-0.005714	-3.58	-7188.341947	-0.011141	-6.99	-7188.315830	-0.009587	-6.01
	B(AB)	-7188.342393	-0.002077	-1.30	-7188.318264	-0.006040	-3.79	-7188.342880	-0.011401	-7.15	-7188.348122	-0.013362	-8.38
	A(A)	-7188.340948	-0.004136	-2.59	-7188.311786	0.025026	15.69	-7188.330806	0.006006	3.77	-7188.306243	0.030570	19.17
	B(B)	-7188.340316	-0.003504	-2.20	-7188.312224	0.024588	15.42	-7188.331480	0.005333	3.34	-7188.334760	0.002052	1.29

Table S6. Total dimerization energies (E_D) for dimer A-D (top) and for neutral dimers formed by ZnPc 7 in the same orientation (middle) calculated with wB97xd/def2-SVP in various PCM solvation environments. Bottom: Estimation of the coulomb repulsion $E_{Coul} = E_D(charged) - E_D(uncharged)$

	ε_r	E_D kcal mol^{-1}			
		dimer A	dimer B	dimer C	dimer D
gasphase	1.00	247.71	256.75	336.89	264.20
toluene	2.37	65.64	70.86	78.80	89.94
DMSO	46.83	-18.51	-16.84	-45.35	-51.93
water	78.35	-20.66	-19.04	-48.33	-54.94

	ε_r	E_D kcal mol^{-1} uncharged dimer			
		dimer A	dimer B	dimer C	dimer D
gasphase	1.00	-25.88	-23.58	-56.21	-64.14
toluene	2.37	-26.00	-24.83	-56.29	-64.54
DMSO	46.83	-26.41	-26.82	-58.24	-64.64
water	78.35	-26.42	-26.90	-58.40	-64.56

	ε_r	$E_{Coulomb}$ kcal mol^{-1}			
		dimer A	dimer B	dimer C	dimer D
gasphase	1.00	273.59	280.33	393.10	328.34
toluene	2.37	91.64	95.70	135.09	154.48
DMSO	46.83	7.90	9.99	12.89	12.71
water	78.35	5.75	7.86	10.07	9.63

To extrapolate the dimerization energies towards the energy gain per monomer in the aggregates, we applied a linear superposition model. This is useful, as the number of interaction motifs differs for the aggregates. Please note, that this is only a coarse guess for multiple reasons: First, only the first layer of aggregation was taken in account. Second – no change in electronic structure or reorganization is included, as the energies are extrapolated from the dimer calculations. We choose to separate the attractive Van der Waals interactions from the repulsive Coulomb part, to account for the short- and longer-range character, respectively (typically $\sim r^{-6}$ vs. $\sim r^{-1}$). The number of π-interactions can be directly extrapolated from the dimer structure. For the Coulomb part we counted all pyridinium cations within a Cutoff of 10 Å next to the monomer pyridinium moieties.

Table S7. Extrapolated estimation of the energy gain per monomer in aggregates (Emon), based on dimer A-D.

	dimer A	dimer B	dimer C	dimer D
π-π-interactions in aggregates per monomer	4	8	2	4
π-π-interactions in the dimer	1	3	1	2
Coulomb close contacts in aggregate (10 Å cutoff-distance)	20	16	16	20
Coulomb interaction in dimers	4	4	4	4

	ε_r	E_{mon} kcal mol^{-1}			
		dimer A	dimer B	dimer C	dimer D
gasphase	1.00	1264	1058	1460	1513
toluene	2.37	354	317	428	643
DMSO	46.83	-66	-32	-65	-66
water	78.35	-77	-40	-77	-81

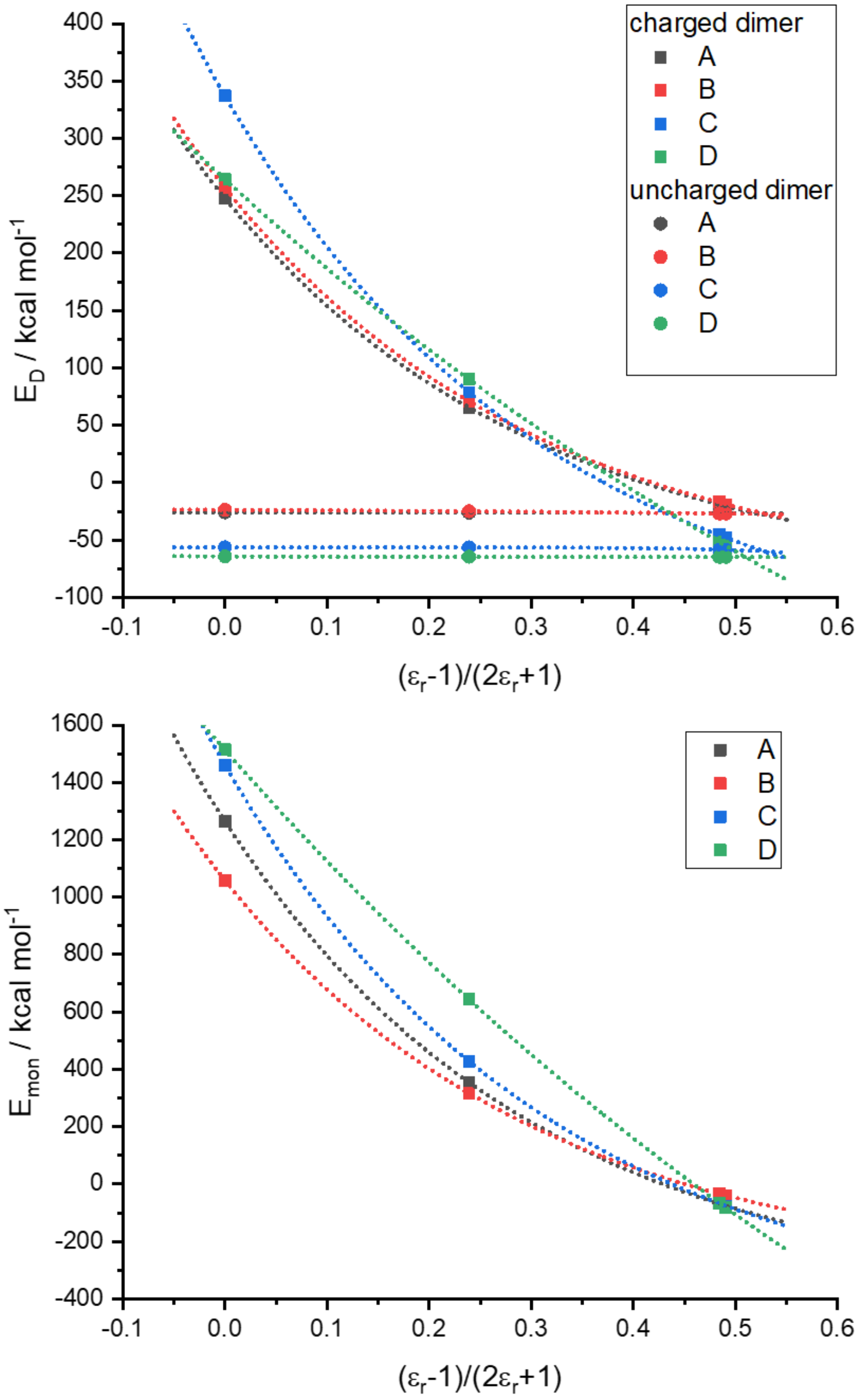

Figure S24. wB97xd/def2-SVP predicted dimerization energy (E_D, top) and possible interaction energy in the aggregates per monomer (E_{mon}), assuming equal interaction energy per binding motif and usage of all possible interaction motifs (bottom) as function of the Kirkwood solvent parameter, fitted with an monoexponential decay.

Table S8. TD-DFT wB97xd/def2-SVP derived energy for the first vertical transitions for the monomer and dimer A-D in eV and the corresponding oscillator strength (OSC).

	monomer		dimer							
			A		B		C		D	
interaction motive			pyrene π-interaction		pyrene π-interaction intercalating		ZnPc H-type		ZnPc - pyrene π-interaction	
State	eV	OSC	eV	OSC	eV	OSC	eV	OSC	eV	OSC
S1	1.896	0.7834	1.893	1.5641	1.908	1.4074	1.784	0.0045	1.829	0.4037
S2	1.921	0.6353	1.903	0.2133	1.914	0.1381	1.794	0.0053	1.847	0.2597
S3	3.411	0.0000	1.924	0.4384	1.925	0.4348	1.952	0.9224	1.915	0.6489
S4	3.428	0.0000	1.928	0.6731	1.935	0.7460	1.989	1.0903	1.921	0.7267
S5	3.748	0.0165	3.473	0.0001	3.372	0.0010	2.116	0.0228	2.862	0.0069
S6	3.754	0.0109	3.490	0.0001	3.389	0.0004	2.150	0.0275	2.882	0.0088
S7	3.755	0.0007	3.503	0.0015	3.473	0.0003	2.164	0.0712	2.909	0.0190
S8	3.758	0.0100	3.524	0.0042	3.488	0.0000	2.199	0.2248	2.954	0.0301
S9	3.773	0.0154	3.617	0.0007	3.491	0.0002	3.462	0.0084	3.089	0.0000
S10	3.776	0.0136	3.676	0.0026	3.497	0.0000	3.470	0.0080	3.116	0.0001
S11	3.796	0.0000	3.703	0.0016	3.627	0.0001	3.473	0.0006	3.323	0.0000
S12	3.837	0.0076	3.711	0.0380	3.673	0.0095	3.483	0.0024	3.352	0.0000

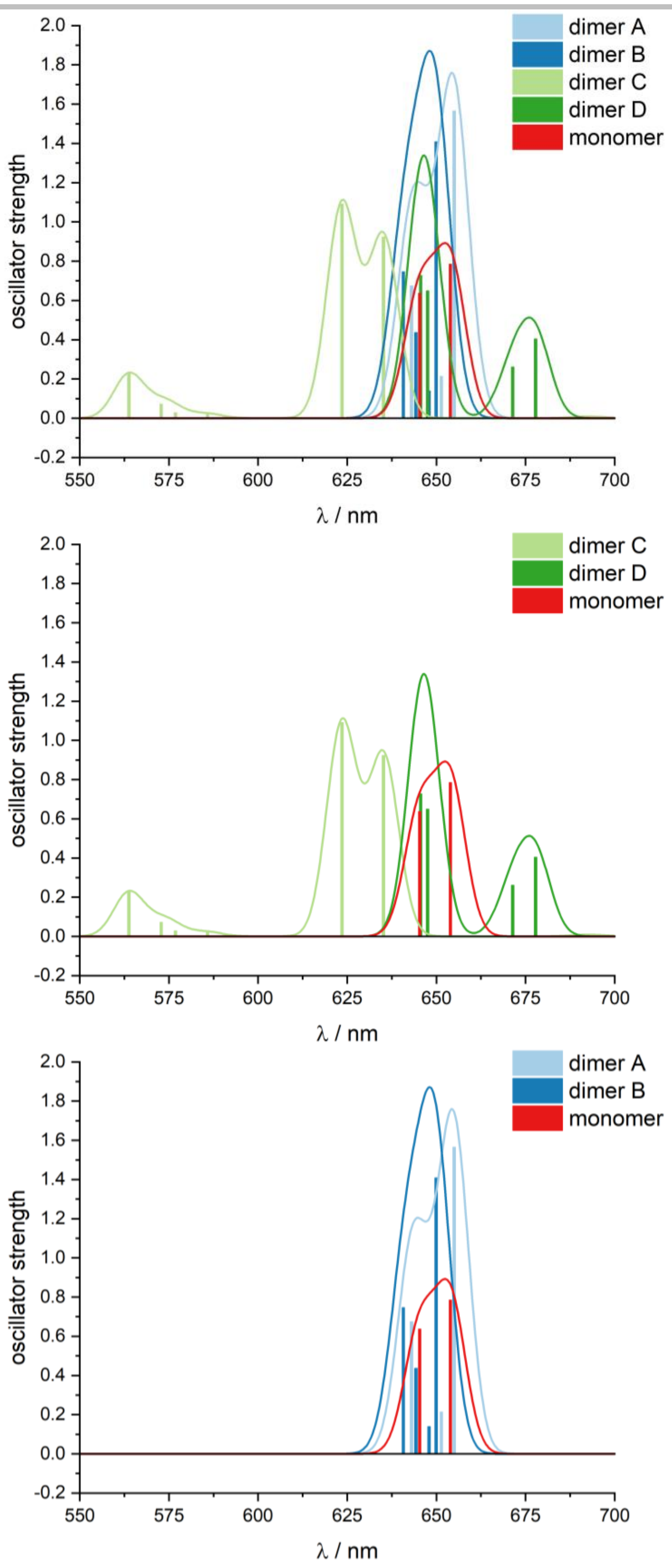

Figure S25. Vertical transitions calculated on the TD-DFT wB97xd/def2-SVP level of theory including water PCM solvation. Gaussian distribution with 10nm FWHM used to generate the spectra.

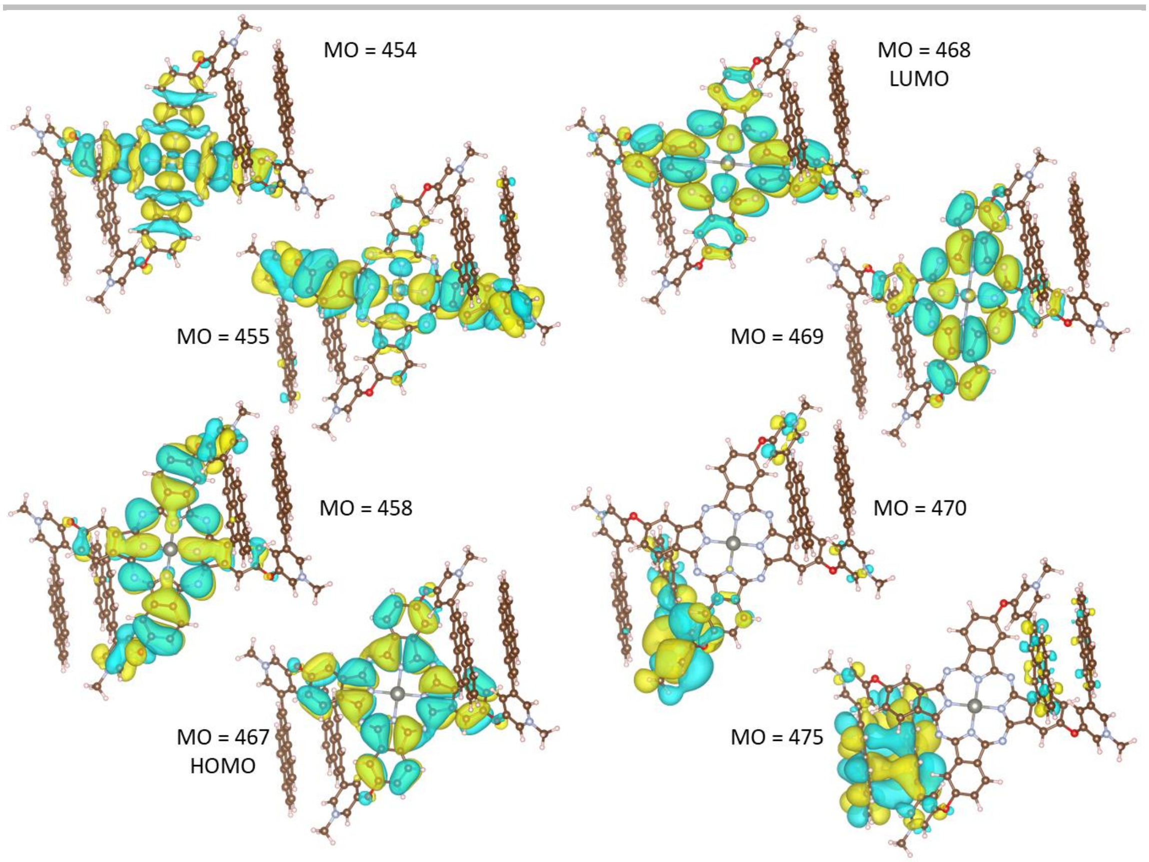

Figure S26. Selected molecular orbitals (isosurface value 0.01) for **1** involved in the first S_0-S_x vertical transitions. Calculation performed on the TD-DFT wB97xd/def2-SVP level of theory including water PCM solvation.

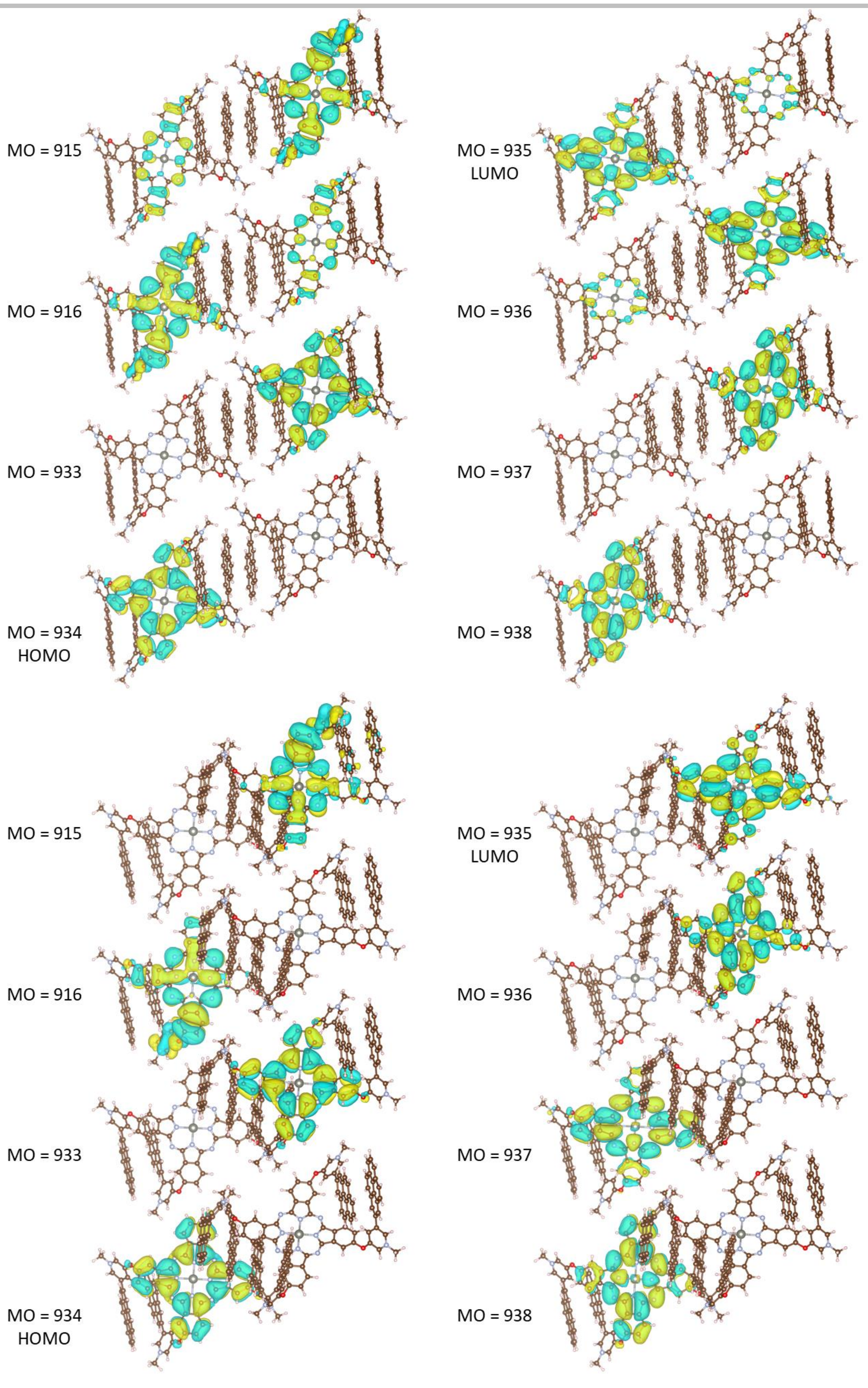

Figure S27. Selected molecular orbitals (isosurface value 0.01) for dimer A (top) and B (bottom) involved in the first S_0-S_x vertical transitions. Calculation performed on the TD-DFT wB97xd/def2-SVP level of theory including water PCM solvation.

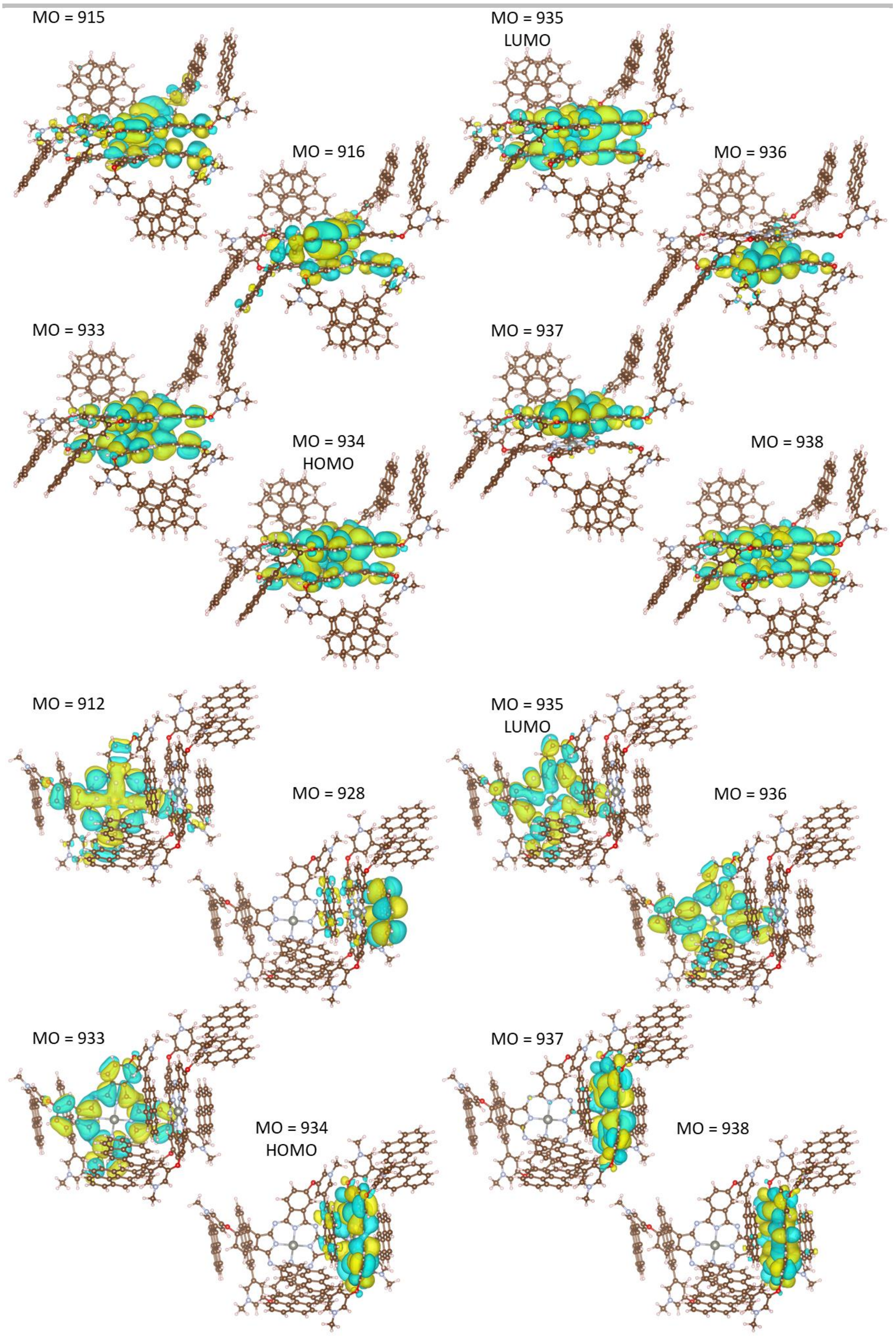

Figure S28. Selected molecular orbitals (isosurface value 0.01) for dimer C (top) and D (bottom) involved in the first S_0-S_x vertical transitions. Calculation performed on the TD-DFT wB97xd/def2-SVP level of theory including water PCM solvation.

Table S9. Vertical transition energies for **1** calculated with wB97xd/def2-SVP in DMSO PCM solvation. Contribution of initial and final molecular orbitals and the amplitude of contribution for the given transition.

	monomer					dimer A					dimer B					dimer C					dimer D				
	E	OSC	MO_i	MO_f	amp	E	OSC	MO_i	MO_f	amp	E	OSC	MO_i	MO_f	amp	E	OSC	MO_i	MO_f	amp	E	OSC	MO_i	MO_f	amp
S1	1.896	0.7834	458	469	0.12132	1.893	1.5641	916	938	0.11089	1.908	1.4074	916	938	-0.11244	1.784	0.0045	933	935	0.55796	1.829	0.4037	933	935	-0.1905
			467	468	0.68611			933	936	0.28285			933	935	-0.29078			933	937	-0.15619			934	937	0.56272
								934	935	0.60546			934	937	0.61392			934	937	-0.18483			934	938	-0.33774
								934	936	-0.12728								934	938	-0.321					
S2	1.921	0.6353	458	468	-0.12253	1.903	0.2133	915	937	0.10593	1.914	0.1381	915	936	-0.10313	1.794	0.0053	933	936	0.34319	1.847	0.2597	933	936	0.22402
			467	469	0.68654			933	935	0.12916			933	935	0.61087			933	937	-0.20546			934	937	0.32642
								933	936	0.60246			934	937	0.28881			933	938	0.11485			934	938	0.55471
								934	935	-0.28262			934	938	0.114			934	935	-0.26504					
																		934	936	-0.32421					
																		934	937	-0.33806					
																		934	938	0.11254					
S3	3.411	0.0000	454	468	-0.6353	1.924	0.4384	916	935	-0.12077	1.925	0.4348	915	935	0.1117	1.952	0.9224	933	936	0.21362	1.915	0.6489	912	936	-0.13549
			455	468	0.22904			934	938	0.67726			933	936	0.6752			933	938	-0.17096			933	935	0.6263
																		934	935	0.59391			933	936	-0.18711
																		934	937	-0.1442			934	937	0.17217
S4	3.428	0.0000	454	469	0.64476	1.928	0.6731	915	936	-0.11749	1.935	0.7460	916	937	0.1251	1.989	1.0903	933	935	0.2195	1.921	0.7267	912	935	0.13273
			455	469	-0.21399			933	937	0.67987			933	935	-0.11337			933	936	0.32845			933	935	0.18752
													934	938	0.67193			933	937	0.25804			933	936	0.61635
																		934	936	-0.28704			934	937	-0.11803
																		934	937	0.39193			934	938	-0.19946
S5	3.748	0.0165	460	475	-0.21734	3.473	0.0001	903	936	-0.34461	3.372	0.0010	928	935	0.35969	2.116	0.0228	933	936	0.44433	2.862	0.0069	928	937	0.36276
			460	477	0.10268			906	935	-0.10325			928	936	0.13682			934	936	0.5378			928	938	0.58609
			463	468	-0.13118			906	936	-0.48646			930	935	0.13774										
			463	470	0.16523			910	936	0.23962			932	935	0.52759										
			464	468	-0.1971								932	936	0.15092										
			464	470	0.25977																				
			464	477	0.10622																				
			464	481	0.14475																				
			464	487	-0.12713																				
			466	468	-0.18186																				
			466	470	0.22926																				
			466	480	0.11104																				
			466	481	0.11391																				
			466	487	-0.11054																				
S6	3.754	0.0109	459	474	0.13339	3.490	0.0001	894	937	-0.10502	3.389	0.0004	928	935	-0.10036	2.150	0.0275	933	937	0.57462	2.882	0.0088	925	937	-0.11614
			461	476	-0.11333			903	937	-0.36189			928	936	0.38637			934	937	-0.37132			926	937	-0.13426
			463	468	0.29266			906	937	-0.5086			930	936	0.13015								927	937	-0.17083
			463	471	-0.14977			910	937	0.22112			932	935	-0.17728								928	937	0.52372
			463	472	-0.10164								932	936	0.50593								928	938	-0.37015
			463	473	0.17036																				
			464	468	-0.15044																				
			464	471	0.1075																				
			464	472	0.11726																				
			465	468	0.20283																				
			465	471	-0.10022																				
			465	473	-0.11416																				
			465	476	-0.12986																				
			465	478	0.11381																				
			465	479	-0.10248																				

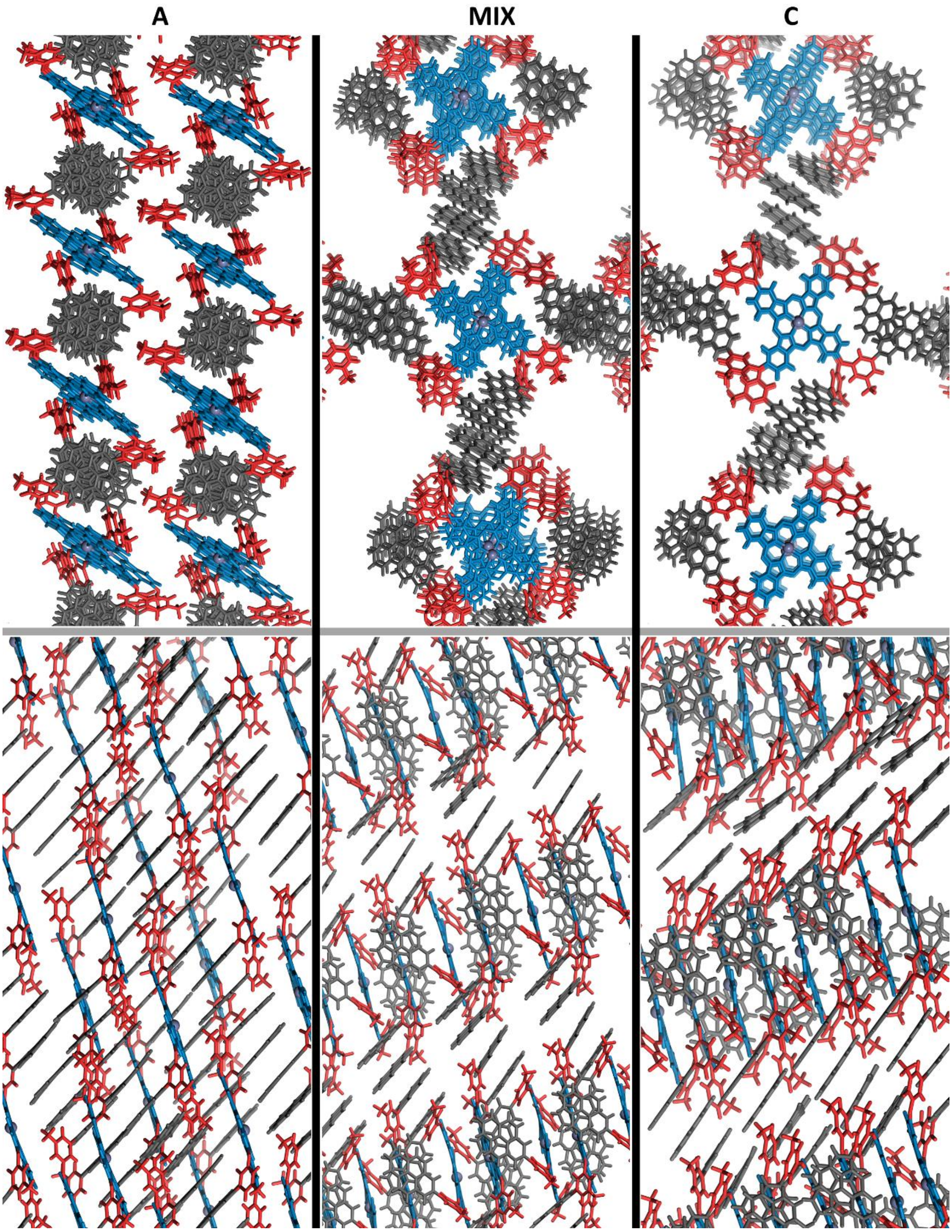

Figure S29. Top and side view of Dreiding forcefield optimized aggregates based on dimer A (A), dimer C (C) and a combination of both (MIX). For clarity, the ZnPcs are displayed in blue, pyrenes in grey and methylpyridinium linkers in red.

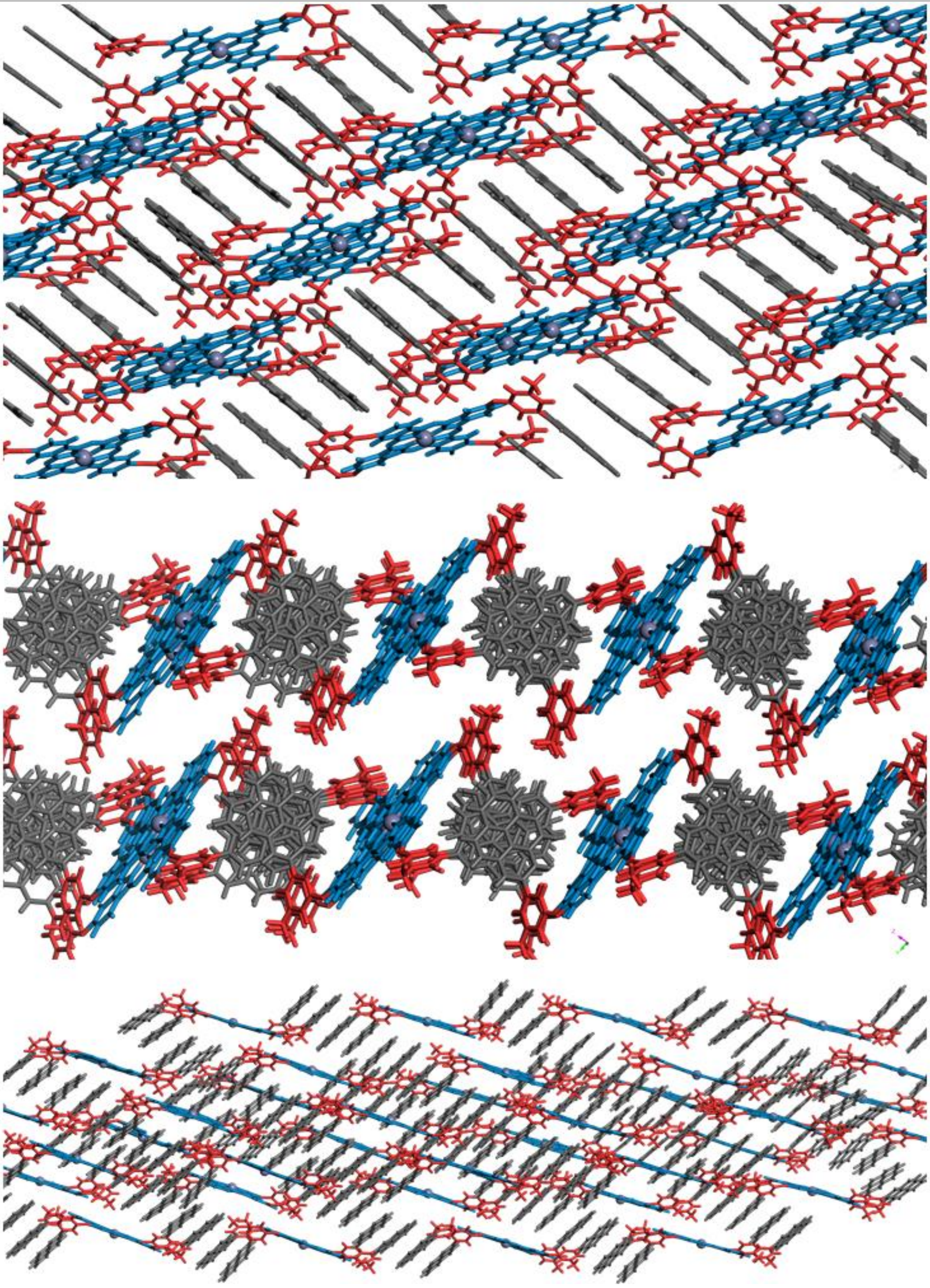

Figure S30. Top and side view of Dreiding forcefield optimized aggregate based on dimer A (A). For better visibility the ZnPc-cores are displayed in blue, pyrenes in grey and methylpyridinium linkers in red.

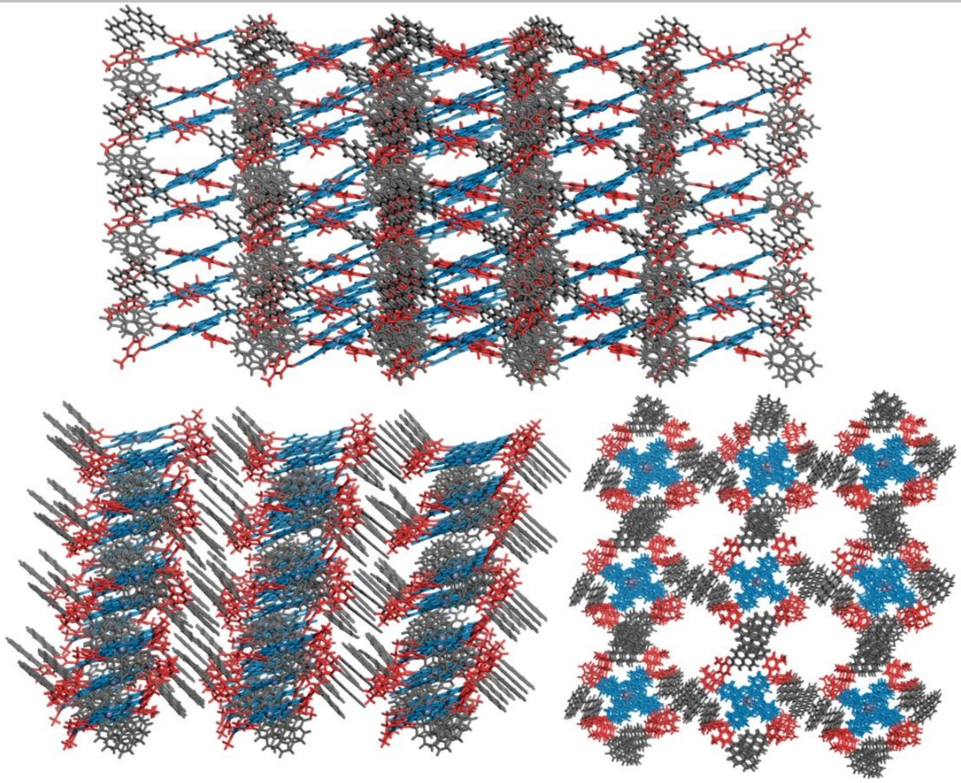

Figure S31. Top and side view of Dreiding forcefield optimized aggregate MIX. For better visibility the ZnPc-cores are displayed in blue, pyrenes in grey and methylpyridinium linkers in red.

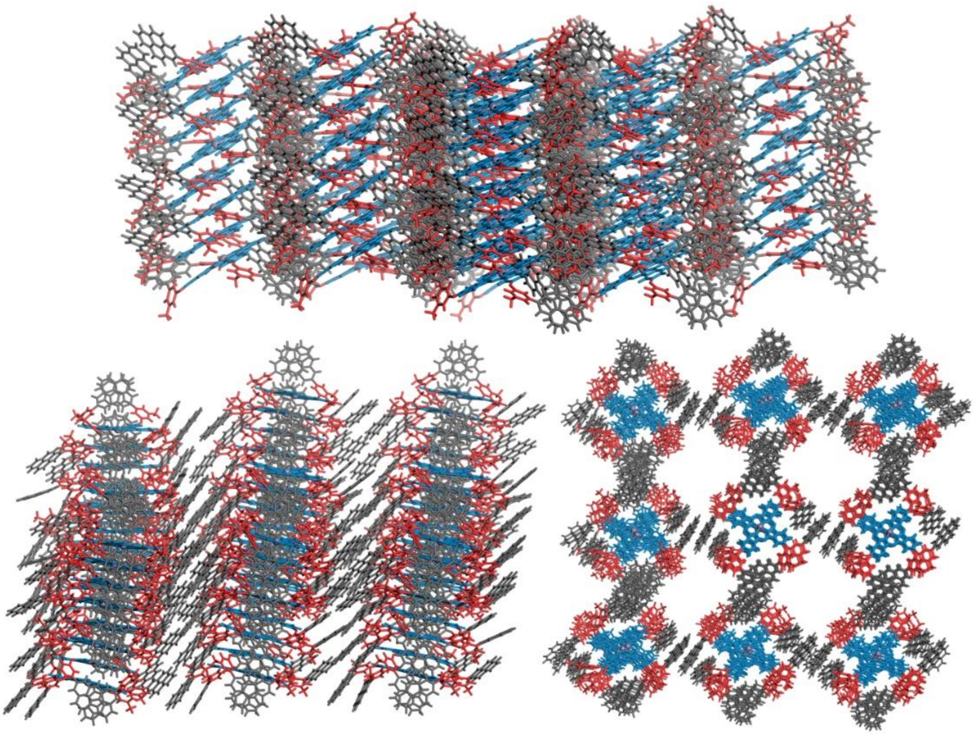

Figure S32. Top and side view of Dreiding forcefield optimized aggregate based on dimer C (C). For better visibility the ZnPc-cores are displayed in blue, pyrenes in grey and methylpyridinium linkers in red.

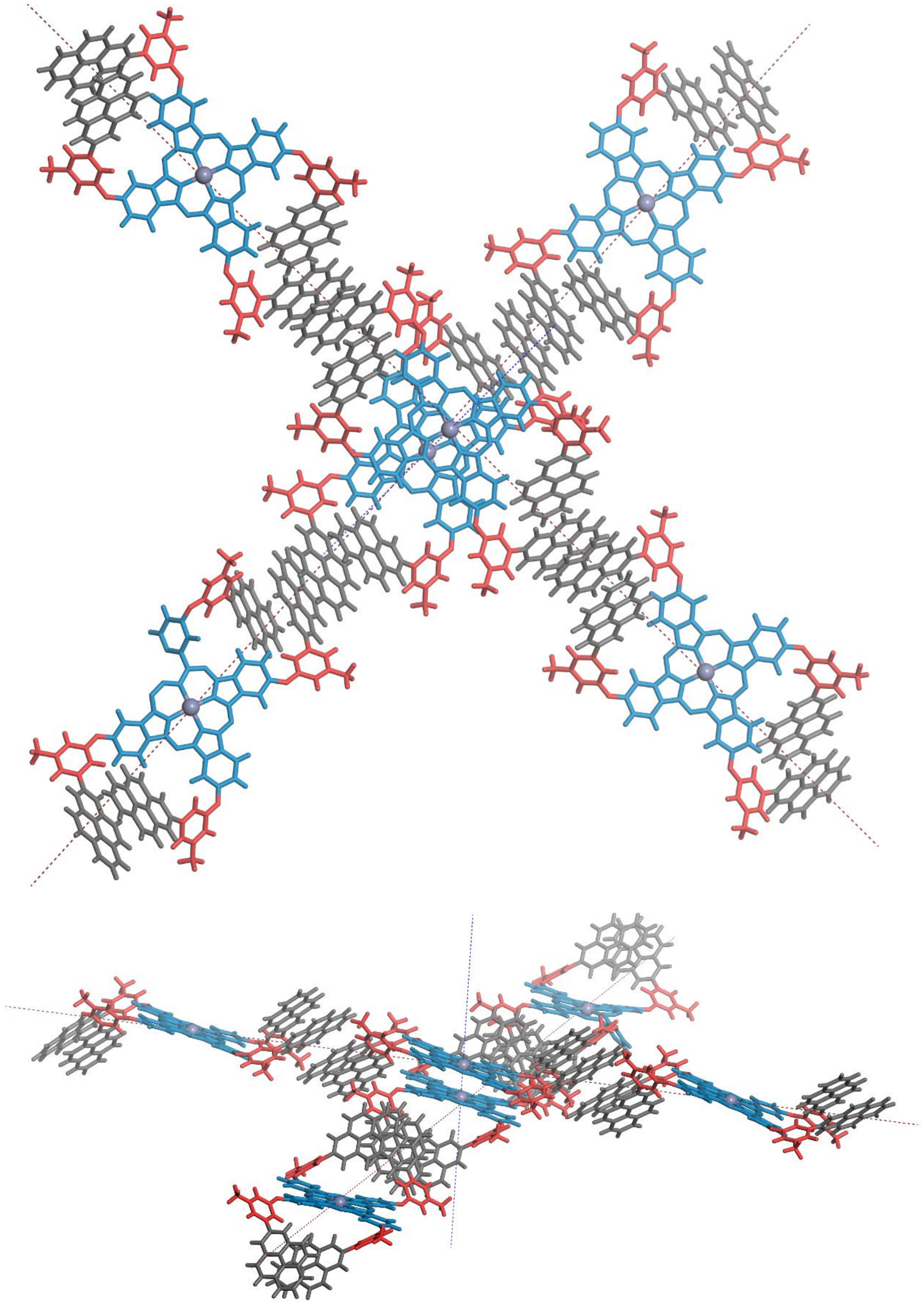

Figure S33. Top and side view of Dreiding forcefield optimized aggregate MIX. For better visibility only 2 layers are displayed - the ZnPc-cores are displayed in blue, pyrenes in grey and methylpyridinium linkers in red.

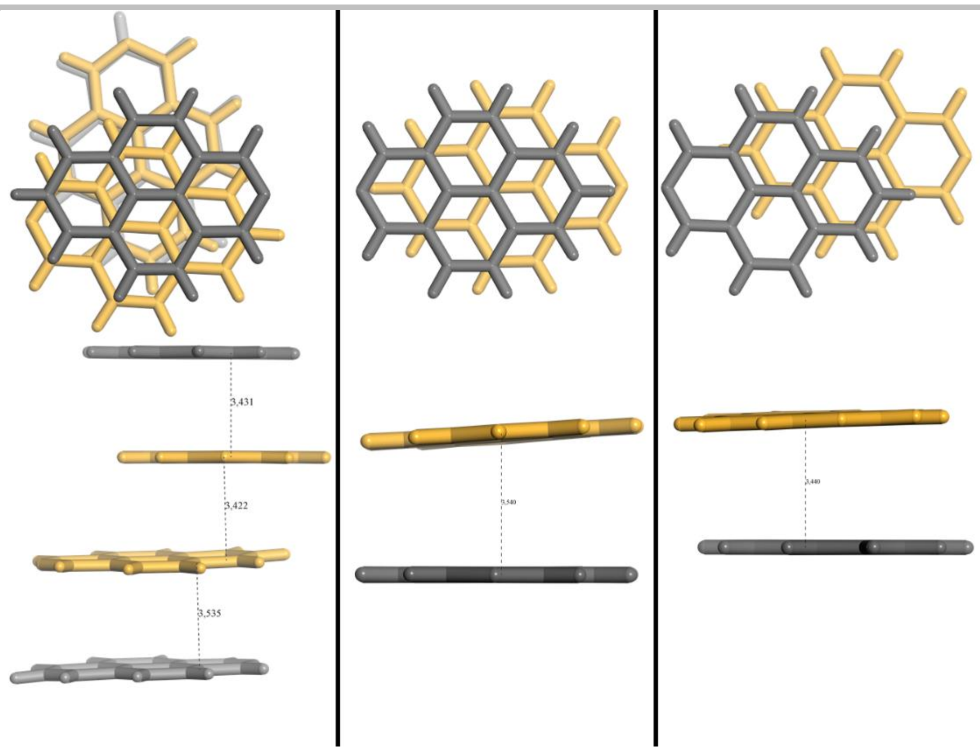

Figure S34. Pyrene interactions present in aggregate A (left), MIX (middle) and C (right). For better visibility, the ZnPc cores are not displayed. Pyrenes with the same color belong to the same ZnPc.

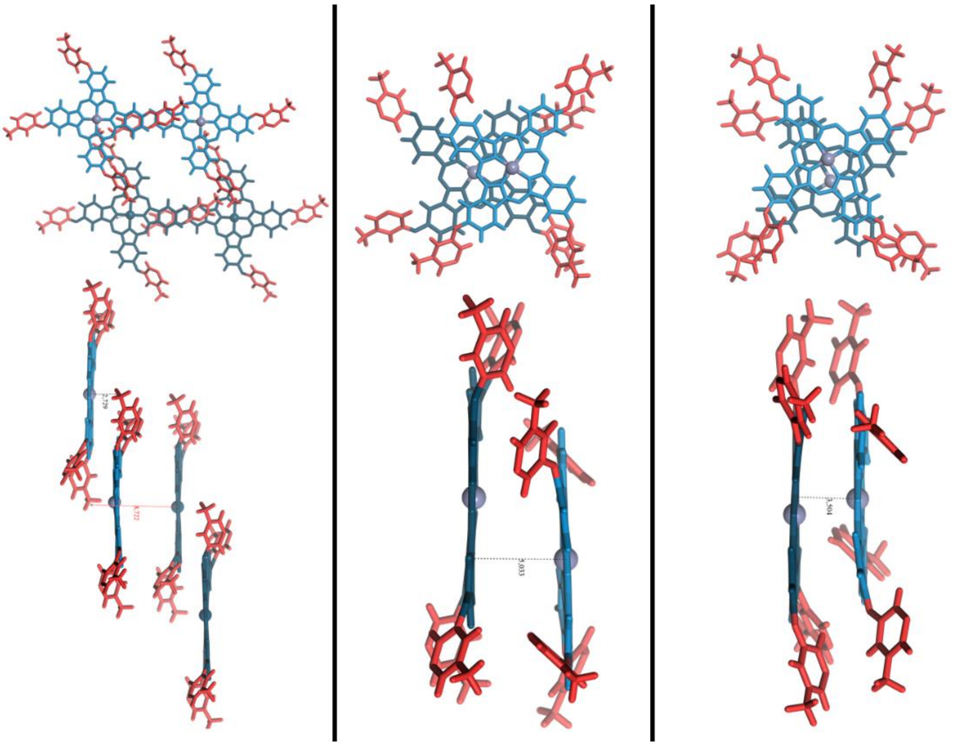

Figure S35. ZnPc interactions present in aggregate A (left), MIX (middle) and C (right). For better visibility, the pyrenes are not displayed.

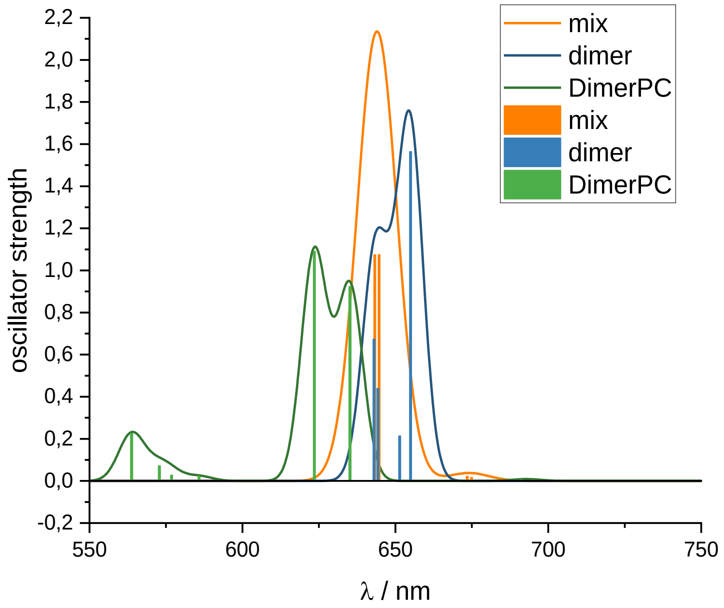

Figure S36. Vertical transitions calculated on the TD-DFT wB97xd/def2-SVP level of theory including water PCM solvation. Gaussian distribution with 10nm FWHM used to generate the spectra.

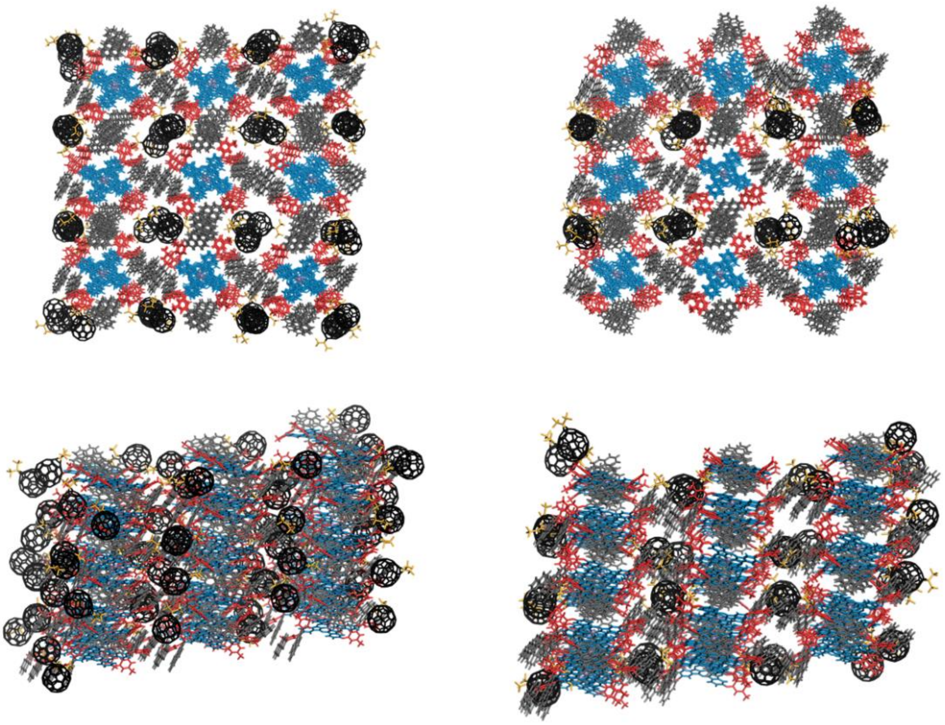

Figure S37. Dreiding forcefield predicted interaction motifs of 3 (left) and 4 (right) with MIX. Please note, the initial placement of 3 and 4 is arbitrary.

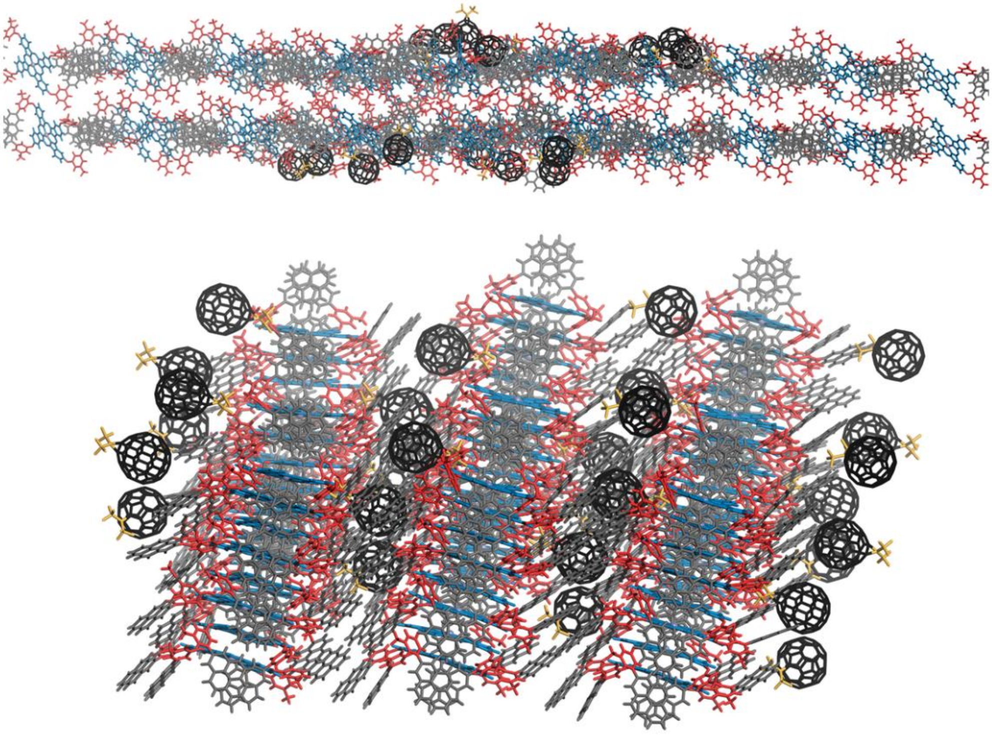

Figure S38. Dreiding forcefield predicted interaction motifs of 3 with A (top) and C (bottom). Please note, the initial placement of 3 is arbitrary.

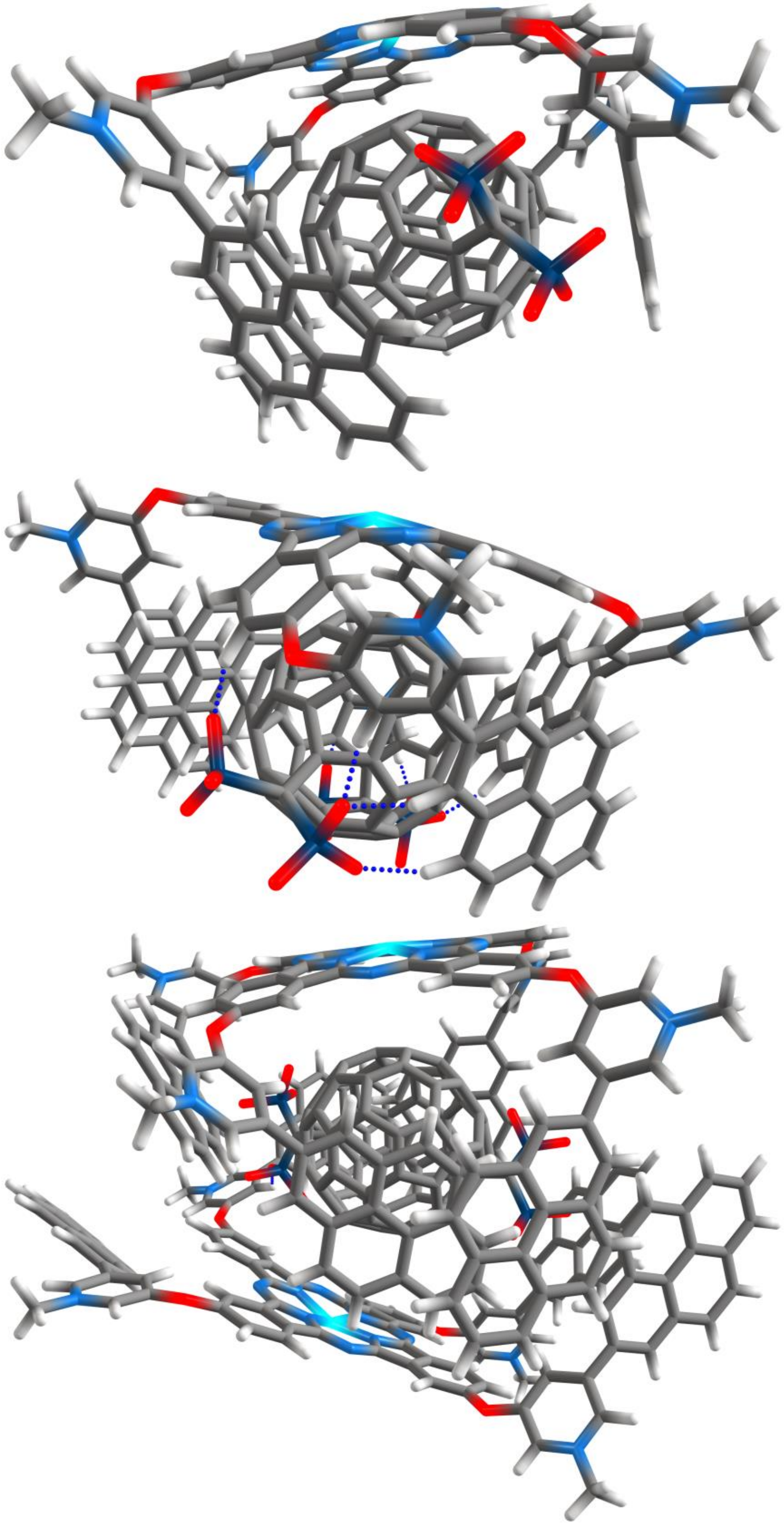

Figure S39. wB97xd/Lan2DZ optimized geometries for 1:1 complexation of **1** with **3** (top), **4** (middle) and 2:1 complexation of **1** with **4** (bottom). In case of phosphorus additional d-orbitals (Lan2DZdp) were added to the basis set to account for the hyper valence. The input structures are based on Dreiding simulated annealing calculations.

Spectroelectrochemical characterisation

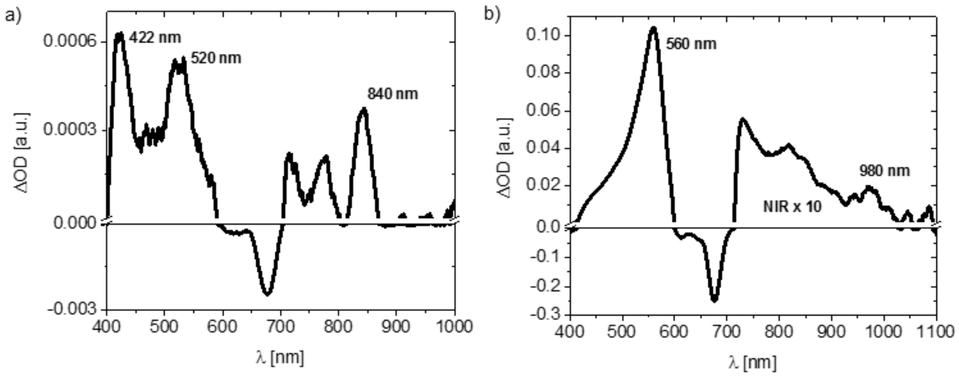

Figure S40. Differential absorption spectrum obtained upon spectroelectrochemical a) oxidation and b) reduction of **1** in DMSO. A voltage of +1.2 V *vs.* Ag-wire (Baseline set to +1.0 V) and -0.2 V *vs.* Ag-wire (Baseline set to 0.0 V) for oxidation and reduction, respectively, were applied.

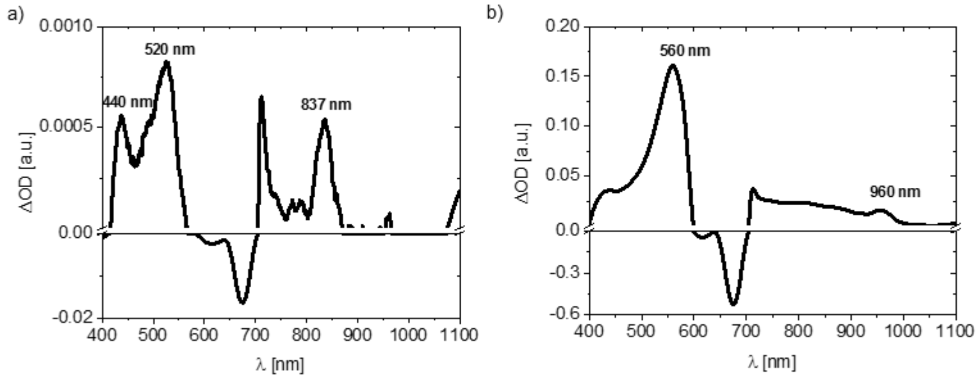

Figure S41. Differential absorption spectrum obtained upon spectroelectrochemical a) oxidation and b) reduction of **2** in DMSO. A voltage of +1.0 V vs. Ag-wire (Baseline set to +0.9 V) and -0.2 V vs. Ag-wire (Baseline set to 0.2 V) for oxidation and reduction, respectively, were applied.

SUPPORTING INFORMATION

Time resolved transient absorption spectroscopy

Measurements in DMSO

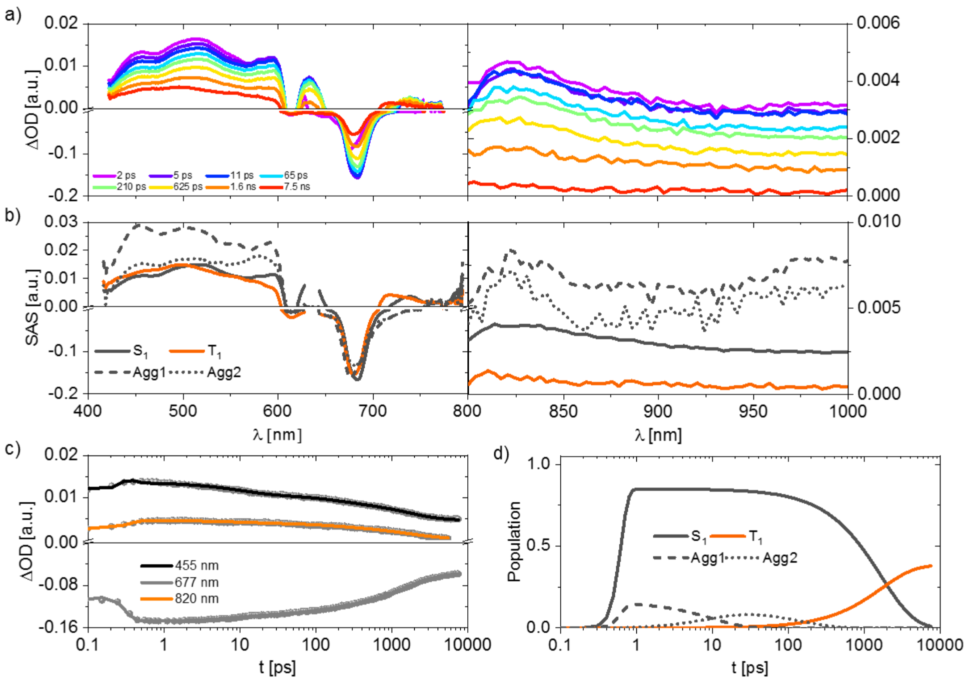

Figure S42. Differential absorption spectra obtained in DMSO upon femtosecond pump-probe experiments (λ_{ex} = 630 nm) (**a**) and time profiles of selected wavelengths and corresponding fittings of GloTarAn (**c**) of **1** (c = 2 x 10^{-5} M) at time delays from 0-7.5 ns. Species associated differential absorption spectra (**b**) and population over time (**d**) of **1** obtained upon deconvolution of spectra with Target Analysis (GloTarAn).

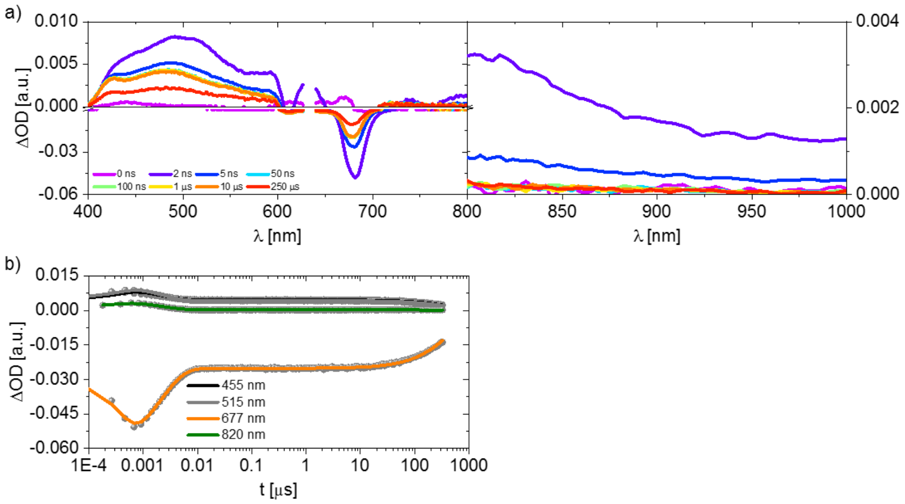

Figure S43. Differential absorption spectra obtained in DMSO upon nanosecond pump-probe experiments (λ_{ex} = 630 nm) (**a**) and time profiles of selected wavelengths and corresponding fittings of GloTarAn (**b**) of **1** (c = 2 x 10^{-5} M) at time delays from 0-250 µs.

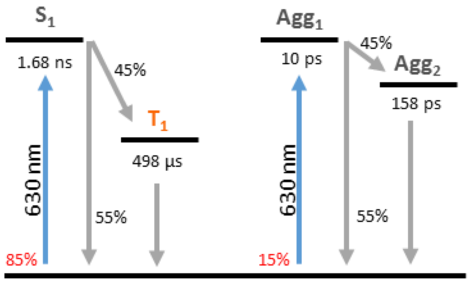

Figure S44. Mechanistic model used in Target Analysis (GloTarAn) for deconvolution of spectra obtained upon femto- and nanosecond pump-probe experiments (λ_{ex} = 630 nm) of **1** (c = 2 x 10^{-5} M) in DMSO at time delays from 0-250 µs.

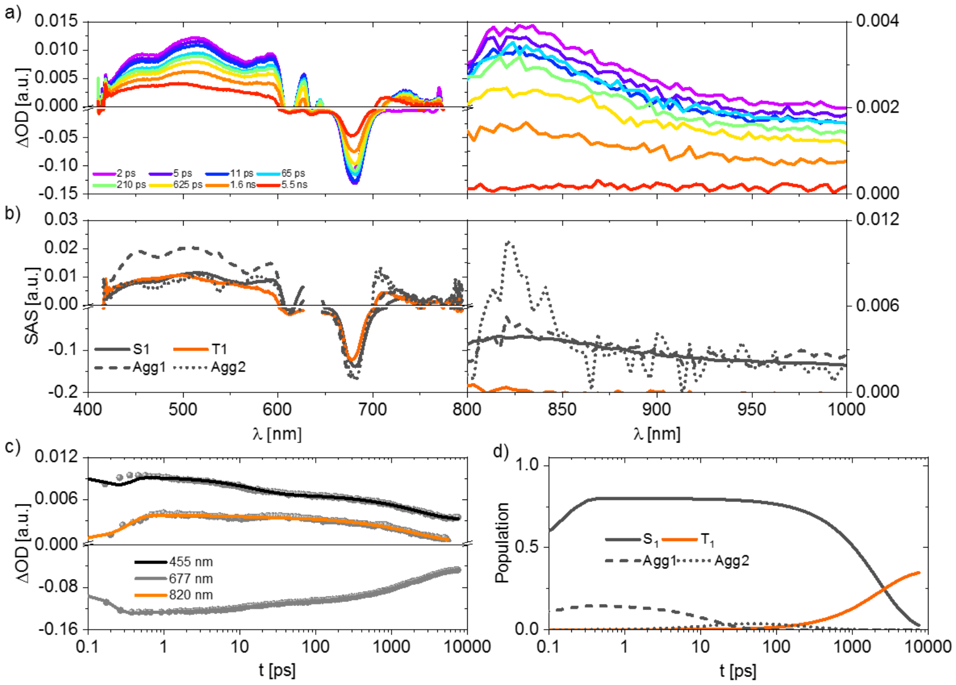

Figure S45. Differential absorption spectra obtained in DMSO upon femtosecond pump-probe experiments (λ_{ex} = 630 nm) (**a**) and time profiles of selected wavelengths and corresponding fittings of GloTarAn (**c**) of **2** (c = 2 x 10^{-5} M) at time delays from 0-7.5 ns. Species associated differential absorption spectra (**b**) and population over time (**d**) of **2** obtained upon deconvolution of spectra with Target Analysis (GloTarAn).

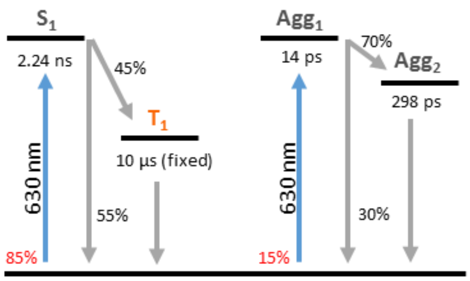

Figure S46. Mechanistic model used in Target Analysis (GloTarAn) for deconvolution of spectra obtained upon femtosecond pump-probe experiments (λ_{ex} = 630 nm) of **2** (c = 2 x 10^{-5} M) in DMSO at time delays from 0-7.5 ns (T_1 fixed at 10 µs).

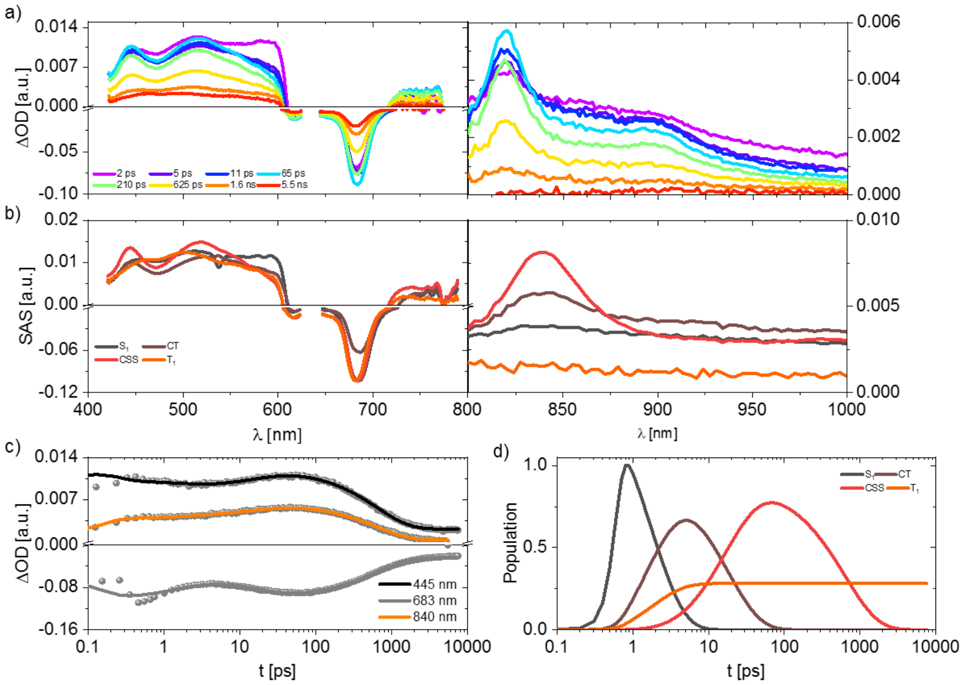

Figure S47. Differential absorption spectra obtained in DMSO upon femtosecond pump-probe experiments (λ_{ex} = 630 nm) (**a**) and time profiles of selected wavelengths and corresponding fittings of GloTarAn (**c**) of **1-4** (c = 2 x 10^{-5} M, 1:2 ratio) at time delays from 0-5.5 ns. Species associated differential absorption spectra (**b**) and population over time (**d**) of **1-4** obtained upon deconvolution of spectra with Target Analysis (GloTarAn).

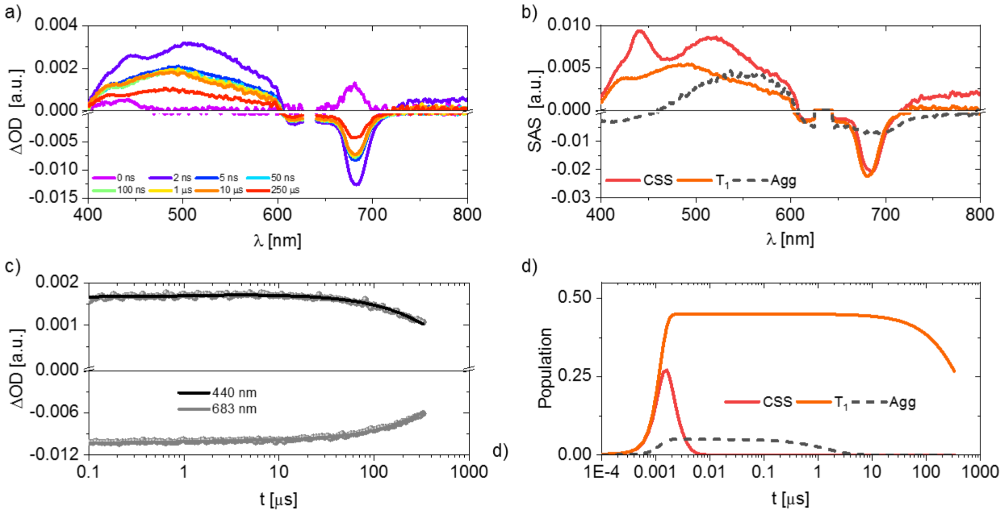

Figure S48. Differential absorption spectra obtained in DMSO upon nanosecond pump-probe experiments (λ_{ex} = 630 nm) (**a**) and time profiles of selected wavelengths and corresponding fittings of GloTarAn (**c**) of **1-4** (c = 2 x 10^{-5} M, 1:2 ratio) at time delays from 0-250 µs. Species associated differential absorption spectra (**b**) and population over time (**d**) of **1-4** obtained upon deconvolution of spectra with Target Analysis (GloTarAn).

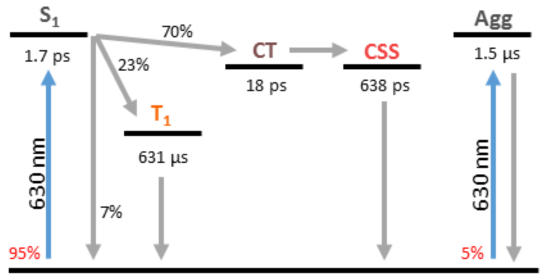

Figure S49. Mechanistic model used in Target Analysis (GloTarAn) for deconvolution of spectra obtained upon femto- and nanosecond pump-probe experiments (λ_{ex} = 630 nm) of **1-4** (c = 2 x 10^{-5} M, 1:2 ratio) in DMSO at time delays from 0-250 µs.

Measurements in regime A

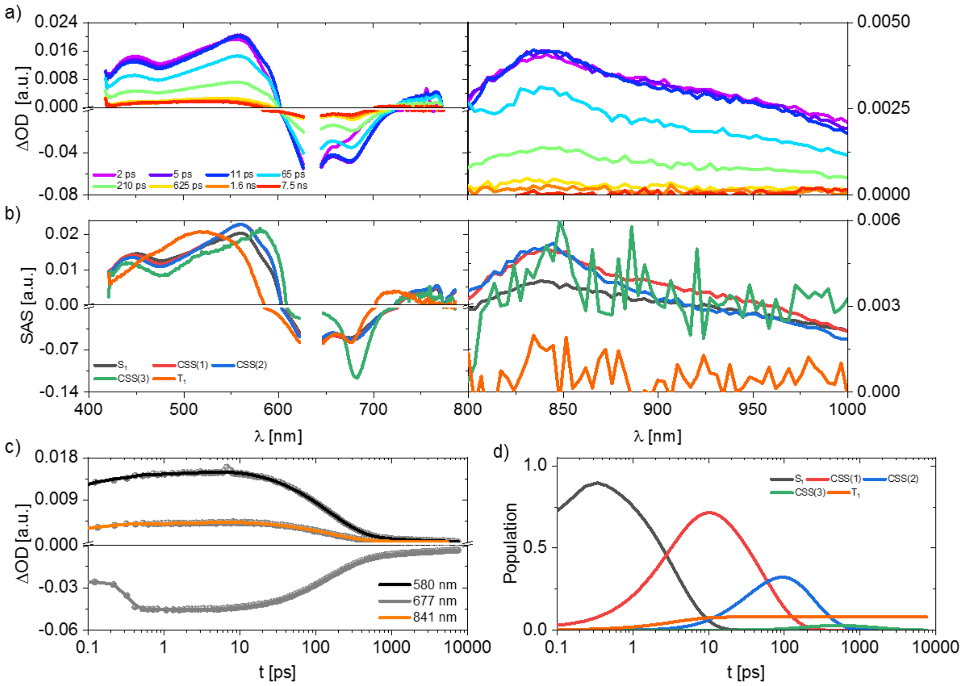

Figure S50. Differential absorption spectra obtained in 40 vol% DMSO/60 vol% water upon femtosecond pump-probe experiments (λ_{ex} = 630 nm) (**a**) and time profiles of selected wavelengths and corresponding fittings of GloTarAn (**c**) of $\mathbf{1}_{\mathbf{aggA}}$ (c = 2 x 10^{-5} M) at time delays from 0-7.5 ns. Species associated differential absorption spectra (**b**) and population over time (**d**) of $\mathbf{1}_{\mathbf{aggA}}$ obtained upon deconvolution of spectra with Target Analysis (GloTarAn).

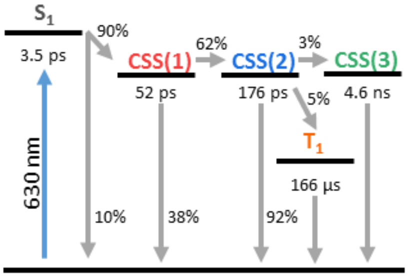

Figure S51. Mechanistic model used in Target Analysis (GloTarAn) for deconvolution of spectra obtained upon femto- and nanosecond pump-probe experiments (λ_{ex} = 630 nm) of $\mathbf{1}_{\mathbf{aggA}}$ (c = 2 x 10^{-5} M) in 40 vol% DMSO/60 vol% water at time delays from 0-250 µs.

Measurements in regime B

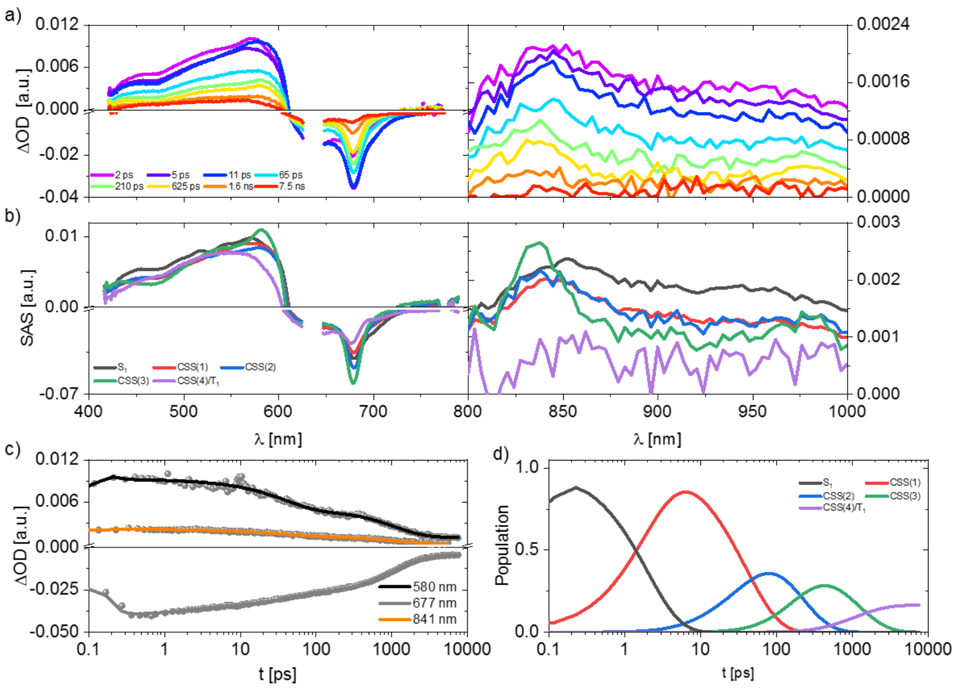

Figure S52. Differential absorption spectra obtained in 5 vol% DMSO/95 vol% water upon femtosecond pump-probe experiments (λ_{ex} = 630 nm) (**a**) and time profiles of selected wavelengths and corresponding fittings of GloTarAn (**c**) of $\mathbf{1}_{\mathbf{aggB}}$ (c = 2 x 10^{-5} M) at time delays from 0-7.5 ns. Species associated differential absorption spectra (**b**) and population over time (**d**) of $\mathbf{1}_{\mathbf{aggB}}$ obtained upon deconvolution of spectra with Target Analysis (GloTarAn).

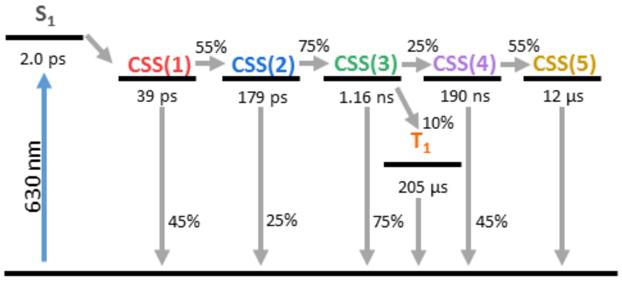

Figure S53. Mechanistic model used in Target Analysis (GloTarAn) for deconvolution of spectra obtained upon femto- and nanosecond pump-probe experiments (λ_{ex} = 630 nm) of $\mathbf{1}_{\mathbf{aggB}}$ (c = 2 x 10^{-5} M) in 5 vol% DMSO/95 vol% water at time delays from 0-250 µs.

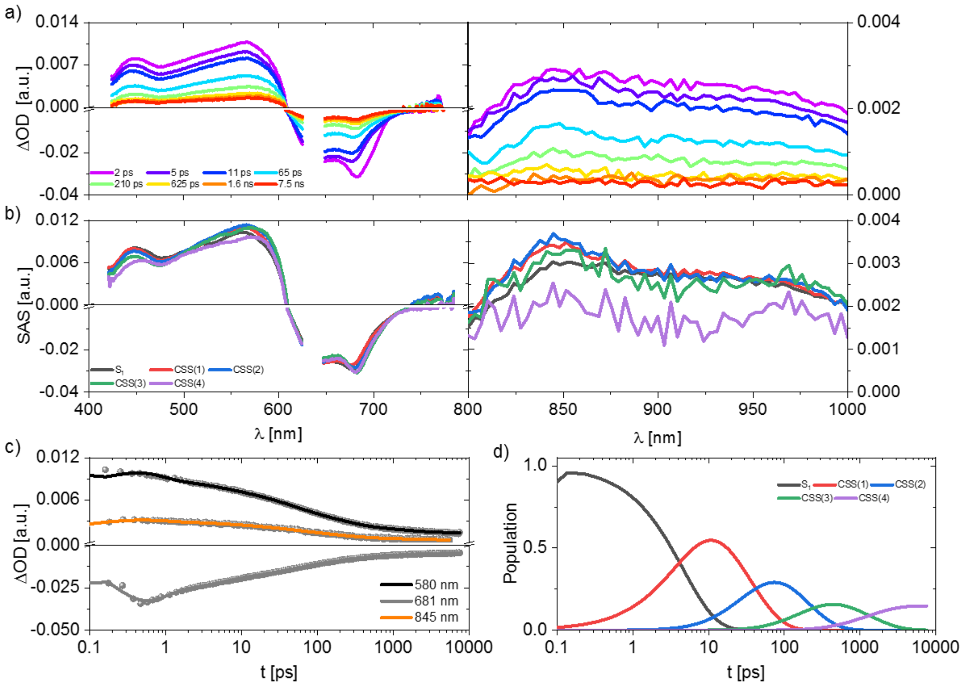

Figure S54. Differential absorption spectra obtained in 5 vol% DMSO/95 vol% water upon femtosecond pump-probe experiments (λ_{ex} = 630 nm) (**a**) and time profiles of selected wavelengths and corresponding fittings of GloTarAn (**c**) of **1_{aggB}-4** (c = 2 x 10^{-5} M, 1:2 ratio) at time delays from 0-7.5 ns. Species associated differential absorption spectra (**b**) and population over time (**d**) of **1_{aggB}-4** obtained upon deconvolution of spectra with Target Analysis (GloTarAn).

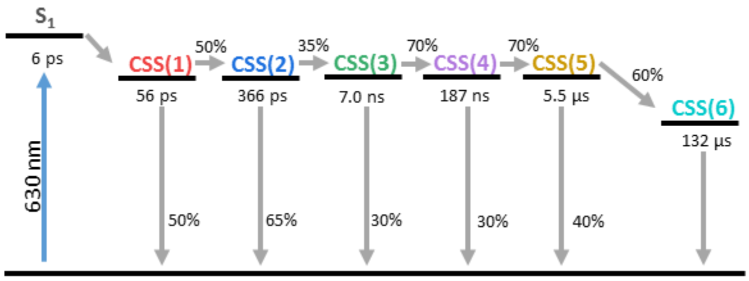

Figure S55. Mechanistic model used in Target Analysis (GloTarAn) for deconvolution of spectra obtained upon femto- and nanosecond pump-probe experiments (λ_{ex} = 630 nm) of **ZnPc(Pyr)$_{aggB}$|C$_{60}$(2)** (c = 2 x 10^{-5} M, 1:2 ratio) in 5 vol% DMSO/95 vol% water at time delays from 0-250 µs.

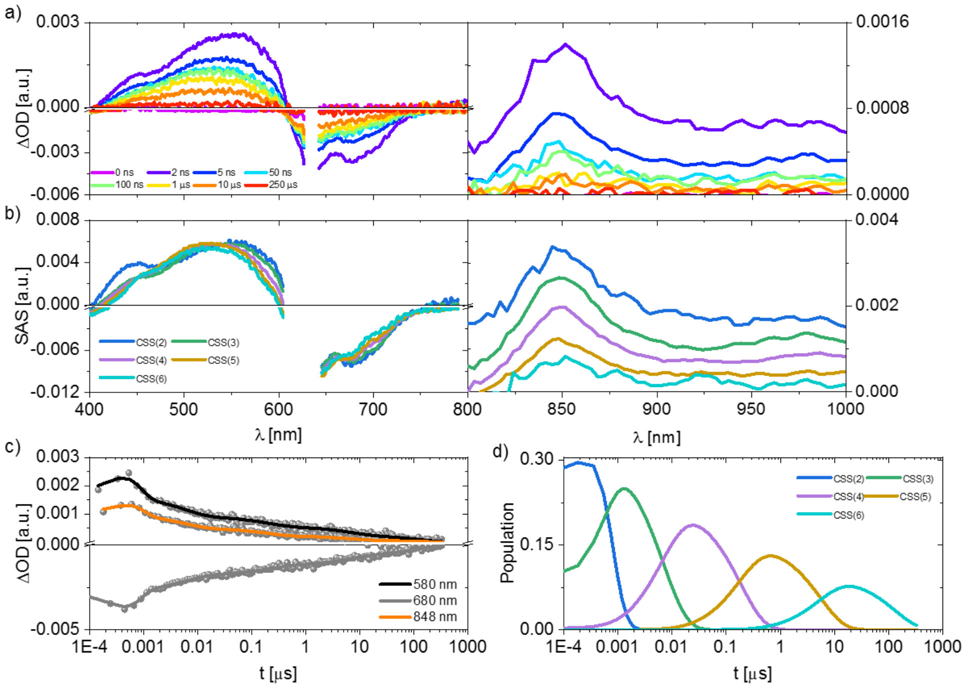

Figure S56. Differential absorption spectra obtained in 5 vol% DMSO/95 vol% water upon nanosecond pump-probe experiments (λ_{ex} = 630 nm) (**a**) and time profiles of selected wavelengths and corresponding fittings of GloTarAn (**c**) of **ZnPc(Pyr)$_{aggB}$|C$_{60}$(2)** (c = 2 x 10^{-5} M, 1:1 ratio) at time delays from 0-250 µs. Species associated differential absorption spectra (**b**) and population over time (**d**) of **ZnPc(Pyr)$_{aggB}$|C$_{60}$(2)** obtained upon deconvolution of spectra with Target Analysis (GloTarAn).

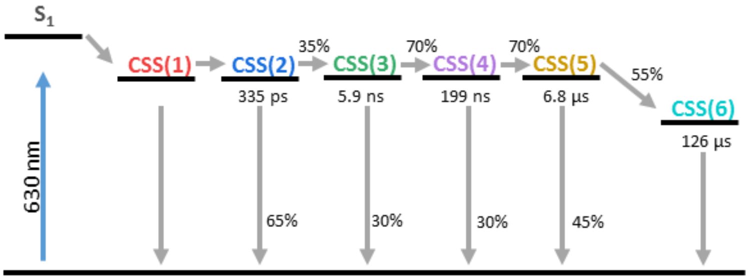

Figure S57. Mechanistic model used in Target Analysis (GloTarAn) for deconvolution of spectra obtained upon nanosecond pump-probe experiments (λ_{ex} = 630 nm) of **1$_{aggB}$-4** (c = 2 x 10^{-5} M, 1:1 ratio) in 5 vol% DMSO/95 vol% water at time delays from 0-250 µs.

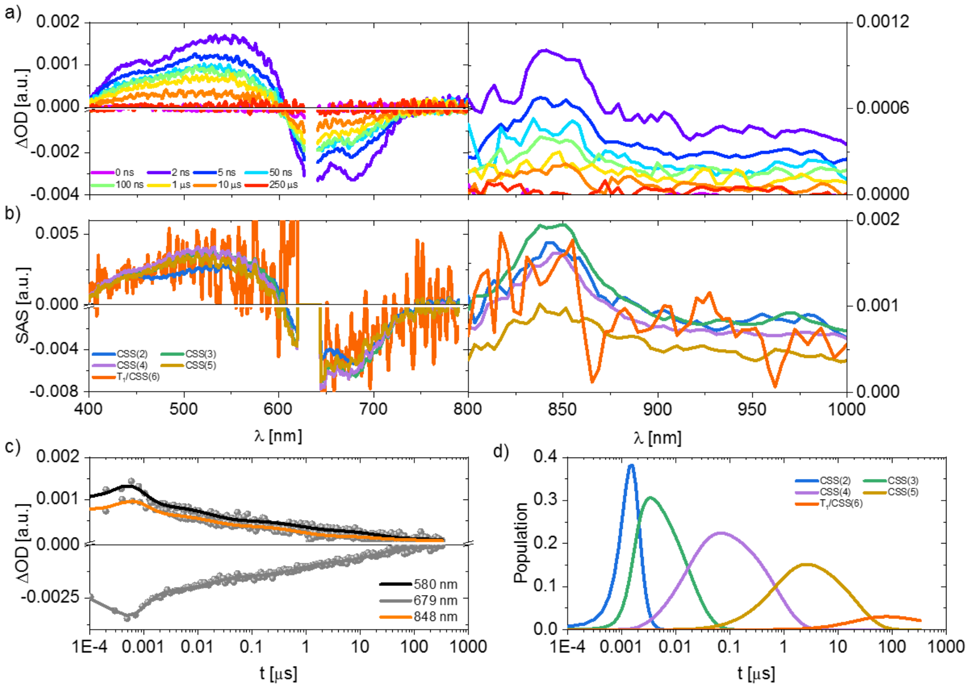

Figure S58. Differential absorption spectra obtained in 5 vol% DMSO/95 vol% water upon nanosecond pump-probe experiments (λ_{ex} = 630 nm) (**a**) and time profiles of selected wavelengths and corresponding fittings of GloTarAn (**c**) of **1_{aggB}-4** (c = 2 x 10^{-5} M, 1:0.5 ratio) at time delays from 0-250 µs. Species associated differential absorption spectra (**b**) and population over time (**d**) of **1_{aggB}-4** obtained upon deconvolution of spectra with Target Analysis (GloTarAn).

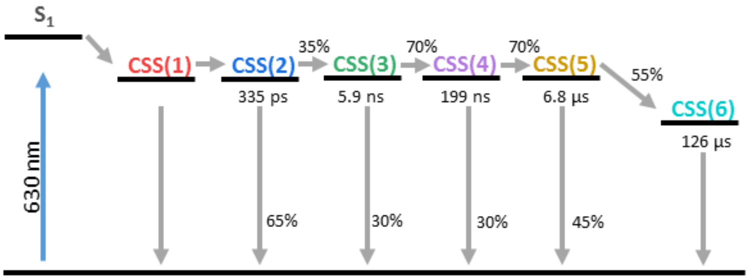

Figure S59. Mechanistic model used in Target Analysis (GloTarAn) for deconvolution of spectra obtained upon nanosecond pump-probe experiments (λ_{ex} = 630 nm) of **1_{aggB}-4** (c = 2 x 10^{-5} M, 1:0.5 ratio) in 5 vol% DMSO/95 vol% water at time delays from 0-250 µs.

References

[1] M. Beinhoff, W. Weigel, M. Jurczok, W. Rettig, C. Modrakowski, I. Brüdgam, H. Hartl, A. D. Schlüter, *European J. Org. Chem.* **2001**, *49*, 3819–3829.
[2] H. Li, T. J. Jensen, F. R. Fronczek, M. G. H. Vicente, **2008**, *32*, 502–511.
[3] B. Iorga, P. Savignac, *J. Organomet. Chem.* **2001**, *624*, 203–207.
[4] A. L. Mirakyan, L. J. Wilson, *J. Chem. Soc. Perkin Trans. 2* **2002**, *0*, 1173–1176.
[5] Mayo, S. L.; Olafson, B. D.; Goddard, W. A., III. *J. Phys. Chem.* **1990**, *94*, 8897-8909.
[6] Dassault Systèmes BIOVIA, [Materials Studio 2018], [18.1.0.2017], San Diego: Dassault Systèmes, (2018).
[7] Gaussian 16, Revision C.01, M. J. Frisch, G. W. Trucks, H. B. Schlegel, G. E. Scuseria, M. A. Robb, J. R. Cheeseman, G. Scalmani, V. Barone, G. A. Petersson, H. Nakatsuji, X. Li, M. Caricato, A. V. Marenich, J. Bloino, B. G. Janesko, R. Gomperts, B. Mennucci, H. P. Hratchian, J. V. Ortiz, A. F. Izmaylov, J. L. Sonnenberg, D. Williams-Young, F. Ding, F. Lipparini, F. Egidi, J. Goings, B. Peng, A. Petrone, T. Henderson, D. Ranasinghe, V. G. Zakrzewski, J. Gao, N. Rega, G. Zheng, W. Liang, M. Hada, M. Ehara, K. Toyota, R. Fukuda, J. Hasegawa, M. Ishida, T. Nakajima, Y. Honda, O. Kitao, H. Nakai, T. Vreven, K. Throssell, J. A. Montgomery, Jr., J. E. Peralta, F. Ogliaro, M. J. Bearpark, J. J. Heyd, E. N. Brothers, K. N. Kudin, V. N. Staroverov, T. A. Keith, R. Kobayashi, J. Normand, K. Raghavachari, A. P. Rendell, J. C. Burant, S. S. Iyengar, J. Tomasi, M. Cossi, J. M. Millam, M. Klene, C. Adamo, R. Cammi, J. W. Ochterski, R. L. Martin, K. Morokuma, O. Farkas, J. B. Foresman, and D. J. Fox, Gaussian, Inc., Wallingford CT, 2016.
[8] Tomasi, J.; Mennucci, B.; and Cammi, R. *Chem. Rev.* **2005**, *105*, 2999-3093.
[9] Miertuš, S.; Scrocco, B.; and Tomasi, J. *Chem. Phys.* **1981**, *55*, 117-29.
[10] Pascual-Ahuir, J. L.; Silla, E.; and Tuñón, I. *J. Comp. Chem.* **1994**, *15*, 1127-38.
[11] Cossi, M.; Rega, N.; Scalmani, G.; and Barone, V. *J. Comp. Chem.* **2003**, *24*, 669-81.
[12] Lipparini, F.; Scalmani, G.; Mennucci, B.; Cances, E.; Caricato, M.; Frisch, M. J., *J. Chem. Phys.* **2010**, *133*, 014106.
[13] Caricato, M. *J. Chem. Theory & Comput.* **2012**, *8*, 4494.
[14] Scalmani, G.; and Frisch, M. J. *J. Chem. Phys.* **2010**, *132*, 114110.
[15] Chai, J.-D.; and Head-Gordon, M. *Phys. Chem. Chem. Phys.*, **2008**, *10*, 6615.
[16] Benjamin P. Pritchard, Doaa Altarawy, Brett Didier, Tara D. Gibson, Theresa L. Windus. *J. Chem. Inf. Model.* **2019**, *59*, 4814.
[17] Gordon, M. S., Binkley, J. S., Pople, J. A., Pietro, W. J. & Hehre, W. J. *J. Am. Chem. Soc.* **1982**, *104*, 2797.
[18] Dobbs, K. D. & Hehre, W. J. *J. Comput. Chem.* **1986**, *7*, 359–378.
[19] Check, C. E. et al. *J. Phys. Chem. A* **2001**, *105*, 8111–8116.
[20] Feller, D. *J. Comput. Chem.* **1996**, *17*, 1571–1586.
[21] Hay, P. J. & Wadt, W. R. *J. Chem. Phys*. **1985**, *82*, 270–283.
[22] Rappoport, D. & Furche, F. *J. Chem. Phys.* **2010**, *133*, 134105.
[23] Schuchardt, K. L. et al. *J. Chem. Inf. Model.* **2007**, *47*, 1045–1052.
[24] Wadt, W. R. & Hay, P. J. *J. Chem. Phys.* **1985**, *82*, 284–298.
[25] Weigend, F. & Ahlrichs, R. *Phys. Chem. Chem. Phys.* **2005**, *7*, 3297–3305.

Author Contributions

The manuscript was written through contributions of all authors. All authors have given approval to the final version of the manuscript.

E. Anaya-Plaza wrote the original draft and performed the synthesis and characterization of the molecules (supervised by T. Torres and A. de la Escosura), as well as the early titrations and photophysical investigations (supervised by D. M. Guldi, S. Bauroth and M. Sekita). J. Joseph finished the draft with his own contributions in terms of TEM images and a complete photophysical investigation, including transient absorption spectroscopy (supervised by D. M. Guldi) and added/supervised contributions from S. Bauroth (calculations, supervised by T. Clark), M. Wagner (light scattering, supervised by F. Gröhn) and C. Dolle (SAED, supervised by E. Spiecker). A. de la Escosura, D. M. Guldi and T. Torres designed the project and gathered funding.
